# Supplementary material for: Micronutrient intake status and associated factors among children aged 6–23 months in the emerging regions of Ethiopia: A multilevel analysis of the 2016 Ethiopia demographic and health survey
Source: PLoS One. 2021 Oct 22;16(10):e0258954. doi: 10.1371/journal.pone.0258954 (PMC8535338; doi:10.1371/journal.pone.0258954)
Supplement: Attachment — Submitted filename: mcn13041 MB Final.pdf [file pone.0258954.s002.pdf]

PLOS ONE

Micronutrient intake status and associated factors among children aged 6-23 months in the emerging regions of Ethiopia: a multilevel analysis of the 2016 Ethiopian demographic and health survey

--Manuscript Draft--

Manuscript Number: PONE-D-20-13718R2

Article Type: Research Article

Full Title: Micronutrient intake status and associated factors among children aged 6-23 months in the emerging regions of Ethiopia: a multilevel analysis of the 2016 Ethiopian demographic and health survey

Short Title: Micronutrient intake status and its associated factors among children aged 6-23 months

Corresponding Author: Tsegaye Gebremedhin
University of Gondar
Gondar, Amara ETHIOPIA

Keywords: Micronutrient intake; vitamins and minerals; Children; emerging regions; multilevel mixed-effect regression; Ethiopia

Abstract:

Background: Micronutrient deficiency is recognized as a major public health problem in developing countries, including Ethiopia. The scarcity of micronutrients, particularly in pastoral communities of Ethiopia, might be severe due to poor health care access, drought and poverty. However, empirical evidence is scarce in Ethiopia. Therefore, this study aimed to assess the micronutrient intake status of children aged 6 to 23 months in the emerging regions of Ethiopia.

Methods: Data from the 2016 Ethiopia Demographic and Health Survey (EDHS) were used. A two-stage stratified sampling technique was employed to identify 1009 children aged 6 to 23 months. A multilevel mixed-effect logistic regression analysis was used to identify individual and community-level factors associated with micronutrient intake status. In the final model, variables with a p-value of < 0.05 and adjusted odds ratio (AOR) with 95% confidence interval (CI) were used to identify factors statistically associated with micronutrient intake status.

Results: Overall, 62.7% (95% CI: 59.7-65.7) of children aged 6 to 23 months had received at least one of the recommended micronutrients. Antenatal care (ANC) visit (AOR: 1.95, 95% CI: 1.37-2.77), work in the agriculture (AOR: 2.22, 95% CI: 1.30-3.80) and children aged 13 to 23 months (AOR: 1.72, 95% CI: 1.25-2.36) were the individual-level factors, whereas rural residence (AOR: 0.37, 95% CI: 0.14-0.88), reside in Benishangul (AOR: 2.25, 95% CI: 1.29-4.93) and Gambella regions (AOR: 1.87, 95% CI: 1.03-3.38) were the community-level factors associated with micronutrient intake status.

Conclusions: The micronutrient intake status in the study area was low compared to the national recommendation. Promoting vitamin A and iron-rich foods and micronutrient powders at individual and community-level and strengthen supplementations and deworming alongside the community-based maternal and child health services would improve the micronutrient intake among children.

Order of Authors:

Tsegaye Gebremedhin
Andualem Yalew Aschalew
Chalie Tadie
Endalkachew Dellie
Asmamaw Atnafu

Response to Reviewers:

Response to reviewers'
To: PLOS ONE Journal Editorial Office
Manuscript title: "Micronutrient intake status and associated factors among children aged 6-23 months in the emerging regions of Ethiopia: a multilevel analysis of the 2016 Ethiopian demographic and health survey" [Manuscript ID: PONE-D-20-13718].

Powered by Editorial Manager® and ProduXion Manager® from Aries Systems Corporation

Subject: Submission of a revised manuscript for publication
Dear Editor,
Greetings!
We appreciate and acknowledge the academic editor and reviewers for investing their time and energy to review and make comments on our manuscript once again. It is with great pleasure to receive the invaluable and constructive comments for our manuscript.
We accepted and tried to incorporate all of the comments provided. Moreover, the manuscript has been revised by English language expert and grammar and spellings have been improved throughout the manuscript. Thus, the comments are attached here below with their point-by-point responses. In addition, the detailed changes made are highlighted in the “revised manuscript with track changes” to easily identify the changes/improvements and also the clean copy of the revised manuscript is prepared. Finally, we kindly request you to review our revised manuscript.

RESPONSE TO EDITOR’S COMMENTS
Academic editor (Mary Hamer Hodges, MBBS MRCP DSc)
#1. I should double check that you are entitled to provide the dataset as supporting information file by contacting Measure DHS.
Authors’ response: Dear editor, thank you for your important comment. We have double checked the authorization letter, and unfortunately, we were prohibited to share the data set, “The data must not be passed on to other researchers without the written consent of DHS”. So, we would like to say sorry for the previous data set attachment as a supplementary information and we have removed the data set from the manuscript tracking system. Finally, we kindly request the journal office to remove the data set from the public repository if it’s deposited.

Response to Reviewers
Reviewer #1 (Anni-Maria Pulkki-Brannstrom):
1. I am surprised that a data file is provided as supporting information because as the authors correctly report, the data can be very easily accessed through contacting Measure DHS. I would recommend the authors double check that they are entitled to provide the dataset as supporting information file.
Authors’ response: Dear reviewer, we are very much thankful for your critical insights. We have checked the authorization letter; unfortunately, we were prohibited to share the data set, “The data must not be passed on to other researchers without the written consent of DHS”. So, we have removed the data from the supporting information and we hope you will definitely understand for the mistaken done in our previous submission regarding to the data file. Finally, we kindly request the journal editorial office to remove the data set from the supplementary files.

2. References to “received the recommended micronutrients” can be misunderstood to mean adequate intake. Please consistently refer to “received at least one of the recommended micronutrients” to ensure no reader is misled about your outcome variable. For example: in the results section of the Abstract, in the column heading in Table 6, and the first sentence of the discussion.
Authors’ response: Dear reviewer, thank you for your comment. We have amended the term of outcome variable “received the recommended micronutrients” in to “received at least one of the recommended micronutrients” in the results section of the Abstract, in the column heading in Table 6, and the first sentence of the discussion. Kindly see the clean version of the revised manuscript on page 2 lines 24-25, Table 6 column heading on page 20 and in the discussion page 23 lines 322-323.

3. Regarding the exclusion criteria, please rephrase “index to birth history” in Figure 1 and on row 120, because the meaning is difficult to understand.
Authors’ response: Dear reviewer, thank you so much for your comments. We have addressed the issue as per the comments, kindly see the clean version of the revised manuscript on page 7, lines 119-120.

Reviewer #2:
1. In the results section you stated children born of mothers who had ANC visits for

	their recent pregnancy were 1.95 times more likely to receive micronutrients. This may be related to knowledge gained from health talks during ANC visits. It would be prudent to proffer reasons for these differences.<br>Authors' response: Dear reviewer, thank you very much for your comment. We have mentioned that information and knowledge gained from health talks during ANC visits could be the possible explanations of micronutrient intake status difference. Kindly see the clean version of the revised manuscript on page 25, lines 373-376.<br><br>2. Also proffer reasons in the discussion section for why more mother tend to do complementary feeding for children ages 13-23 compared to 6-12months.<br>Authors' response: Dear reviewer, thank you for your comments; we have included the possible explanation. We tried to discuss the difference in feeding practice by stating the contributor factors of low feeding among children 6-12 months. Please see the clean version of the revised manuscript on page 24, lines 363-366.<br><br>3. I think it would be worth knowing why more mother preferred to give birth at compared to health facility. Is this because of distance to health facility, costs or they trust the traditional birth attendance more than they do health workers?<br>Authors' response: Dear reviewer, thank you very much for your insight. A plenty of studies identified that distance to health facility, mothers' low awareness about institutional delivery and others cultural and social factors were the high contributor for having a significant number of home delivery in Ethiopia. These factors were contributed not only for low institutional delivery services utilization and or coverage, but also for other maternal and neonatal health services uptake like postnatal services.
**Additional Information:**	
**Question**	**Response**
**Financial Disclosure**<br><br>Enter a financial disclosure statement that describes the sources of funding for the work included in this submission. Review the submission guidelines for detailed requirements. View published research articles from *PLOS ONE* for specific examples.<br><br>This statement is required for submission and **will appear in the published article** if the submission is accepted. Please make sure it is accurate.	The author(s) received no specific funding for this work.

Powered by Editorial Manager® and ProduXion Manager® from Aries Systems Corporation

**Unfunded studies**<br>Enter: *The author(s) received no specific funding for this work.*<br><br>**Funded studies**<br>Enter a statement with the following details:<br>• Initials of the authors who received each award<br>• Grant numbers awarded to each author<br>• The full name of each funder<br>• URL of each funder website<br>• Did the sponsors or funders play any role in the study design, data collection and analysis, decision to publish, or preparation of the manuscript?<br>• **NO** - Include this sentence at the end of your statement: *The funders had no role in study design, data collection and analysis, decision to publish, or preparation of the manuscript.*<br>• **YES** - Specify the role(s) played.<br><br>* typeset	
**Competing Interests**<br><br>Use the instructions below to enter a competing interest statement for this submission. On behalf of all authors, disclose any competing interests that could be perceived to bias this work—acknowledging all financial support and any other relevant financial or non-financial competing interests.<br><br>This statement **will appear in the published article** if the submission is accepted. Please make sure it is accurate. View published research articles from *PLOS ONE* for specific examples.	The authors have declared that no competing interests exist.

Powered by Editorial Manager® and ProduXion Manager® from Aries Systems Corporation

**NO authors have competing interests**<br><br>Enter: *The authors have declared that no competing interests exist.*<br><br>**Authors with competing interests**<br><br>Enter competing interest details beginning with this statement:<br><br>*I have read the journal's policy and the authors of this manuscript have the following competing interests: [insert competing interests here]*<br><br>* typeset	
**Ethics Statement**<br><br>Enter an ethics statement for this submission. This statement is required if the study involved:<br><br>• Human participants<br>• Human specimens or tissue<br>• Vertebrate animals or cephalopods<br>• Vertebrate embryos or tissues<br>• Field research<br><br>Write "N/A" if the submission does not require an ethics statement.<br><br>General guidance is provided below. Consult the submission guidelines for detailed instructions. **Make sure that all information entered here is included in the Methods section of the manuscript.**	The ethical approval and permission to access the data were obtained from the MEASURE DHS (available from https://www.dhsprogram.com/Data/ and accessed on April 06, 2020) after a brief study concept was submitted.

**Format for specific study types**<br><br>**Human Subject Research (involving human participants and/or tissue)**<br>• Give the name of the institutional review board or ethics committee that approved the study<br>• Include the approval number and/or a statement indicating approval of this research<br>• Indicate the form of consent obtained (written/oral) or the reason that consent was not obtained (e.g. the data were analyzed anonymously)<br><br>**Animal Research (involving vertebrate animals, embryos or tissues)**<br>• Provide the name of the Institutional Animal Care and Use Committee (IACUC) or other relevant ethics board that reviewed the study protocol, and indicate whether they approved this research or granted a formal waiver of ethical approval<br>• Include an approval number if one was obtained<br>• If the study involved *non-human primates*, add *additional details* about animal welfare and steps taken to ameliorate suffering<br>• If anesthesia, euthanasia, or any kind of animal sacrifice is part of the study, include briefly which substances and/or methods were applied<br><br>**Field Research**<br><br>Include the following details if this study involves the collection of plant, animal, or other materials from a natural setting:<br>• Field permit number<br>• Name of the institution or relevant body that granted permission	
**Data Availability**<br><br>Authors are required to make all data underlying the findings described fully available, without restriction, and from the time of publication. PLOS allows rare exceptions to address legal and ethical concerns. See the PLOS Data Policy and FAQ for detailed information.	No - some restrictions will apply

Powered by Editorial Manager® and ProduXion Manager® from Aries Systems Corporation

A Data Availability Statement describing where the data can be found is required at submission. Your answers to this question constitute the Data Availability Statement and **will be published in the article**, if accepted.<br><br>**Important:** Stating 'data available on request from the author' is not sufficient. If your data are only available upon request, select 'No' for the first question and explain your exceptional situation in the text box.<br><br>Do the authors confirm that all data underlying the findings described in their manuscript are fully available without restriction?	
**Describe where the data may be found in full sentences. If you are copying our sample text, replace any instances of XXX with the appropriate details.**<br><br>• If the data are **held or will be held in a public repository**, include URLs, accession numbers or DOIs. If this information will only be available after acceptance, indicate this by ticking the box below. For example: *All XXX files are available from the XXX database (accession number(s) XXX, XXX.).*<br>• If the data are all contained **within the manuscript and/or Supporting Information files**, enter the following: *All relevant data are within the manuscript and its Supporting Information files.*<br>• If neither of these applies but you are able to provide **details of access elsewhere**, with or without limitations, please do so. For example:<br><br>*Data cannot be shared publicly because of [XXX]. Data are available from the XXX Institutional Data Access / Ethics Committee (contact via XXX) for researchers who meet the criteria for access to confidential data.*<br><br>*The data underlying the results presented in the study are available from (include the name of the third party*	All relevant data are within the manuscript. Furthermore, data can be accessed from the MEASURE DHS (available from https://www.dhsprogram.com/Data/) after submitting a clear concept.

Powered by Editorial Manager® and ProduXion Manager® from Aries Systems Corporation

and contact information or URL).<br>• This text is appropriate if the data are owned by a third party and authors do not have permission to share the data.<br><br>* typeset	
Additional data availability information:	

Powered by Editorial Manager® and ProduXion Manager® from Aries Systems Corporation

Manuscript Click here to access/download;Manuscript;Clean version of the revised manuscript.docx

Micronutrient intake status and associated factors among children aged 6-23 months in the emerging regions of Ethiopia: a multilevel analysis of the 2016 Ethiopian demographic and health survey

Tsegaye Gebremedhin*, Andualem Yalew Aschalew, Chalie Tadie, Endalkachew Dellie, and Asmamaw Atnafu

Department of Health Systems and Policy, Institute of Public Health, College of Medicine and Health Sciences, University of Gondar, Gondar, Ethiopia

***Corresponding author**

Email: tsegishg27@gmail.com

Abstract

Background: Micronutrient deficiency is recognized as a major public health problem in developing countries, including Ethiopia. The scarcity of micronutrients, particularly in pastoral communities of Ethiopia, might be severe due to poor health care access, drought and poverty. However, empirical evidence is scarce in Ethiopia. Therefore, this study aimed to assess the micronutrient intake status of children aged 6 to 23 months in the emerging regions of Ethiopia.

Methods: Data from the 2016 Ethiopia Demographic and Health Survey (EDHS) were used. A two-stage stratified sampling technique was employed to identify 1009 children aged 6 to 23 months. A multilevel mixed-effect logistic regression analysis was used to identify individual and community-level factors associated with micronutrient intake status. In the final model, variables with a p-value of < 0.05 and adjusted odds ratio (AOR) with 95% confidence interval (CI) were used to identify factors statistically associated with micronutrient intake status.

Results: Overall, 62.7% (95% CI: 59.7-65.7) of children aged 6 to 23 months had received at least one of the recommended micronutrients. Antenatal care (ANC) visit (AOR: 1.95, 95% CI: 1.37-2.77), work in the agriculture (AOR: 2.22, 95% CI: 1.30-3.80) and children aged 13 to 23 months (AOR: 1.72, 95% CI: 1.25-2.36) were the individual-level factors, whereas rural residence (AOR: 0.37, 95% CI: 0.14-0.88), reside in Benishangul (AOR: 2.25, 95% CI: 1.29-4.93) and Gambella regions (AOR: 1.87, 95% CI: 1.03-3.38) were the community-level factors associated with micronutrient intake status.

Conclusions: The micronutrient intake status in the study area was low compared to the national recommendation. Promoting vitamin A and iron-rich foods and micronutrient powders at individual and community-level and strengthen supplementations and deworming alongside

the community-based maternal and child health services would improve the micronutrient intake among children.

Introduction

Micronutrient deficiency is recognized as a global public health problem among children, and it is worse in low- and middle-income countries, particularly in Ethiopia [1-3].

The commonest micronutrients needed for life are iron, zinc, calcium, iodine, and vitamins, which can be found from foods rich in vitamins and minerals or in the form of supplements, such as vitamin A supplement [4-6]. Although micronutrients are only needed in small quantities, their absence from children's diet negatively affects the survival and development of children. Micronutrient deficiency may lead to devastating consequences, like stunting, wasting, weakened immunity, and delay in cognitive development [7-11]. Notably, micronutrient is very critical during the first two years of a child's life; as adequate nutrition during this period promotes healthy growth and development. Yet, micronutrient deficiency received less attention than the apparent starvation of people who are unable to get enough food to survive [12, 13].

Furthermore, most infants and young children around the world are not getting the healthy diets that they need for life and grow well. According to the United Nations Children's Fund (UNICEF) 2019 report, there were around 340 million children worldwide who suffered from hidden hunger caused by deficiencies of vitamins and minerals [14]. Globally, a million child deaths per year due to vitamin A and zinc deficiencies were reported in 2016 [15-17]. Nevertheless, the magnitude of the problem is much higher in low- and middle-income countries; only 29% of children aged 6–23 months were fed the minimum diversified diet needed for growth and development [18].

Similarly, the deficiency of crucial vitamins and mineral are among the significant public health problems in Ethiopia. These deficiencies are a result of diets with limited diversity, minimal bioavailability, frequent meal skipping, limited access to micronutrient-rich and

fortified foods, and low vegetable and fruit intake [15, 19, 20]. According to the Ethiopian Demographic and Health Survey (EDHS) 2016 report, only 14% of the children aged 6–23 months received the minimum dietary diversity [21]. Moreover, studies in different parts of the country reported only 13 to 43.2% of children aged 6-23 months consumed a diversified diet [22-26]. The Ethiopian national nutritional supplementation survey (2016) indicated that vitamin A supplementation coverage in children was 63%, which is lower than the national target (more than 90%) and vitamin A deficiency was significant (14%) [27].

Micronutrient intake is associated with various factors at individual and community levels, including mothers' sociodemographic and child characteristics, dietary habits, community-level lifestyle and place of residence [28-30]. On top of the above factors, the use of maternal healthcare services, such as antenatal care (ANC), institutional delivery and postnatal care (PNC) visits, are also associated with the micronutrient intake status of children [31, 32].

Although there is documented evidence of insufficient micronutrient intake for agrarian communities and urban dwellers in Ethiopia [22, 24, 25, 33], there is little evidence on micronutrient intake among children aged 6-23 months in emerging regions of Ethiopia where pastoralist communities, with poor cultivation of fruits and vegetables, are mainly reside [34]. Additionally, these regions have been identified as the hotspot in the country with high food insecurity, high child malnutrition rates, and the recurrent onset of droughts [35, 36].

Besides, these areas have limited access to health facilities, poor infrastructure and inaccessible health services that could worsen the micronutrient deficiency [37, 38]. However, studies that show the individual and community-level factors of micronutrient intake among children are rare. The objective of this study is, therefore, to assess the micronutrient intake status and related factors among children aged 6-23 months in emerging regions of Ethiopia by using the 2016 EDHS data. Moreover, the finding could give important insights to develop contextual strategies for the mitigation of the problem.

Materials and Methods

Study settings and data source

The study used the EDHS 2016 data, a nationally representative household survey data collected every five years. It has been implemented by the Central Statistical Agency (CSA) [39] with the primary objective of providing up-to-date estimates of key demographic and health indicators. Administratively, Ethiopia is divided into nine and two geographical regions and administrative cities, respectively. Of the total regional states, Afar, Somali, Benishangul, and Gambela are the emerging regions of Ethiopia where pastoralists predominantly live.

Sampling procedures

The sampling frame for the 2016 EDHS used the 2007 Ethiopian population and housing census, which was conducted by the CSA of Ethiopia. The census used a complete list of 84,915 enumeration areas (EAs), which contains the location, type of residence, and the estimated number of residential households. Then the sample for the 2016 EDHS was stratified in two stages, and samples of EAs were selected independently from each stratum. The regions were stratified into urban and rural areas. At each of the lower administrative levels, implicit stratification and proportional allocation were achieved within each sampling stratum before sample selection at different levels.

In the first stage, 645 EAs were selected with probability proportional to the EA size, and each sampling stratum was selected from the given samples. The total residential households in the EA were the EA size, and a household listing operation was implemented. Then, the resulting lists of households were used as the sampling frame for the selection of households in the second stage. Some of the selected EAs were large. The selected large EAs with more than 200 households were segmented to minimize the task of household listing. Through the probability

proportional to the segment size, only a segment was selected for the survey, and the household listing was conducted for each selected segment.

A fixed number of 28 households from each cluster were selected with an equal probability in the second stage, a systematic selection from the newly created household listing. The survey interviewer interviewed only pre-selected households. No replacements or changes of the pre-selected households were allowed in the implementing stages to prevent bias. In this study, the 2016 EDHS childhood datasets of the four emerging regional states: Afar, Benishangul, Gambella and Somali, were used for analysis.

All women aged 15-49 years who are the usual members of the selected households were eligible for the female survey. Children aged 6–23 months were the source population and included 1009 mothers and their children aged 6-23 months were included in the analysis. Alive, last child and live with their mothers/caregivers were included, while those second and above child and live with other than their mothers/caregivers were excluded from the analysis (Fig 1). Additionally, for twins, a mother was asked for a child only. Potential individual and community level independent variables were also extracted and further analysis was done.

Fig 1. Sample study selection of children age 6-23 months in emerging regions, EDHS 2016

Measurements of variables

The dependent variable of the study was micronutrient intake status among children aged 6-23 months, which was determined from the reports of mothers regarding the routine micronutrient intake of their children. The intake status was measured based on the recommendations of daily micronutrient intake that meets the micronutrient requirements of almost all healthy individuals in age and sex-specific population groups [40-43]. The recommended micronutrient for children are foods rich in vitamin A within 24 hours, foods rich in iron within 24 hours, multiple micronutrient powder within seven days, iron supplements within seven days, vitamin A supplements within six months, or deworming medication within six months [41, 42, 44-46]. Accordingly, if the children received at least one of the minimum recommended micronutrients, we considered as "Yes"; if the children received none of the minima recommended micronutrients, it was considered as "No".

Foods rich in vitamin A were measured by the consumption of either of the seven food groups within the preceding 24 hours. These food groups were I. Eggs, ii. Meat (beef, pork, lamb, chicken), iii. pumpkin, carrots, and squash, iv. any dark green leafy vegetables, v. mangoes, papayas, and others with vitamin A fruits, vi. Liver, heart, and other organs and vii. Fish or shellfish.

Foods rich in iron were measured by the consumption of either of the four iron-rich food groups within the past 24 hours. These groups were i. eggs, ii. meat (beef, pork, lamb, chicken), iii. Liver, heart, and other organs, and iv. Fish or shellfish.

Multiple micronutrient powders were assessed by asking the mother whether their child had received micronutrient powders in the previous seven days. The micronutrients contained a range of vitamins and minerals (and nearly always include iron, per WHO recommendations) enclosed in a single-dose sachet added to foods; Sprinkles™ is a brand name used for micronutrient powders.

Iron supplementation was assessed by asking the mothers whether their child had iron supplementation defined as iron pills, sprinkles with iron, or iron syrup in the previous seven days.

Vitamin A supplementation and deworming medication have been provided for children aged 6-59 months by semi-annually as a national nutrition program. For this study, vitamin A supplementation and deworming were assessed for those 6 to 23 months of children whether they received for the last six months or not by reviewing the integrated child card, which consists of immunization and growth monitoring history.

Individual and community-level characteristics were the independent variables. The individual-level variables were individual sociodemographic and economic characteristics, obstetric history of the women, and child characteristics. At the same time, the community-level variables were residence, region, community-level poverty, community-level media exposure, the distance of the nearest health facility.

The wealth quintile was calculated as an index based on consumer goods such as television, bicycle, or car. Household characteristics were also considered in calculating the wealth index. These scores were derived using principal component analysis and ranked into poor, middle, and rich. The wealth quintiles are expressed in terms of quintiles of individuals in the population rather than quintiles of individuals at risk for anyone's health or population indicator.

Distance to the health facility was assessed by the question “distance to the nearest health facility is a problem?” and the responses were categorized as “big problem” or “not a problem” [47].

Community-level poverty was assessed using the asset index based on data from the entire country sample on separate scores prepared for rural and urban households, and combined to

produce a single asset index for all households as the community level and ranked into five (poorest, poorer, middle, richer, and richest).

Community media exposure was assessed as “yes” if they have access to all three media (newsletter, radio, and television) at least once a week, otherwise “no” if they did not have any media exposure.

Data processing and statistical analysis

The data were cleaned, re-coded and analysed using STATA (StataCorp, College Station, TX) version 14. Descriptive statistics were presented using tables and narration to describe the magnitude of micronutrient intake status by sociodemographic, maternal obstetric and child characteristics.

A multilevel analysis was conducted after checking the eligibility. The model eligibility was assessed by calculating the Intra-class Correlation Coefficient (ICC) and model with ICC greater than 10% is eligible for multilevel analysis. In this study, the ICC was 27.3%. Since the data were hierarchical (individuals were nested within communities), a two-level mixed-effects logistic regression model was fitted to estimate both the individual and community level variables (fixed and random) effect on micronutrient intake status, and the log of the probability of micronutrient intake was modelled using the formula as follows [48]:

$$log\ [\frac{\pi ij}{1-\pi ij}] = \beta_o + \beta_1 X_{ij} + \beta_2 Z_{ij} + U_j + e_{ij}$$

Where i is individual level unit and j is a community-level unit; X and Z refer to individual and community-level variables, respectively; πij is the probability of micronutrient intake for the ith child in the jth community; the β's are the fixed coefficients. Whereas, β_0 is the intercept-the effect on the probability of micronutrient intake in the absence of influence of predictors; and u_j showed the effect of the community (random effect) on micronutrient intake for the jth

community and e_{ij} showed random errors at the individual levels. By assuming each community had different intercept ($\beta_0 + U_j$) and fixed coefficient ($\beta_{1,2}$), the clustered data nature and the within and between community variations were taken into account.

Bivariable and multivariable analysis were computed. First, in the bivariable logistic regression analysis, a p-value of less than 0.2 was used to fitted three models (models for the individual level, community level, and both the individual and community level). Then, in the final model (fixed effect), a p-value less than 0.05 and adjusted odds ratio (AOR) with 95% confidence interval (CI) were used to estimate the association of individual and community level factors with micronutrient intake status.

The measures of variation (random-effects) were reported using ICC and proportional change in variance (PCV) to measure the variation between clusters. The ICC refers to the ratio of the between-cluster variance to the total variance, and it tells us the proportion of the total variance in the outcome variable that is accounted at the cluster level. The loglikelihood test was used to estimate the goodness of fit of the adjusted final model in comparison to the preceding models. A model with the smallest value of loglikelihood is better; accordingly, model three (a model for both the individual and community level variables) had the lowest value.

Ethical considerations

The ethical approval and permission to access the data were obtained from the MEASURE DHS (available from https://www.dhsprogram.com/Data/: accessed on April 06, 2020) after a brief study concept was submitted.

Results

Sociodemographic and economic characteristics of participants

A total of 1009 mothers/caregivers with children aged 6-23 months were included in the final analysis. The mean age of the mothers was 27.5 (SD ± 6.3) years, and 83.9% of them were

rural dwellers. The majority (72.4%) of the households were in the poor wealth status; the mean family size was 5.9 (SD± 2.3). Nearly one-third (34.3%) of the participants were from the Somali region and the majority (71.1%) were Muslim by religion. Of the participants, 95.2% were married; 59.3% with no education and 66.5% had no work. Similarly, 59.3 and 46.6% of their husbands/partners had no education and agricultural workers, respectively (Table 1).

Table 1. Sociodemographic and economic characteristics of study participants in emerging regions of Ethiopia, 2016 (n=1009).

Variables	Category	Frequency (n)	Percent (%)
Age of mothers/caregivers in years	15-24	341	33.8
	25-34	497	49.3
	>=35	171	16.9
Residence	Urban	163	16.1
	Rural	846	83.9
Region	Afar	254	25.2
	Somali	346	34.3
	Benishangul	224	22.2
	Gambela	185	18.3
Religion	Muslim	717	71.1
	Protestant	173	17.1
	Orthodox	85	8.4
	Others*	34	3.4
Sex of household head	Male	674	66.8
	Female	335	33.2
Household wealth status	Poor	730	72.4
	Middle	78	7.7
	Rich	201	19.9
Current marital status	Married	961	95.2
	Unmarried	48	4.8
Educational status of mothers/caregivers	No education	715	70.9
	Primary education	199	19.7
	Secondary education	67	6.6
	Higher	28	2.8
Educational status of husband's/partner's (n=961)	No education	570	59.3
	Primary education	197	20.5
	Secondary education	104	10.8
	Higher	90	9.4

Variables	Category	Frequency (n)	Percent (%)
Respondent's occupation	No work	671	66.5
	Professional worker	80	7.9
	Agricultural worker	189	18.7
	Others**	69	6.8
Husband’s/partner’s occupation (n=961)	No work	139	14.5
	Professional worker	175	18.2
	Agricultural worker	448	46.6
	Others***	199	20.7

*Catholic, traditional, Joba,

**Daily labor, merchant

***Daily labor, merchant

Obstetric history of participants

The majority (54.5%) of women were in the age group of 18 to 24 years when they gave their first birth. Of the total women, 45.2% of them were multipara, and 59.9% have two to five living children. Nearly 56% of the women had ANC visits for their recent birth, and out of them, 55.9% had first visit. Furthermore, only one-fourth of the women delivered at health facilities, and 7.2% of them had PNC checks within two months after delivery (Table 2).

Table 2. Obstetric characteristics of participants in the emerging regions of Ethiopia, 2016 (n=1009).

Variables	Category	Frequency (n)	Percent (%)
Age of the mother at first birth (in years)	<18	402	39.8
	18-24	550	54.5
	25+	57	5.7
Parity	Primipara	176	17.4
	Multipara	456	45.2
	Grand multipara	377	37.4
Number of living children	1	174	17.2
	2-5	604	59.9

Variables	Category	Frequency (n)	Percent (%)
	6+	231	22.9
ANC visit	Yes	569	56.4
	No	440	43.6
Desire for more children	Wants	798	79.1
	Undecided	43	4.3
	Wants no more	168	16.6
Place of delivery	Home	745	73.8
	Health facility	264	26.2
PNC check within two months	Yes	73	7.2
	No	936	92.8
Timing after postnatal delivery check took place (n=73)	Within 24hrs	7	9.6
	1-7th day	41	56.2
	After the 7th day	25	34.2
Current pregnancy status	Pregnant	90	8.9
	Non-pregnant	919	91.1

ANC: Antenatal care, PNC: Postnatal care

Child characteristics and common childhood illness

Of the total children, 54.1% were male and 54.2% were in the age group of 13 to 23 months. Moreover, 42.0% of the children had average birth weight;55.8% of the children were found between the second and fifth-order of birth. Approximately 96% of the children were measured their current weight, and of those, 79.0% were weighted 7-11kgs. Additionally, 15.9, 13.6, and 15.8% of the children had diarrhea, cough, and fever within the last two weeks, respectively (Table 3).

Table 3. Child characteristics and common childhood illness among children aged 6 to 23 months in the emerging regions of Ethiopia, 2016 (n=1009).

Variables	Category	Frequency (n)	Percent (%)
Sex of the child	Male	546	54.1
	Female	463	45.9

The current age of the child in months	6-12	462	45.8
	13-23	547	54.2
Size of a child at birth	Large	269	26.7
	Average	424	42.0
	Small	316	31.3
Birth order	1	176	17.4
	2-5	563	55.8
	6+	270	26.8
Preceding birth interval in months (n=833)	<=24	241	28.9
	25-36	282	33.8
	37-48	163	19.6
	>=49	147	17.7
Current weight of the child measured	Yes	964	95.6
	No	45	4.4
Current weight of child in kg (n=964)	<7	145	15.0
	7-11	762	79.0
	11+	57	6.0
Had diarrhea	Yes	160	15.9
	No	849	84.1
Had cough	Yes	137	13.6
	No	872	86.4
Had fever	Yes	159	15.8
	No	850	84.2

Community-level poverty, media exposure and access to a health facility

The analysis showed that 63.1, 16.1, 9.3, 7.3 and 4.2% of the communities are in the poorest, poorer, middle, richer and richest status, respectively. Only five percent of the community has media exposure. Additionally, 42.0% of the respondents mentioned that access to a health facility is not a problem.

Micronutrient intake status among children aged 6-23 months

Overall, 62.7% (95% CI: 59.7-65.7) of the children received the minimum recommended micronutrients. Of those who took the recommended micronutrient, only 12.9% had received three and above micronutrient types.

Moreover, 27.8% (95% CI: 25.0-30.5) of the children consumed foods rich in vitamin A at any time in 24 hours preceding the interview, and 15.6% (95% CI: 13.3-17.8) of them have

consumed foods rich in iron at any time in 24 hours preceding the interview. Additionally, 7.5% (95% CI: 5.9-9.2) of the children received multiple micronutrient powder in the seven days preceding the interview, and 6.0% (95% CI: 4.5-7.4) of them received iron supplements in the seven days preceding the interview. Around 47% (95% CI: 44.1-50.3) of the children received vitamin A supplements in the six months preceding the interview. Besides, 8.4% (95% CI: 5.5-8.7) of the children aged 12 to 23 months of were received deworming medication in the six months preceding the interview (Table 4).

Table 4. Micronutrient intake status among children aged 6-23 months in the emerging regions of Ethiopia, 2016 (n=1009).

Food groups and supplementations	Contains/measurements	Received n (%)	No received n (%)
Consumed foods rich in vitamin A within 24 hours	Eggs	85 (8.4)	924 (91.6)
	Meat (beef, pork, lamb, chicken, etc)	52 (5.2)	957 (94.8)
	Pumpkin, carrots, and squash	111 (11.0)	898 (89.0)
	Any dark green leafy vegetables	91 (9.0)	918 (91.0)
	Mangoes, papayas, and others with vitamin A fruits	133 (13.2)	876 (86.8)
	Liver, heart, and other organs	32 (3.2)	977 (96.8)
	Fish or shellfish.	45 (4.5)	964 (95.5)
**Overall vitamin A rich foods consumptions**		**280 (27.7)**	**729 (72.3)**
Consumed foods rich in iron at any time in 24 hours	Eggs	85 (8.4)	924 (91.6)
	Meat (beef, pork, lamb, chicken)	52 (5.2)	957 (94.8)
	Liver, heart, and other organs	32 (3.2)	977 (96.8)
	Fish or shellfish.	45 (4.5)	964 (95.5)
**Overall iron rich food consumption**		**157 (15.6)**	**852 (84.4)**

Multiple micronutrient powder within seven days	76 (7.5)	933 (92.5)
Iron supplements within seven days	60 (6.0)	949 (94.0)
Vitamin A supplements within six months	476 (47.2)	533 (52.8)
Deworming medication in the six months (n=547)	46 (8.4)	501 (91.6)
**Overall, received at least the minimum recommended micronutrient**	**633 (62.7)**	**376 (37.3)**

Random effects (measures of variation)

There was a significant variation in the intake of micronutrients among children aged 6 to 23 months across the communities (clusters). The intra-cluster correlation coefficient (ICC) in the null model (model 0) for micronutrient intake was 0.273. In other words, 27.3% of the variation in micronutrient intake among children aged 6 to 23 months is due to the differences between regions/clusters (between-cluster variation) (Table 5).

Table 5. Results from a random intercept model (a measure of variation) for micronutrient intake among children aged 6 to 23 months at cluster level by multilevel logistic regression analysis, EDHS 2016.

Measure of variations	Model 0 (null model)	Model 1	Model 2	Model 3 (full model)
Variance	3.35	1.49	1.61	1.43
Explained variation (PCV) (%)	Ref.	55	52	57
ICC (%)	27.3	62.2	73.3	79.9
Model fitness				
Deviance (-2*log likelihood)	1271.9	1154.7	1195.0	1135.4
AIC	1275.9	1200.4	1214.5	1193.8

AIC: Akaike's Information Criterion

ICC: Intra-class Correlation Coefficient

PCV: Proportional Change in Variance

Model 0: without independent variables (null model)

Model 1: only individual-level variables

Model 2: only community-level variables

Model 3: individual and community-level variables (full model)

Individual and community-level factors of micronutrient intake status (fixed effects)

In the bivariable analysis, mothers educational status and occupation, sex of household head, household wealth status, age at first birth, ANC and PNC visits, place of delivery, desire for more child, age of the child, currently breastfeeding, history of diarrhea and cough within the last two weeks and current pregnancy status was the individual level candidate variables. Whereas residence, region, community-level poverty and community level media exposure were the community level candidate variables.

In the final model (model 3), after adjusting for individual and community level factors, women's occupational status, the current age of the child, antenatal visits for current pregnancy, residence and region were significantly associated with the micronutrient intake status among children aged 6 to 23 months.

Accordingly, children whose mothers/caregivers with an agricultural occupation were 2.22 times more likely to receive the recommended micronutrient compared to those children whose mothers/caregivers with no work (AOR: 2.22, 95% CI: 1.30-3.80). Children born from mothers who had ANC visits for their recent pregnancy were 1.95 times more likely to receive the recommended micronutrient compared to those who had not ANC visits (AOR: 1.95, 95% CI:1.37-2.77). Those children aged 13 to 23 months were 1.72 times more likely to receive the recommended micronutrient compared to those aged 6 to 12 months (AOR: 1.72, 95% CI: 1.25-2.36). Those children who reside in the rural communities were 63% less likely to receive the recommended micronutrient compared to their counterparts (AOR: 0.37, 95% CI: 0.14-

0.88). Children who live in the Benishangul and Gambella region were 2.52 (AOR: 2.52, 95% CI: 1.29-4.93) and 1.87 (AOR: 1.87, 95% CI: 1.03-3.38) times more likely to take the recommended micronutrient compared to those children who live in the Afar region, respectively (Table 6).

Table 6. Multilevel logistic regression analysis of factors associated with micronutrient intake status among children aged 6-23 months in the emerging regions of Ethiopia, EDHS 2016 (n=1009).

Variables	Received at least one of the recommended micronutrients		COR (95%CI)	Model 0 (ICC: 27.3%)	Model 1 AOR (95% CI)	Model 2 AOR (95%CI)	Model 3 AOR (95%CI)
	Yes n (%)	No n (%)					
Individual-level characteristics							
Mothers educational status							
No education	409 (57.2)	306 (42.8)	1		1		1
Primary	150 (75.4)	49 (24.6)	1.89 (1.24-2.88)		1.44 (0.94-2.22)		1.19 (0.76-1.87)
Secondary	52 (77.6)	15 (22.4)	2.38 (1.19-4.76)		1.52 (0.75-3.10)		1.20 (0.57-2.54)
Higher	22 (78.6)	6 (21.4)	2.47 (0.84-7.20)		1.17 (0.38-3.56)		0.83 (0.25-2.75)
Household head							
Male	435 (64.5)	239 (35.5)	1		1		1
Female	198 (59.1)	137 (40.9)	0.77 (0.55-1.07)		0.81 (0.58-1.12)		0.86 (0.61-1.19)
Mothers' occupation							
No work	371 (55.3)	300 (44.7)	1		1		1
Professional	57 (71.3)	23 (28.7)	1.78 (0.98-3.21)		1.38 (0.75-2.54)		1.38 (0.75-2.55)
Agricultural	154 (81.5)	35 (18.5)	3.34 (2.06-5.43)		3.04 (1.89-4.88)		2.22 (1.30-3.80) *
Others	51 (73.9)	18 (26.1)	2.03 (1.07-3.86)		1.51 (0.78-2.93)		1.34 (0.68-2.62)
Wealth status							
Poor	418 (57.3)	312 (42.7)	1		1		1
Middle	61 (78.2)	17 (21.8)	2.33 (1.23-4.40)		1.43 (0.75-2.74)		1.17 (0.46-3.00)
Rich	154 (76.6)	47 (23.4)	2.12 (1.34-3.59)		1.40 (0.86-2.26)		0.54 (0.20-1.47)
Age at first birth in years							
Less than 18	248 (61.7)	154 (38.3)	1		1		1
18-24	347 (63.1)	203 (36.9)	1.23 (0.89-1.69)		1.18 (0.86-1.62)		1.18 (0.86-1.63)
25+	38 (66.7)	19 (33.3)	1.54 (0.76-3.13)		1.43 (0.72-2.87)		1.40 (0.69-2.86)

ANC visit							
No	214 (48.6)	226 (51.4)	1		1		1
Yes	419 (73.6)	150 (26.3)	2.78 (2.01-3.84)		2.03 (1.43-2.86)		1.95 (1.37-2.77) *
Place of delivery							
Home	429 (57.6)	316 (42.4)	1		1		1
Health facility	204 (77.3)	60 (22.7)	2.39 (1.61-3.54)		1.31 (0.85-2.02)		1.20 (0.77-1.89)
PNC visit							
Yes	62 (84.9)	11 (15.1)	1		1		1
No	571 (61.0)	365 (39.0)	0.30 (0.14-0.64)		0.54 (0.25-1.13)		0.54 (0.25-1.17)
Desire more child							
Wants	478 (59.9)	320 (40.1)	1		1		1
Undecided	27 (62.8)	16 (37.2)	1.24 (0.59-2.59)		1.05 (0.50-2.19)		1.15 (0.55-2.39)
Wants no more	128 (76.2)	40 (23.8)	1.94 (1.24-3.05)		1.87 (1.19-2.94)		1.54 (0.96-2.46)
Child currently breastfeed							
No	111 (56.1)	87 (43.9)	1		1		1
Yes	522 (64.4)	289 (35.6)	1.36 (0.93-1.98)		1.17 (0.76-1.82)		1.15 (0.74-1.80)
Currently pregnant							
No	585 (63.7)	334 (36.3)	1		1		1
Yes	48 (53.3)	42 (46.7)	0.62 (0.36-1.05)		0.73 (0.40-1.32)		0.79 (0.43-1.44)
Age of child in months							
6-12	259 (56.1)	203 (43.9)	1		1		1
13-23	374 (68.4)	173 (31.6)	1.74 (1.28-2.35)		1.79 (1.31-2.45)		1.72 (1.25-2.36) *
Diarrhoea in the last two weeks							
No	522 (61.5)	327 (38.5)	1		1		1
Yes	111 (69.4)	49 (30.6)	1.53 (1.00-2.35)		1.27 (0.81-1.99)		1.28 (0.82-2.00)
Cough in the last two weeks							
No	558 (64.0)	314 (36.0)	1		1		1
Yes	75 (54.7)	62 (42.3)	0.73 (0.47-1.12)		0.59 (0.38-0.92)		0.65 (0.41-1.02)
Community-level characteristics							
Residence							
Urban	126 (77.3)	37 (22.7)	1			1	1

Rural	507 (60.0)	339 (40.0)	0.42 (0.24-0.75)			0.42 (0.24-0.72)	0.35 (0.14-0.88) *
Region							
Afar	127 (50.0)	127 (50.0)	1			1	1
Somali	182 (52.6)	164 (47.4)	1.11 (0.69-1.81)			1.02 (0.65-1.59)	1.13 (0.71-1.76)
Benishangul	187 (83.4)	37 (16.6)	6.44 (3.54-11.7)			5.27 (2.87-9.66)	2.52 (1.29-4.93) *
Gambella	137 (74.0)	48 (26.0)	3.81 (2.12-6.87)			2.87 (1.63-5.04)	1.87 (1.03-3.38) *
Community-level poverty							
Poorest	350 (55.0)	287 (45.0)	1			1	1
Poorer	120 (74.0)	42 (26.0)	1.89 (1.19-3.00)			1.20 (0.74-1.94)	1.07 (0.64-1.77)
Middle	67 (71.3)	27 (28.7)	1.88 (1.08-3.26)			1.15 (0.64-2.04)	0.78 (0.35-1.73)
Richer	62 (83.8)	12 (16.2)	3.65 (1.77-7.52)			2.04 (0.98-4.26)	2.34 (0.76-7.13)
Richest	34 (81.0)	8 (19.0)	3.43 (1.42-8.31)			1.95 (0.79-4.80)	2.59 (0.67-9.93)
Community-level media exposure							
No	593 (61.8)	366 (38.2)	1			1	1
Yes	40 (80.0)	10 (20.0)	1.89 (0.83-4.30)			1.03 (0.44-2.36)	1.07 (0.44-2.61)

*****Statistically significant at p-value <0.05 at model 3

ANC: Antenatal Care,

AOR: Adjusted Odds Ratio,

COR: Crude Odds Ratio,

ICC: Intra-class Correlation Coefficient,

PNC: Postnatal Care

Discussion

The study showed that at least one of the recommended micronutrient intake among children aged 6-23 months in the emerging regions of Ethiopia was found to be 62.7%. The individual-level factors such as the age of the child, mother's occupation, and current ANC follow up have been significantly associated with the intake of micronutrients. From the community-level factors, residence and region were significantly associated with micronutrient inta[illegible]

Micronutrient is available in foods and can also be provided through a direct supplement. In this study, we assessed the level of vitamin A and iron intake. The result showed that around 28 and 15.6% of children consume foods rich in vitamin A and iron, respectively. From EDHS 2016 report, consumption of foods rich in vitamin A and iron were 38 and 22%, correspondingly, and lowest intake was observed in Afar [49], which is comparable with the current finding. Besides, almost half of the children (47.18%) get vitamin A supplements and as few as 6% of them get iron supplements. The previous EDHS (2011) finding showed that vitamin A supplement, in the four regions, was 43.17%, which is lower than the current result [36]. This might be due to the emphasis given by the government to achieve the first phase of the nutritional program.

Moreover, the national micronutrient survey (2016) revealed that the national coverage of vitamin supplements was 63% [50], which is higher than the current finding. However, the report showed that sub-clinical vitamin A deficiency was 14%. Vitamin A supplement in Nigeria was 45% which is comparable with the current finding [51]. However, our study differs from the finding in India (30.4%) [52].

The finding showed that at least one of the recommended micronutrient intake status was 62.7%, which is lower than that of the national target of over 90% [53]. Besides, there was a considerable difference between the regions. The National Nutritional Program end-line survey

report was also comparable with the current findings [54]. Benishangul and Gambella regions where agriculture, a source of legume and cereal plants, is very common, had a higher intake. However, regions of Afar and Somalia are inhabited by pastoralist people with a scattered settlement where fresh fruit and vegetables are not readily available. Besides, healthcare facilities may also be inaccessible and under-staffed. A study from the pastoral community showed that the majority source of food was dairy products [55].

This study identified that micronutrient intake among children whose mothers worked in agriculture was higher compared with children whose mothers did not have work. Mothers who work in agriculture might have better access to diversified agricultural products and animal products that are rich sources for vitamins and minerals. Moreover, participating in work may expose mothers with peers and friends that can serve as a source of information related to micronutrient intake and benefits. This study also showed that agrarian dominants were more likely to consume diversified food, which can be used as a proxy for adequate micronutrient density of foods [56]. Previous studies are also consistent with the current finding [36, 57].

The odds of micronutrient intake for children age 13-23 were higher compared to children age between 6-12 months. The possible explanation could be that the age groups have better nutritional diversity. In the EDHS 2016, for example, children over 12 months old were more likely to obtain diversified food, which implies that they can got more micronutrients [56, 58]. On the other way, the late introduction of complementary feeding might have resulted in consuming a limited variety of food such as only milk or cereal products. Moreover, mothers' perception of low ability of children intestine and traditional beliefs might contribute to low consumption of diversified food in those children (6-12 months). Also, even if vitamin A supplement is effective for 6-11 months, especially when used with the vaccine against measles, vitamin A supplementation can be reduced by dropping out of this age group and

certain children (6-12 months) were not eligible to receive vitamin A supplement during the survey. Studies from Nigeria are also comparable to the current finding [51, 57].

In this study, higher odds of micronutrient intake were observed among children whose mother had ANC follow-ups compared to those children whose mother did not have ANC follow-ups. This finding was in line with these of previous studies conducted in Ethiopia [36]. The possible explanation might be mothers who had ANC follow-up may have a chance of getting information, education, knowledge and also counselling services on foods rich in micronutrients and its availability from health professionals' talks. Furthermore, they might able to learn about the value of iron intake that supplements during their ANC follow-up. Moreover, a systematic review by Temesgen et al. suggests that children whose mothers have ANC follow-up have a higher probability than their counterparts to eat diversified food [59].

The odds of micronutrient intake among children who reside in rural communities were lower compared to their counterparts. This is supported by a systematic study in Ethiopia, which reported that urban residents had higher odds of micronutrient intake than rural residents [59]. However, few studies' findings [51, 52, 60] contradict with the current study. The potential explanation might be food fortification and supplementation focused more on rural than urban through community-based maternal and child health outreach programs.

Our finding shows that micronutrient intake among children who live in Benishangul and Gambella regions was higher compared to those who live in the Afar region. This can be explained by the fact that, compared to the two regions, the economic activities of the Afar and Somalia regions are mostly dominated by cattle breeding and pastoral lifestyles, and agriculture is common in Benishangul and Gambella. Besides, since the latter two regions have dense forests and water reservoirs, they could get wild fruit and fish, which are a good source of micronutrients. Previous studies showed that in the pastoral community, vitamin A rich foods were scarce, and meat and egg consumption were low [55]. Natural forest and semi-natural

forests were positively associated with a larger number of nutritionally important food groups [61]. A study from the recent EDHS (2016) showed that children of the agrarian community were more likely to consume diversified food than that of the pastoral community.

Strength and limitations of the study

The main strengths of the study are its representativeness, large sample size, and the availability of individual and community-level factors that demonstrate the actual micronutrient intake status of children in the emerging regions. Besides, this study used a multilevel-modelling technique to identify a more valid result that takes the hierarchical nature of the survey data into account. Furthermore, the DHS surveys have similar designs with similar variables in a different setting; the result may, therefore, be applied to other similar settings.

The study tried to assess the intake status of micronutrient using the national data and discuss this with other cross-sectional studies; focus on nutritional studies that can under or overestimates our findings. Moreover, the study used the national micronutrient recommendation for the assessment of the outcome variable which might not significantly measure the adequacy of intake. Furthermore, the mothers might have experienced recall bias, particularly regarding vitamin A supplementation and deworming for their child in the last six months prior to the survey, for instance. Compared to other studies, however, our study assessed later events that preceded the study by only six months which might decrease the recall bias.

Conclusions

In conclusion, the overall intake of micronutrients in this study was below the national recommendation. Besides, there was a lower intake of micronutrients in the two pastorally dominated regions (Afar and Somalia). Individual-level characteristics such as mothers'

occupation and age of a child and community-level characteristics ANC for index child, residence, and region were significantly associated with the micronutrient intake status. Therefore, for the improvement of micronutrient intake, the regional and zonal health departments and the health facilities needs to cooperate with state governments across the regions, continuously increase the amount of vitamin A and the iron supplementation, encourage the use of Vitamin A and iron-rich foods, and reinforce community-based outreach programs for maternal health and child health programs.

List of Abbreviations

ANC: Antenatal Care, AOR: Adjusted Odds Ratio, CI: Confidence Interval, COR: Crude Odds Ratio, CSA: Central Statistical Agency, EA: Enumeration Areas, EDHS: Ethiopian Demographic and Health Survey, FAO: Food and Agriculture Organizations, FMoH: Federal Ministry of Health, ICC: Intra-class Correlation Coefficients, MN: Micronutrients, PNC: Postnatal Care, and WHO: World Health Organization

Acknowledgments

We are very thankful to MEASURE DHS for permission to use the EDHS 2016 survey data sets.

References

1. Von Grebmer K, Saltzman A, Birol E, Wiesman D, Prasai N, Yin S, et al. Synopsis: 2014 Global Hunger Index: The Challenge of Hidden Hunger: Intl Food Policy Res Inst; 2014.
2. Tzioumis E, Kay MC, Bentley ME, Adair LS. Prevalence and trends in the childhood dual burden of malnutrition in low-and middle-income countries, 1990–2012. Public health nutrition. 2016;19(8):1375-88.
3. Harika R, Faber M, Samuel F, Kimiywe J, Mulugeta A, Eilander A. Micronutrient status and dietary intake of iron, vitamin A, iodine, folate and zinc in women of reproductive

age and pregnant women in Ethiopia, Kenya, Nigeria and South Africa: a systematic review of data from 2005 to 2015. Nutrients. 2017;9(10):1096.

4. Azadbakht L, Esmaillzadeh A. Macro and Micro-Nutrients Intake, Food Groups Consumption and Dietary Habits among Female Students in Isfahan University of Medical Sciences. Iranian Red Crescent medical journal. 2012;14(4):204-9. Epub 2012/07/04. PubMed PMID: 22754682; PubMed Central PMCID: PMCPMC3385798.

5. Bush LA, Hutchinson J, Hooson J, Warthon-Medina M, Hancock N, Greathead K, et al. Measuring energy, macro and micronutrient intake in UK children and adolescents: a comparison of validated dietary assessment tools. BMC Nutrition. 2019;5(1):53. doi: 10.1186/s40795-019-0312-9.

6. Herrador Z, Sordo L, Gadisa E, Buño A, Gómez-Rioja R, Iturzaeta JM, et al. Micronutrient deficiencies and related factors in school-aged children in Ethiopia: a cross-sectional study in Libo Kemkem and Fogera districts, Amhara Regional State. PLoS One. 2014;9(12):e112858. Epub 2014/12/30. doi: 10.1371/journal.pone.0112858. PubMed PMID: 25546056; PubMed Central PMCID: PMCPMC4278675.

7. Sharma P, Dwivedi S, Singh D. Global poverty, hunger, and malnutrition: a situational analysis. Biofortification of food crops: Springer; 2016. p. 19-30.

8. Tariku A, Bikis GA, Woldie H, Wassie MM, Worku AG. Child wasting is a severe public health problem in the predominantly rural population of Ethiopia: A community based cross–sectional study. Archives of Public Health. 2017;75(1):26.

9. Bharaniidharan J, Reshmi S. Review on Malnutrition: Impact and Prevention. International Journal. 2019;7(3):240-3.

10. Ritchie H, Roser M. Micronutrient deficiency. Our World in data. 2017.

11. Ames BN. Low micronutrient intake may accelerate the degenerative diseases of aging through allocation of scarce micronutrients by triage. Proceedings of the National Academy of

Sciences of the United States of America. 2006;103(47):17589-94. Epub 2006/11/15. doi: 10.1073/pnas.0608757103. PubMed PMID: 17101959.

12. Schneider J, Fumeaux CJF, Duerden EG, Guo T, Foong J, Graz MB, et al. Nutrient intake in the first two weeks of life and brain growth in preterm neonates. Pediatrics. 2018;141(3):e20172169.

13. Velasco I, Bath SC, Rayman MP. Iodine as essential nutrient during the first 1000 days of life. Nutrients. 2018;10(3):290.

14. Keeley B, Little C, Zuehlke E. The State of the World's Children 2019: Children, Food and Nutrition--Growing Well in a Changing World. UNICEF. 2019.

15. Ahmed F, Prendiville N, Narayan A. Micronutrient deficiencies among children and women in Bangladesh: progress and challenges. Journal of nutritional science. 2016;5:e46. Epub 2017/06/18. doi: 10.1017/jns.2016.39. PubMed PMID: 28620473; PubMed Central PMCID: PMCPMC5465809.

16. Nunn RL, Kehoe SH, Chopra H, Sahariah SA, Gandhi M, Di Gravio C, et al. Dietary micronutrient intakes among women of reproductive age in Mumbai slums. European journal of clinical nutrition. 2019;73(11):1536-45. doi: 10.1038/s41430-019-0429-6.

17. Kennedy GL, Pedro MR, Seghieri C, Nantel G, Brouwer I. Dietary Diversity Score Is a Useful Indicator of Micronutrient Intake in Non-Breast-Feeding Filipino Children. The Journal of nutrition. 2007;137(2):472-7. doi: 10.1093/jn/137.2.472.

18. Newmann SJ, Mishra K, Onono M, Bukusi EA, Cohen CR, Gage O, et al. Providers' Perspectives on Provision of Family Planning to HIV-Positive Individuals in HIV Care in Nyanza Province, Kenya. AIDS Res Treat. 2013;2013:915923. doi: 10.1155/2013/915923. PubMed PMID: 23738058; PubMed Central PMCID: PMCPMC3659431.

19. Lander RL, Enkhjargal T, Batjargal J, Bailey KB, Diouf S, Green TJ, et al. Multiple micronutrient deficiencies persist during early childhood in Mongolia. Asia Pacific journal of clinical nutrition. 2008;17(3):429-40. Epub 2008/09/27. PubMed PMID: 18818163.

20. Sheehy T, Carey E, Sharma S, Biadgilign S. Trends in energy and nutrient supply in Ethiopia: a perspective from FAO food balance sheets. Nutrition journal. 2019;18(1):46. doi: 10.1186/s12937-019-0471-1.

21. Central Statistical Agency Addis Ababa E. Ethiopia Demographic and Health Survey 2016 Addis Ababa, Ethiopia 2016.

22. Dangura D, Gebremedhin S. Dietary diversity and associated factors among children 6-23 months of age in Gorche district, Southern Ethiopia: Cross-sectional study. BMC pediatrics. 2017;17(1):6.

23. Birhanu M, Abegaz T, Fikre R. Magnitude and Factors Associated with Optimal Complementary Feeding Practices among Children Aged 6-23 Months in Bensa District, Sidama Zone, South Ethiopia. Ethiopian journal of health sciences. 2019;29(2).

24. Forsido SF, Kiyak N, Belachew T, Hensel O. Complementary feeding practices, dietary diversity, and nutrient composition of complementary foods of children 6–24 months old in Jimma Zone, Southwest Ethiopia. Journal of Health, Population and Nutrition. 2019;38(1):14.

25. Mulat E, Alem G, Woyraw W, Temesgen H. Uptake of minimum acceptable diet among children aged 6–23 months in orthodox religion followers during fasting season in rural area, DEMBECHA, north West Ethiopia. BMC Nutrition. 2019;5(1):18.

26. Sagaro GG AM. Dietary Diversity and Associated Factors Among Infants and Young Children in Wolaita Zone, Southern Ethiopia. Science Journal of Clinical Medicine. July 2017;6, , (4,): 53-9.

27. Belay A, Joy EJ, Chagumaira C, Zerfu D, Ander EL, Young SD, et al. Selenium Deficiency Is Widespread and Spatially Dependent in Ethiopia. Nutrients. 2020;12(6):1565.

28. Herrador Z, Sordo L, Gadisa E, Buño A, Gómez-Rioja R, Iturzaeta J, et al. Micronutrient Deficiencies and Related Factors in School-Aged Children in Ethiopia: A Cross-Sectional Study in Libo Kemkem and Fogera Districts, Amhara Regional State. PloS one. 2014;9:e112858. doi: 10.1371/journal.pone.0112858.

29. Shivakoti R, Christian P, Yang WT, Gupte N, Mwelase N, Kanyama C, et al. Prevalence and risk factors of micronutrient deficiencies pre- and post-antiretroviral therapy (ART) among a diverse multicountry cohort of HIV-infected adults. Clinical nutrition. 2016;35(1):183-9. Epub 2015/02/24. doi: 10.1016/j.clnu.2015.02.002. PubMed PMID: 25703452; PubMed Central PMCID: PMCPMC4531105.

30. Serra-Majem L, Ribas L, Pérez-Rodrigo C, García-Closas R, Peña-Quintana L, Aranceta J. Determinants of nutrient intake among children and adolescents: results from the enKid Study. Annals of Nutrition and Metabolism. 2002;46(Suppl. 1):31-8.

31. Cheng Y, Dibley MJ, Zhang X, Zeng L, Yan H. Assessment of dietary intake among pregnant women in a rural area of western China. BMC Public Health. 2009;9(1):222. doi: 10.1186/1471-2458-9-222.

32. Cetin I, Bühling K, Demir C, Kortam A, Prescott SL, Yamashiro Y, et al. Impact of Micronutrient Status during Pregnancy on Early Nutrition Programming. Annals of Nutrition and Metabolism. 2019;74(4):269-78. doi: 10.1159/000499698.

33. Birhanu M, Abegaz T, Fikre R. Magnitude and Factors Associated with Optimal Complementary Feeding Practices among Children Aged 6-23 Months in Bensa District, Sidama Zone, South Ethiopia. Ethiop J Health Sci. 2019;29(2):153-64. Epub 2019/04/24. doi: 10.4314/ejhs.v29i2.2. PubMed PMID: 31011263; PubMed Central PMCID: PMCPMC6460456.

34. Iannotti L. Dietary Intakes and Microntrient Adequacy Related to the Changing Livelihoods of Two Pastoralist Communities in Samburu, Kenya. Current Anthropology. 2014;55:475-82. doi: 10.1086/677107.

35. Bach A, Gregor E, Sridhar S, Fekadu H, Fawzi W. Multisectoral Integration of Nutrition, Health, and Agriculture: Implementation Lessons From Ethiopia. Food and nutrition bulletin. 2020:0379572119895097.

36. Haile D, Biadgilign S, Azage M. Differentials in vitamin A supplementation among preschool-aged children in Ethiopia: evidence from the 2011 Ethiopian Demographic and Health Survey. Public health. 2015;129(6):748-54.

37. Gebre-Egziabhere T. Emerging Regions in Ethiopia: Are they catching up with the rest of Ethiopia? Eastern Africa Social Science Research Review. 2018;34(1):1-36.

38. Stark J, Terasawa K, Ejigu M. Climate change and conflict in pastoralist regions of Ethiopia: mounting challenges, emerging responses. Conflict Management and Mitigation (CMM) Discussion Paper. 2011;(4).

39. Central Statistical Agency (CSA) [Ethiopia] and ICF. Ethiopia Demographic and Health Survey 2016. Addis Ababa, Ethiopia, and Rockville, Maryland, USA: CSA and ICF. 2016. https://dhsprogram.com. 2016.

40. WHO. Use of multiple micronutrient powders for point- of- use fortification of foods consumed by infants and young children aged 6–23 months and children aged 2–12 years guideline2016. 60. https://www.who.int/nutrition/publications/micronutrients/guidelines/mmpowders-infant6to23mons-children2to12yrs/en/ p.

41. Dary O, Hurrell R. Guidelines on food fortification with micronutrients. World Health Organization, Food and Agricultural Organization of the United Nations: Geneva, Switzerland. 2006.

42. FAO, WHO. Human vitamin and mineral requirements. Report of a joint FAO/WHO expert consultation, Bangkok, Thailand. Food and Nutrition Division, FAO, Rome. 2001:235-47.

43. WHO. Protein and amino acid requirements in human nutrition: report of a joint FAO/WHO/UNU expert consultation. Geneva; 2007. WHO Technical Report Series. 935.

44. Organization WH. WHO guideline: use of multiple micronutrient powders for point-of-use fortification of foods consumed by infants and young children aged 6–23 months and children aged 2–12 years: World Health Organization; 2016. 60 p.

45. Russell R, Beard JL, Cousins RJ, Dunn JT, Ferland G, Hambidge K, et al. Dietary reference intakes for vitamin A, vitamin K, arsenic, boron, chromium, copper, iodine, iron, manganese, molybdenum, nickel, silicon, vanadium, and zinc. A Report of the Panel on Micronutrients, Subcommittees on Upper Reference Levels of Nutrients and of Interpretation and Uses of Dietary Reference Intakes, and the Standing Committee on the Scientific Evaluation of Dietary Reference Intakes Food and Nutrition Board Institute of Medicine. 2001.

46. Tabacchi G, Wijnhoven TM, Branca F, Roman-Vinas B, Ribas-Barba L, Ngo J, et al. How is the adequacy of micronutrient intake assessed across Europe? A systematic literature review. The British journal of nutrition. 2009;101 Suppl 2:S29-36. Epub 2009/07/15. doi: 10.1017/s0007114509990560. PubMed PMID: 19594962.

47. Croft TN, Aileen M. J. Marshall, Courtney K. Allen, et al.,. Guide to DHS Statistics. Rockville, Maryland, USA: ICF. 2018.

48. Hox JJ, Moerbeek M, Van de Schoot R. Multilevel analysis: Techniques and applications. Third ed: Routledge; 2010. 364. https://b-ok.cc/book/3695027/0a101f p.

49. Central Statistical Agency (CSA) [Ethiopia] and ICF. 2016. Ethiopia Demographic and Health Survey 2016 2016. Addis Ababa, Ethiopia, and Rockville, Maryland, USA: CSA and ICF,].

50. The Ethiopian Public Health Institute. Ethiopian National Micronutrent Survey Report 2016. Available from: https://www.ephi.gov.et/images/pictures/download2009/National_MNS_report.pdf.

51. Aghaji AE, Duke R, Aghaji UC. Inequitable coverage of vitamin A supplementation in Nigeria and implications for childhood blindness. BMC public health. 2019;19(1):282.

52. Agrawal S, Agrawal P. Vitamin A supplementation among children in India: Does their socioeconomic status and the economic and social development status of their state of residence make a difference? International journal of medicine and public health. 2013;3(1):48.

53. The Federal Democratic Republic of Ethiopia. National Nutrition Programme: June 2013–June 2015, MOH, Addis Ababa, Ethiopia 2012. Available from: https://extranet.who.int/nutrition/gina/sites/default/files/ETH%202013%20National%20Nutrition%20Programme.pdf.

54. Ayana G, Hailu A, Tessema M, Belay A, Zerfu D, Bekele T, et al. Ethiopian National Nutrition Program End-Line Survey2015.

55. Mengistu G, Moges T, Samuel A, Baye K. Energy and nutrient intake of infants and young children in pastoralist communities of Ethiopia. Nutrition. 2017;41:1-6.

56. Tassew AA, Tekle DY, Belachew AB, Adhena BM. Factors affecting feeding 6–23 months age children according to minimum acceptable diet in Ethiopia: A multilevel analysis of the Ethiopian Demographic Health Survey. PloS one. 2019;14(2).

57. Aremu O, Lawoko S, Dalal K. Childhood vitamin A capsule supplementation coverage in Nigeria: a multilevel analysis of geographic and socioeconomic inequities. The Scientific World Journal. 2010;10:1901-14.

58. Aemro M, Mesele M, Birhanu Z, Atenafu A. Dietary diversity and meal frequency practices among infant and young children aged 6–23 months in Ethiopia: a secondary analysis

of Ethiopian demographic and health survey 2011. Journal of nutrition and metabolism. 2013;2013.

59. Temesgen H, Negesse A, Woyraw W, Mekonnen N. Dietary diversity feeding practice and its associated factors among children age 6–23 months in Ethiopia from 2011 up to 2018: a systematic review and meta-analysis. Italian journal of pediatrics. 2018;44(1):1-10.

60. Immurana M, Arabi U. Socio-economic covariates of micronutrients supplementation and deworming among children in Ghana. J Behav Health. 2016;4:154-61.

61. Ickowitz A, Rowland D, Powell B, Salim MA, Sunderland T. Forests, trees, and micronutrient-rich food consumption in Indonesia. PloS one. 2016;11(5).

Figure Click here to access/download;Figure;Figure 1.docx

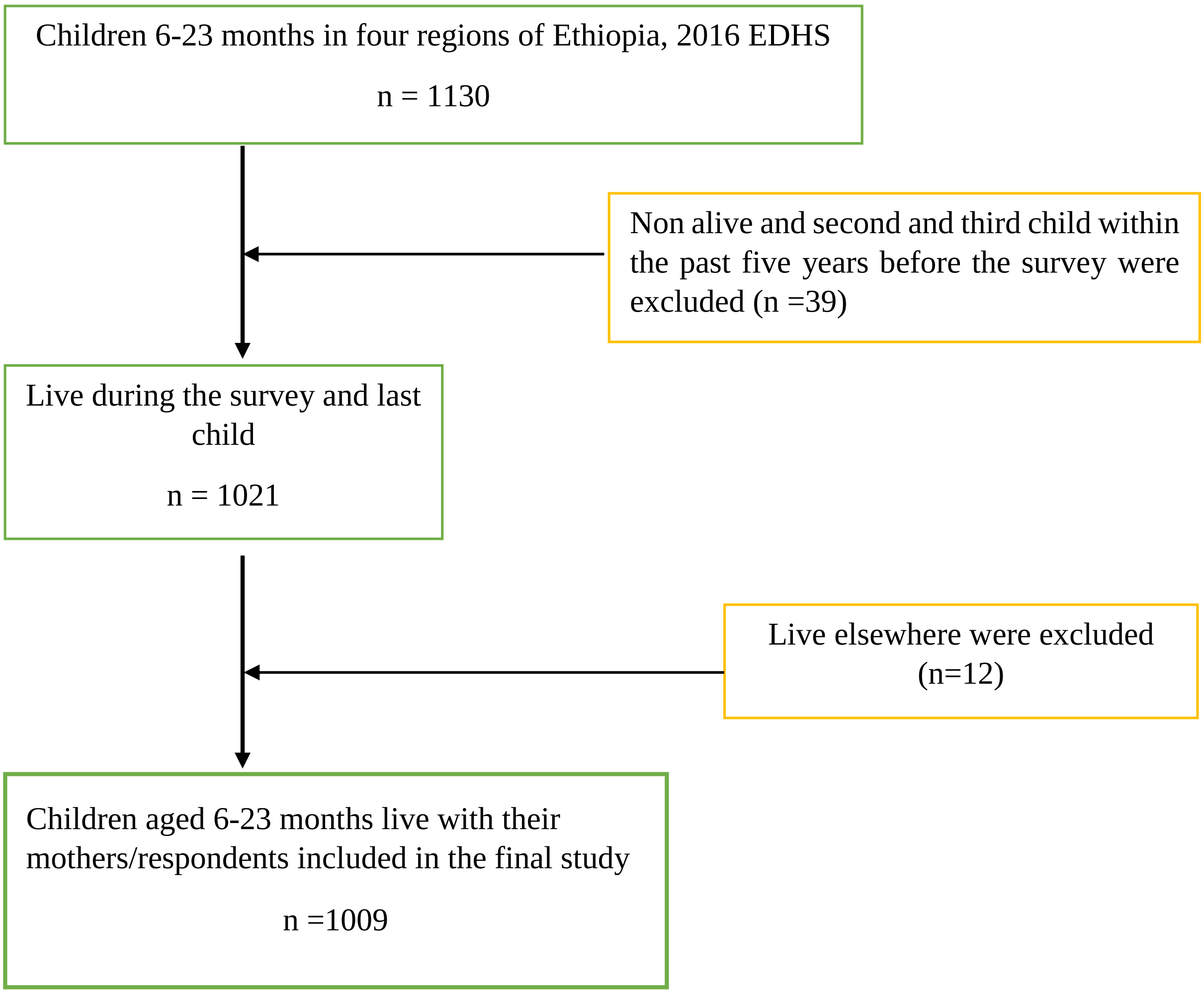

Figure 1. Sampling and exclusion procedures to identify children aged 6-23 months in the emerging regions of Ethiopia, EDHS 2016

Micronutrient intake status and associated factors among children aged 6-23 months in the emerging regions of Ethiopia: a multilevel analysis of the 2016 Ethiopian demographic and health survey

Tsegaye Gebremedhin*, Andualem Yalew Aschalew, Chalie Tadie, Endalkachew Dellie, and Asmamaw Atnafu

Department of Health Systems and Policy, Institute of Public Health, College of Medicine and Health Sciences, University of Gondar, Gondar, Ethiopia

***Corresponding author**

Email: ~~tsegishg27@gmail.com~~tsegishg27@gmail.com

Abstract

Background: Micronutrient deficiency is recognized as a major public health problem in developing countries, including Ethiopia. The scarcity of micronutrients, particularly in pastoral communities of Ethiopia, might be severe due to poor health care access, drought and poverty. However, empirical evidence is scarce in Ethiopia. Therefore, ~~the aim of~~ this study ~~was~~aimed to assess the micronutrient intake status of children aged 6 to 23 months in the emerging regions of Ethiopia.

Methods: Data from the 2016 Ethiopia Demographic and Health Survey (EDHS) were used. A two-stage stratified sampling technique was employed to identify 1009 children aged 6 to 23 months. A multilevel mixed-effect logistic regression analysis was used to identify individual and community-level factors associated with micronutrient intake status. In the final model, variables with a p-value of < 0.05 and adjusted odds ratio (AOR) with 95% confidence interval (CI) were used to identify factors statistically associated with micronutrient intake status.

Results: Overall, 62.7% (95% CI: 59.7-65.7) of children aged 6 to 23 months had received at least one of the ~~minimum~~ recommended micronutrients. Antenatal care (ANC) visit (AOR: 1.95, 95% CI: 1.37-2.77), work in the agriculture (AOR: 2.22, 95% CI: 1.30-3.80~~),~~) and children aged 13 to 23 months (AOR: 1.72, 95% CI: 1.25-2.36) were the individual-level factors, whereas rural residence (AOR: 0.37, 95% CI: 0.14-0.88), reside in Benishangul (AOR: 2.25, 95% CI: 1.29-4.93) and Gambella regions (AOR: 1.87, 95% CI: 1.03-3.38) ~~-~~were the community-level factors associated with micronutrient intake status.

~~Conclusion~~Conclusions: The micronutrient intake status in the study area was low compared to the national recommendation. Promoting vitamin A and iron~~-~~-rich foods and micronutrient powders at individual and community-level and strengthen supplementations and deworming

alongside the community-based maternal and child health services would improve the micronutrient intake among children.

Introduction

Micronutrient deficiency is recognized as a global public health problem among children, and it is worse in low- and middle-income countries, particularly in Ethiopia [1-3].

The commonest micronutrients needed for life are iron, zinc, calcium, iodine, and vitamins, which can be found from foods rich in vitamins and minerals or in the form of supplements, such as vitamin A supplement [4-6]. Although micronutrients are only needed in small quantities, their absence from children's diet negatively affects the survival and development of children. Micronutrient deficiency may lead to devastating consequences, like stunting, wasting, weakened immunity, and ~~delays~~delay in cognitive development [7-11]. Notably, micronutrient is very critical during the first two years of a child's life; as adequate nutrition during this period promotes healthy growth and development. Yet, micronutrient deficiency received less attention than the apparent starvation of people who are unable to get enough food to survive [12, 13].

Furthermore, most infants and young children around the world are not getting the healthy diets that they need for life and grow well. According to the United Nations Children's Fund (UNICEF) 2019 report, there were around 340 million children worldwide who suffered from hidden hunger caused by deficiencies of vitamins and minerals [14]. Globally, a million child deaths per year due to vitamin A and zinc deficiencies were reported in 2016 [15-17]. Nevertheless, the magnitude of the problem is much higher in low- and middle-income countries~~,~~; only 29% of children aged 6–23 months were fed the minimum diversified diet ~~that is~~ needed for growth and development [18].

Similarly, the deficiency of crucial vitamins and mineral are among the significant public health problems in Ethiopia. ~~The~~These deficiencies are a result of diets with limited diversity, minimal bioavailability, frequent meal skipping, limited access to micronutrient-rich and

fortified foods, and low vegetable and fruit intake [15, 19, 20]. According to the Ethiopian Demographic and Health Survey (EDHS) 2016 report, only 14% of the children aged 6–23 months received the minimum dietary diversity [21]. Moreover, studies in different parts of the country reported only 13 to 43.2% of children aged 6-23 months consumed a diversified diet [22-26]. The Ethiopian national nutritional supplementation survey (2016) indicated that vitamin A supplementation coverage in children was 63%, which is lower than the national target (more than 90%) and vitamin A deficiency was significant (14%) [27].

Micronutrient intake is associated with ~~a variety of~~various factors at individual and community levels, including mothers' sociodemographic and child characteristics, dietary habits, community-level lifestyle and place of residence [28-30]. On top of the above factors, the use of maternal healthcare services, such as antenatal care (ANC), institutional delivery and postnatal care (PNC) visits, are also associated with the micronutrient intake status of children [31, 32].

Although there is documented evidence of insufficient micronutrient intake for agrarian communities and urban dwellers in Ethiopia [22, 24, 25, 33], there is little evidence on micronutrient intake among children aged 6-23 months in emerging regions of Ethiopia where pastoralist communities, with poor cultivation of fruits and vegetables, are mainly reside [34]. Additionally, these regions have been identified as the hotspot in the country with high food insecurity, high child malnutrition rates, and the recurrent onset of droughts [35, 36].

Besides, these areas have limited access to health facilities, ~~low~~poor infrastructure and inaccessible health services that could worsen the micronutrient deficiency [37, 38]. However, studies that show the individual and community-level factors of micronutrient intake among children are rare. The objective of this study is, therefore, to assess the micronutrient intake status and related factors among children aged 6-23 months in emerging regions of Ethiopia by

Formatted: Font color: Text 1

Formatted: Font color: Text 1

Formatted: Font color: Text 1

Formatted: Font color: Text 1

Formatted: Font color: Text 1

Formatted: Font color: Text 1

Formatted: Font color: Text 1

Formatted: Font color: Text 1

Formatted: Font color: Text 1

Formatted: Font color: Text 1

Formatted: Font color: Text 1

Formatted: Font color: Text 1

using the 2016 EDHS data. Moreover, the finding could give important insights to develop contextual strategies for the mitigation of the problem.

Materials and Methods

Study settings and data source

The study used the EDHS 2016 data, ~~which is~~ a nationally representative household survey data collected every five years. It has been implemented by the Central Statistical Agency (CSA) [39] with the primary objective of providing up-to-date estimates of key demographic and health indicators. Administratively, Ethiopia is divided into nine and two geographical regions and administrative cities, respectively. Of the total regional states, Afar, Somali, Benishangul, and Gambela are the emerging regions of Ethiopia where pastoralists predominantly live.

Sampling procedures

The sampling frame for the 2016 EDHS used the 2007 Ethiopian population and housing census, which was conducted by the CSA of Ethiopia. The census used a complete list of 84,915 enumeration areas (EAs), which contains the location, type of residence, and the estimated number of residential households. Then the sample for the 2016 EDHS was stratified in two stages, and samples of EAs were selected independently from each stratum. The regions were stratified into urban and rural areas. At each of the lower administrative levels, implicit stratification and proportional allocation were achieved within each sampling stratum before sample selection at different levels.

In the first stage, 645 EAs were selected with probability proportional to the EA size, and each sampling stratum was selected from the given samples. The total residential households in the EA were the EA size, and a household listing operation was implemented. Then, the resulting

lists of households were used as the sampling frame for the selection of households in the second stage. Some of the selected EAs were large. The selected large EAs with more than 200 households were segmented to minimize the task of household listing. Through the probability proportional to the segment size, only a segment was selected for the survey, and the household listing was conducted for each selected segment.

A fixed number of 28 households from each cluster were selected with an equal probability in the second stage, a systematic selection from the newly created household listing. The survey interviewer interviewed only pre-selected households. No replacements or changes of the pre-selected households were allowed in the implementing stages to prevent bias. In this study, the 2016 EDHS childhood datasets of the four emerging regional states: Afar, Benishangul, Gambella~~,~~ and Somali, were used for analysis.

All women aged 15-49 years who are the usual members of the selected households were eligible for the female survey. Children aged 6–23 months were the source population and included 1009 mothers and their children aged 6-23 months were included in the analysis. Alive, ~~index to birth history first~~last, child~~,~~ and live with their mothers/caregivers were included, while those second and above ~~index to birth history~~child and live with other than their mothers/caregivers were excluded from the analysis (Fig 1). Additionally, for twins, a mother was asked for a child only. Potential individual and community level independent variables were also extracted and further analysis was done.

Formatted: Highlight

Fig 1. Sample study selection of children age 6-23 months in emerging regions, EDHS 2016

Measurements of variables

The dependent variable of the study was micronutrient intake status among children aged 6-23 months, which was determined from the reports of mothers regarding the routine micronutrient intake of their children. The intake status was measured based on the recommendations of daily micronutrient intake that meets the micronutrient requirements of almost all healthy individuals in age and sex-specific population groups [40-43]. The recommended micronutrient for children are foods rich in vitamin A within 24 hours, foods rich in iron within 24 hours, multiple micronutrient powder within seven days, iron supplements within seven days, vitamin A supplements within six months, or deworming medication within six months [41, 42, 44-46]. Accordingly, if the children received at least one of the minimum recommended micronutrients, we considered as "Yes"; if the children received none of the minima recommended micronutrients, it was considered as "No".

Foods rich in vitamin A were measured by the consumption of either of the seven food groups within the preceding 24 hours. These food groups were I. Eggs, ii. Meat (beef, pork, lamb, chicken), iii. pumpkin, carrots, and squash, iv. any dark green leafy vegetables, v. mangoes, papayas, and others with vitamin A fruits, vi. Liver, heart, and other organs and vii. Fish or shellfish.

Foods rich in iron were measured by the consumption of either of the four iron-rich food groups within the past 24 hours. These groups were i. eggs, ii. meat (beef, pork, lamb, chicken), iii. Liver, heart, and other organs, and iv. Fish or shellfish.

Multiple micronutrient powders were assessed by asking the mother whether their child had received micronutrient powders in the previous seven days. The micronutrients contained a

range of vitamins and minerals (and nearly always include iron, per WHO recommendations) enclosed in a single-dose sachet added to foods; Sprinkles™ is a brand name used for micronutrient powders.

~~The iron~~Iron supplementation was assessed by asking the mothers whether their child had iron supplementation defined as iron pills, sprinkles with iron, or iron syrup in the previous seven days.

Vitamin A supplementation and deworming medication have been provided for children aged 6-59 months by semi-annually as a national nutrition program. For this study, vitamin A supplementation and deworming were assessed for those 6 to 23 months of children whether they received for the last six months or not by reviewing the integrated child card, which consists of immunization and growth monitoring history.

Individual and community-level characteristics were the independent variables. The individual-level variables were individual sociodemographic and economic characteristics, obstetric history of the women, and child characteristics. ~~Whereas~~At the same time, the community-level variables were residence, region, community-level poverty, community-level media exposure, the distance of the nearest health facility.

~~Wealth~~The wealth quintile was calculated as an index based on consumer goods such as television, bicycle, or car. Household characteristics were also considered in calculating the wealth index. These scores were derived using principal component analysis and ranked into poor, middle, and rich. The wealth quintiles are expressed in terms of quintiles of individuals in the population rather than quintiles of individuals at risk for anyone's health or population indicator.

Distance to the health facility was assessed by the question “distance to the nearest health facility is a problem?” and the responses were categorized as “big problem” or “not a problem” [47].

Community-level poverty was assessed using the asset index based on data from the entire country sample on separate scores prepared for rural and urban households, and combined to produce a single asset index for all households as the community level and ranked into five (poorest, poorer, middle, richer, and richest).

Community media exposure was assessed as “yes” if they have access to all three media (newsletter, radio, and television) at least once a week, otherwise “no” if they did not have any media exposure.

Data processing and statistical analysis

The data were cleaned, re-coded and analysed using STATA (StataCorp, College Station, TX) version 14. Descriptive statistics were presented using tables and narration to describe the magnitude of micronutrient intake status by sociodemographic, maternal obstetric and child characteristics.

A multilevel analysis was conducted after checking the eligibility. The model eligibility was assessed by calculating the Intra-class Correlation Coefficient (ICC) and model with ICC greater than 10% is eligible for multilevel analysis. In this study, the ICC was 27.3%. Since the data were hierarchical (individuals were nested within communities), a two-level mixed-effects logistic regression model was fitted to estimate both the individual and community level variables (fixed and random) effect on micronutrient intake status, and the log of the probability of micronutrient intake was modelled using the formula as follows [48]:

$$log\ [\frac{\pi ij}{1-\pi ij}] = \beta_o + \beta_1 X_{ij} + \beta_2 Z_{ij} + U_j + e_{ij}$$

Where i is individual level unit and j is a community-level unit; X and Z refer to individual and community-level variables, respectively; πij is the probability of micronutrient intake for the ith child in the jth community; the β's are the fixed coefficients. Whereas, β_0 is the intercept-the effect on the probability of micronutrient intake in the absence of influence of predictors; and u_j showed the effect of the community (random effect) on micronutrient intake for the jth community and e_{ij} showed random errors at the individual levels. By assuming each community had different intercept ($\beta_0 + U_j$) and fixed coefficient ($\beta_{1,2}$), the clustered data nature and the within and between community variations were taken into account.

Bivariable and multivariable analysis were computed. First, in the bivariable logistic regression analysis, a p-value of less than 0.2 was used to fitted three models (models for the individual level, community level, and both the individual and community level). Then, in the final model (fixed effect), a p-value less than 0.05 and adjusted odds ratio (AOR) with 95% confidence interval (CI) were used to estimate the association of individual and community level factors with micronutrient intake status.

The measures of variation (random-effects) were reported using ICC and proportional change in variance (PCV) to measure the variation between clusters. The ICC refers to the ratio of the between-cluster variance to the total variance, and it tells us the proportion of the total variance in the outcome variable that is accounted at the cluster level. The loglikelihood test was used to estimate the goodness of fit of the adjusted final model in comparison to the preceding models. A model with the smallest value of loglikelihood is better; accordingly, model three (a model for both the individual and community level variables) had the lowest value.

Ethical considerations

The ethical approval and permission to access the data were obtained from the MEASURE DHS (available from https://www.dhsprogram.com/Data/: accessed on April 06, 2020) after a brief study concept was submitted.

Results

Sociodemographic and economic characteristics of participants

A total of 1009 mothers/caregivers with children aged 6-23 months were included in the final analysis. The mean age of the mothers was 27.5 (SD ± 6.3) years, and 83.9% of them were rural dwellers. The majority (72.4%) of the households were in the poor wealth status; the mean family size was 5.9 (SD± 2.3). Nearly one-third (34.3%) of the participants were from the Somali region and the majority (71.1%) were Muslim by religion. Of the participants, 95.2% were married; 59.3% with no education and 66.5% had no work. Similarly, 59.3 and 46.6% of their husbands/partners had no education and agricultural workers, respectively (Table 1).

Table 1. Sociodemographic and economic characteristics of study participants in emerging regions of Ethiopia, 2016 (n=1009).

Formatted: Font: Not Bold

Variables	Category	Frequency (n)	Percent (%)
Age of mothers/caregivers in ~~complete~~ years	15-24	341	33.8
	25-34	497	49.3
	>=35	171	16.9
Residence	Urban	163	16.1
	Rural	846	83.9
Region	Afar	254	25.2
	Somali	346	34.3
	Benishangul	224	22.2
	Gambela	185	18.3
Religion	Muslim	717	71.1
	Protestant	173	17.1
	Orthodox	85	8.4
	Others*	34	3.4
Sex of ~~head of~~ household head	Male	674	66.8
	Female	335	33.2
Household wealth status	Poor	730	72.4

Variables	Category	Frequency (n)	Percent (%)
	Middle	78	7.7
	Rich	201	19.9
Current marital status	Married	961	95.2
	Unmarried	48	4.8
Educational status of mothers/caregivers	No education	715	70.9
	Primary education	199	19.7
	Secondary education	67	6.6
	Higher	28	2.8
Educational status of husband’s/partner’s (n=961)	No education	570	59.3
	Primary education	197	20.5
	Secondary education	104	10.8
	Higher	90	9.4
Respondent's occupation	No work	671	66.5
	Professional worker	80	7.9
	Agricultural worker	189	18.7
	Others**	69	6.8
Husband’s/partner’s occupation (n=961)	No work	139	14.5
	Professional worker	175	18.2
	Agricultural worker	448	46.6
	Others***	199	20.7

*Catholic, traditional, Joba,

**Daily labor, merchant

***Daily labor, merchant

Obstetric history of participants

The majority (54.5%) of women were in the age group of 18 to 24 years when they gave their first birth. Of the total women, 45.2% of them were multipara, and 59.9% have two to five living children. Nearly 56% of the women had ANC visits for their recent birth, and out of them, 55.9% had first visit. Furthermore, only one-fourth of the women delivered at health facilities, and 7.2% of them had PNC checks within two months after delivery (Table 2).

Table 2. Obstetric characteristics of participants in the emerging regions of Ethiopia, 2016 (n=1009).

Variables	Category	Frequency (n)	Percent (%)
Age of the mother at first birth (in years)	<18	402	39.8
	18-24	550	54.5
	25+	57	5.7
Parity	Primipara	176	17.4
	Multipara	456	45.2
	Grand multipara	377	37.4
Number of living children	1	174	17.2
	2-5	604	59.9
	6+	231	22.9
ANC visit	Yes	569	56.4
	No	440	43.6
Desire for more children	Wants	798	79.1
	Undecided	43	4.3
	Wants no more	168	16.6
Place of delivery	Home	745	73.8
	Health facility	264	26.2
PNC check within two months	Yes	73	7.2
	No	936	92.8
Timing after postnatal delivery check took place (n=73)	Within 24hrs	7	9.6
	1-7th day	41	56.2
	After the 7th day	25	34.2
Current pregnancy status	Pregnant	90	8.9
	Non-pregnant	919	91.1

ANC: Antenatal care, PNC: Postnatal care

Child characteristics and common childhood illness

Of the total children, 54.1% were male and 54.2% were in the age group of 13 to 23 months. Moreover, 42.0% of the children had average birth weight;55.8% of the children were found between the second and fifth-order of birth. Approximately 96% of the children were measured their current weight, and of those, 79.0% were weighted 7-11kgs. Additionally, 15.9, 13.6, and

15.8% of the children had diarrhea, cough, and fever within the last two weeks, respectively (Table 3).

Table 3. Child characteristics and common childhood illness among children aged 6 to 23 months in the emerging regions of Ethiopia, 2016 (n=1009).

Formatted: Font: Not Bold

Variables	Category	Frequency (n)	Percent (%)
Sex of the child	Male	546	54.1
	Female	463	45.9
The current age of the child in months	6-12	462	45.8
	13-23	547	54.2
Size of a child at birth	Large	269	26.7
	Average	424	42.0
	Small	316	31.3
Birth order	1	176	17.4
	2-5	563	55.8
	6+	270	26.8
Preceding birth interval in months (n=833)	<=24	241	28.9
	25-36	282	33.8
	37-48	163	19.6
	>=49	147	17.7
Current weight of the child measured	Yes	964	95.6
	No	45	4.4
Current weight of child in kg (n=964)	<7	145	15.0
	7-11	762	79.0
	11+	57	6.0
Had diarrhoea	Yes	160	15.9
	No	849	84.1
Had cough	Yes	137	13.6
	No	872	86.4
Had fever	Yes	159	15.8
	No	850	84.2

Community-level poverty, media exposure and access to a health facility

The analysis showed that 63.1, 16.1, 9.3, 7.3 and 4.2% of the communities are in the poorest, poorer, middle, richer and richest status, respectively. Only five percent of the community has media exposure. Additionally, 42.0% of the respondents mentioned that access to a health facility is not a problem.

Micronutrient intake status among children aged 6-23 months

Overall, 62.7% (95% CI: 59.7-65.7) of the children received the minimum recommended micronutrients. Of those who took the recommended micronutrient, only 12.9% had received three and above micronutrient types.

Moreover, 27.8% (95% CI: 25.0-30.5) of the children consumed foods rich in vitamin A at any time in 24 hours preceding the interview, and 15.6% (95% CI: 13.3-17.8) of them have consumed foods rich in iron at any time in 24 hours preceding the interview. Additionally, 7.5% (95% CI: 5.9-9.2) of the children received multiple micronutrient powder in the seven days preceding the interview, and 6.0% (95% CI: 4.5-7.4) of them received iron supplements in the seven days preceding the interview. Around 47% (95% CI: 44.1-50.3) of the children received vitamin A supplements in the six months preceding the interview. Besides, 8.4% (95% CI: 5.5-8.7) of the children aged 12 to 23 months of were received deworming medication in the six months preceding the interview (Table 4).

Table 4. Micronutrient intake status among children aged 6-23 months in the emerging regions of Ethiopia, 2016 (n=1009).

Formatted: Font: Not Bold

Food groups and supplementations	Contains/measurements	Received n (%)	No received n (%)
Consumed foods rich in vitamin A within 24 hours	Eggs	85 (8.4)	924 (91.6)
	Meat (beef, pork, lamb, chicken, etc)	52 (5.2)	957 (94.8)
	Pumpkin, carrots, and squash	111 (11.0)	898 (89.0)
	Any dark green leafy vegetables	91 (9.0)	918 (91.0)
	Mangoes, papayas, and others with vitamin A fruits	133 (13.2)	876 (86.8)
	Liver, heart, and other organs	32 (3.2)	977 (96.8)

Formatted: Left

	Fish or shellfish.	45 (4.5)	964 (95.5)
**Overall vitamin A rich foods consumptions**		**280 (27.7)**	**729 (72.3)**
Consumed foods rich in iron at any time in 24 hours	Eggs	85 (8.4)	924 (91.6)
	Meat (beef, pork, lamb, chicken)	52 (5.2)	957 (94.8)
	Liver, heart, and other organs	32 (3.2)	977 (96.8)
	Fish or shellfish.	45 (4.5)	964 (95.5)
**Overall iron rich food consumption**		**157 (15.6)**	**852 (84.4)**
Multiple micronutrient powder within seven days		76 (7.5)	933 (92.5)
Iron supplements within seven days		60 (6.0)	949 (94.0)
Vitamin A supplements within six months		476 (47.2)	533 (52.8)
Deworming medication in the six months (n=547)		46 (8.4)	501 (91.6)
**Overall, received at least the minimum recommended micronutrient**		**633 (62.7)**	**376 (37.3)**

Random effects (measures of variation)

There was a significant variation in the intake of micronutrients among children aged 6 to 23 months across the communities (clusters). The intra-cluster correlation coefficient (ICC) in the null model (model 0) for micronutrient intake was 0.273. In other words, 27.3% of the variation in micronutrient intake among children aged 6 to 23 months is due to the differences between regions/clusters (between-cluster variation) (Table 5).

Table 5. Results from a random intercept model (a measure of variation) for micronutrient intake among children aged 6 to 23 months at cluster level by multilevel logistic regression analysis, EDHS 2016.

Formatted: Font: Not Bold

Measure of variations	Model 0 (null model)	Model 1	Model 2	Model 3 (full model)
Variance	3.35	1.49	1.61	1.43
Explained variation (PCV) (%)	Ref.	55	52	57

ICC (%)	27.3	62.2	73.3	79.9
Model fitness				
Deviance (-2*log likelihood)	1271.9	1154.7	1195.0	1135.4
AIC	1275.9	1200.4	1214.5	1193.8

AIC: Akaike's Information Criterion

ICC: Intra-class Correlation Coefficient

PCV: Proportional Change in Variance

Model 0: without independent variables (null model)

Model 1: only individual-level variables

Model 2: only community-level variables

Model 3: individual and community-level variables (full model)

Individual and community-level factors of micronutrient intake status (fixed effects)

In the bivariable analysis, mothers educational status and occupation, sex of household head, household wealth status, age at first birth, ANC and PNC visits, place of delivery, desire for more child, age of the child, currently breastfeeding, history of diarrhea and cough within the last two weeks and current pregnancy status was the individual level candidate variables. Whereas residence, region, community-level poverty and community level media exposure were the community level candidate variables.

In the final model (model 3), after adjusting for individual and community level factors, women's occupational status, the current age of the child, antenatal visits for current pregnancy, residence and region were significantly associated with the micronutrient intake status among children aged 6 to 23 months.

Accordingly, children whose mothers/caregivers with an agricultural occupation were 2.22 times more likely to receive the recommended micronutrient compared to those children whose mothers/caregivers with no work (AOR: 2.22, 95% CI: 1.30-3.80). Children born from mothers who had ANC visits for their recent pregnancy were 1.95 times more likely to receive the recommended micronutrient compared to those who had not ANC visits (AOR: 1.95, 95% CI:1.37-2.77). Those children aged 13 to 23 months were 1.72 times more likely to receive the recommended micronutrient compared to those aged 6 to 12 months (AOR: 1.72, 95% CI: 1.25-2.36). Those children who reside in the rural communities were 63% less likely to receive the recommended micronutrient compared to their counterparts (AOR: 0.37, 95% CI: 0.14-0.88). Children who live in the Benishangul and Gambella region were 2.52 (AOR: 2.52, 95% CI: 1.29-4.93) and 1.87 (AOR: 1.87, 95% CI: 1.03-3.38) times more likely to take the recommended micronutrient compared to those children who live in the Afar region, respectively (Table 6).

Table 6. Multilevel logistic regression analysis of factors associated with micronutrient intake status among children aged 6-23 months in the emerging regions of Ethiopia, EDHS 2016 (n=1009).

Formatted: Font: Not Bold

Variables	Received at least one of the recommended ~~micronutrient~~micronutrients		COR (95%CI)	Model 0 (ICC: 27.3%)	Model 1 AOR (95% CI)	Model 2 AOR (95%CI)	Model 3 AOR (95%CI)
	Yes n (%)	No n (%)					
Individual-level characteristics							
Mothers educational status							
No education	409 (57.2)	306 (42.8)	1		1		1
Primary	150 (75.4)	49 (24.6)	1.89 (1.24-2.88)		1.44 (0.94-2.22)		1.19 (0.76-1.87)
Secondary	52 (77.6)	15 (22.4)	2.38 (1.19-4.76)		1.52 (0.75-3.10)		1.20 (0.57-2.54)
Higher	22 (78.6)	6 (21.4)	2.47 (0.84-7.20)		1.17 (0.38-3.56)		0.83 (0.25-2.75)
Household head							
Male	435 (64.5)	239 (35.5)	1		1		1
Female	198 (59.1)	137 (40.9)	0.77 (0.55-1.07)		0.81 (0.58-1.12)		0.86 (0.61-1.19)
Mothers' occupation							
No work	371 (55.3)	300 (44.7)	1		1		1
Professional	57 (71.3)	23 (28.7)	1.78 (0.98-3.21)		1.38 (0.75-2.54)		1.38 (0.75-2.55)
Agricultural	154 (81.5)	35 (18.5)	3.34 (2.06-5.43)		3.04 (1.89-4.88)		2.22 (1.30-3.80) *
Others	51 (73.9)	18 (26.1)	2.03 (1.07-3.86)		1.51 (0.78-2.93)		1.34 (0.68-2.62)
Wealth status							
Poor	418 (57.3)	312 (42.7)	1		1		1
Middle	61 (78.2)	17 (21.8)	2.33 (1.23-4.40)		1.43 (0.75-2.74)		1.17 (0.46-3.00)
Rich	154 (76.6)	47 (23.4)	2.12 (1.34-3.59)		1.40 (0.86-2.26)		0.54 (0.20-1.47)
Age at first birth in years							
Less than 18	248 (61.7)	154 (38.3)	1		1		1
18-24	347 (63.1)	203 (36.9)	1.23 (0.89-1.69)		1.18 (0.86-1.62)		1.18 (0.86-1.63)

25+	38 (66.7)	19 (33.3)	1.54 (0.76-3.13)		1.43 (0.72-2.87)		1.40 (0.69-2.86)
ANC visit							
No	214 (48.6)	226 (51.4)	1		1		1
Yes	419 (73.6)	150 (26.3)	2.78 (2.01-3.84)		2.03 (1.43-2.86)		1.95 (1.37-2.77) *
Place of delivery							
Home	429 (57.6)	316 (42.4)	1		1		1
Health facility	204 (77.3)	60 (22.7)	2.39 (1.61-3.54)		1.31 (0.85-2.02)		1.20 (0.77-1.89)
PNC visit							
Yes	62 (84.9)	11 (15.1)	1		1		1
No	571 (61.0)	365 (39.0)	0.30 (0.14-0.64)		0.54 (0.25-1.13)		0.54 (0.25-1.17)
Desire more child							
Wants	478 (59.9)	320 (40.1)	1		1		1
Undecided	27 (62.8)	16 (37.2)	1.24 (0.59-2.59)		1.05 (0.50-2.19)		1.15 (0.55-2.39)
Wants no more	128 (76.2)	40 (23.8)	1.94 (1.24-3.05)		1.87 (1.19-2.94)		1.54 (0.96-2.46)
Child currently breastfeed							
No	111 (56.1)	87 (43.9)	1		1		1
Yes	522 (64.4)	289 (35.6)	1.36 (0.93-1.98)		1.17 (0.76-1.82)		1.15 (0.74-1.80)
Currently pregnant							
No	585 (63.7)	334 (36.3)	1		1		1
Yes	48 (53.3)	42 (46.7)	0.62 (0.36-1.05)		0.73 (0.40-1.32)		0.79 (0.43-1.44)
Age of child in months							
6-12	259 (56.1)	203 (43.9)	1		1		1
13-23	374 (68.4)	173 (31.6)	1.74 (1.28-2.35)		1.79 (1.31-2.45)		1.72 (1.25-2.36) *
Diarrhoea in the last two weeks							
No	522 (61.5)	327 (38.5)	1		1		1
Yes	111 (69.4)	49 (30.6)	1.53 (1.00-2.35)		1.27 (0.81-1.99)		1.28 (0.82-2.00)
Cough in the last two weeks							
No	558 (64.0)	314 (36.0)	1		1		1
Yes	75 (54.7)	62 (42.3)	0.73 (0.47-1.12)		0.59 (0.38-0.92)		0.65 (0.41-1.02)
Community-level characteristics							
Residence							

Urban	126 (77.3)	37 (22.7)	1			1	1
Rural	507 (60.0)	339 (40.0)	0.42 (0.24-0.75)			0.42 (0.24-0.72)	0.35 (0.14-0.88) *
Region							
Afar	127 (50.0)	127 (50.0)	1			1	1
Somali	182 (52.6)	164 (47.4)	1.11 (0.69-1.81)			1.02 (0.65-1.59)	1.13 (0.71-1.76)
Benishangul	187 (83.4)	37 (16.6)	6.44 (3.54-11.7)			5.27 (2.87-9.66)	2.52 (1.29-4.93) *
Gambella	137 (74.0)	48 (26.0)	3.81 (2.12-6.87)			2.87 (1.63-5.04)	1.87 (1.03-3.38) *
Community-level poverty							
Poorest	350 (55.0)	287 (45.0)	1			1	1
Poorer	120 (74.0)	42 (26.0)	1.89 (1.19-3.00)			1.20 (0.74-1.94)	1.07 (0.64-1.77)
Middle	67 (71.3)	27 (28.7)	1.88 (1.08-3.26)			1.15 (0.64-2.04)	0.78 (0.35-1.73)
Richer	62 (83.8)	12 (16.2)	3.65 (1.77-7.52)			2.04 (0.98-4.26)	2.34 (0.76-7.13)
Richest	34 (81.0)	8 (19.0)	3.43 (1.42-8.31)			1.95 (0.79-4.80)	2.59 (0.67-9.93)
Community-level media exposure							
No	593 (61.8)	366 (38.2)	1			1	1
Yes	40 (80.0)	10 (20.0)	1.89 (0.83-4.30)			1.03 (0.44-2.36)	1.07 (0.44-2.61)

~~*statistically~~*Statistically significant at p-value <0.05 at model 3

ANC: Antenatal Care,

AOR: Adjusted Odds Ratio,

COR: Crude Odds Ratio,

ICC: Intra-class Correlation Coefficient,

PNC: Postnatal Care

Discussion

The ~~overall status~~study showed that at least one of the recommended micronutrient intake among children aged 6-23 months in the emerging regions of Ethiopia was found to be 62.7%. The individual-level factors such as the age of the child, mother's occupation, and current ANC follow up have been significantly associated with the intake of micronutrients. From the community-level factors, ~~the~~ residence and region were significantly associated with micronutrient intake.

Micronutrient is available in foods and can also be provided through a direct supplement. In this study, we assessed the level of vitamin A and iron intake. The result showed that around 28 and 15.~~56~~6% of children consume foods rich in vitamin A and iron, respectively. From EDHS 2016 report, consumption of foods rich in vitamin A and iron were 38 and 22%, correspondingly, and lowest intake was observed in Afar [49], which is comparable with the current finding. ~~In addition~~Besides, almost half of the children (47.18%) get vitamin A supplements~~,~~ and as few as 6% of them get iron supplements. The previous EDHS (2011) finding showed that vitamin A supplement, in the four regions, was 43.17%, which is lower than the current ~~finding~~result [36]. This might be due to the emphasis given by the government to achieve the first phase of the nutritional program.

Moreover, the national micronutrient survey (2016) revealed that the national coverage of vitamin supplements was 63% [50], which is higher than the current finding. However, the report showed that sub-clinical vitamin A deficiency was 14%. Vitamin A supplement in Nigeria was 45~~%,~~% which is comparable with the current finding [51]. However, our study differs from the finding in India (30.4%) [52].

The finding showed that at least one of the ~~overall~~recommended micronutrient intake status was 62.7%, which is lower than that of the national target of over 90% [53]. Besides, there was

Formatted: Default Paragraph Font, Font: +Body (Calibri), 11 pt, Font color: Auto

a considerable difference between the regions. The National Nutritional Program end-line survey report was also comparable with the current findings [54]. Benishangul and Gambella regions where agriculture, a source of legume and cereal plants, is very common, had a higher intake. However, regions of Afar and Somalia are inhabited by pastoralist people with a scattered settlement where fresh fruit and vegetables are not readily available. Besides, healthcare facilities may also be inaccessible and under-staffed. A study from the pastoral community showed that the majority source of food was dairy products [55].

This study identified that micronutrient intake among children whose mothers worked in agriculture was higher compared with children whose mothers did not have work. Mothers who work in agriculture might have better access to diversified agricultural products and animal products that are rich sources for vitamins and minerals. Moreover, participating in work may expose mothers with peers and friends that can serve as a source of information related to micronutrient intake and benefits. This study also showed that agrarian dominants were more likely to consume diversified food, which can be used as a proxy for adequate micronutrient density of foods [56]. Previous studies are also consistent with the current finding [36, 57].

The odds of micronutrient intake for children age 13-23 were higher compared to children age between 6-12 months. The possible explanation could be that the age groups have better nutritional diversity. In the EDHS 2016, for example, children over 12 months old were more likely to obtain diversified food, ~~a possible source of~~which implies that they can got more micronutrients~~.~~ [56, 58]. On the other way, the late introduction of complementary feeding might have resulted in consuming a limited variety of food such as only milk or cereal products. Moreover, mothers' perception of low ability of children intestine and traditional beliefs might contribute to low consumption of diversified food in those children (6-12 months). Also, even if vitamin A supplement is effective for 6-11 months, especially when used with the vaccine against measles, vitamin A supplementation can be reduced by dropping out of this age group~~,~~

Formatted: Font: Times New Roman, 12 pt, Font color: Text 1

and certain children (6-12 months) were not eligible to receive vitamin A supplement during the survey. Studies from Nigeria are also comparable to the current finding [51, 57].

Formatted: English (United Kingdom)

In this study, higher odds of micronutrient intake were observed among children whose mother had ANC follow-ups compared to those children whose mother did not have ANC follow-ups. This finding was in line with these of previous studies conducted in Ethiopia [36]. ~~A plausible~~The possible explanation might be mothers who had ANC follow-up may have a chance of getting information, education, knowledge and also counselling services on foods rich in micronutrients and ~~the~~its availability ~~of supplements~~ from health ~~professionals.~~professionals' talks. Furthermore, they might ~~be~~ able to learn about the value of iron ~~by taking iron~~intake that supplements during their ANC follow-up. Moreover, a systematic review by Temesgen et al. suggests that children whose mothers have ANC follow-up have a higher probability than their counterparts to eat diversified food [59].

The odds of micronutrient intake among children who reside in rural communities were lower compared to their counterparts. This is supported by a systematic study in Ethiopia, which reported that urban residents had higher odds of micronutrient intake than rural residents [59]. However, few studies' findings [51, 52, 60] contradict with the current study. The potential explanation might be food fortification~~,~~ and supplementation focused more ~~for~~on rural than urban through community-based maternal and child health outreach programs.

Our finding shows that micronutrient intake among children who live in Benishangul and Gambella regions was higher compared to those who live in the Afar region. This can be explained by the fact that, compared to the two regions, the economic activities of the Afar and Somalia regions are mostly dominated by cattle breeding and pastoral lifestyles, and agriculture is common in Benishangul and Gambella. Besides, since the latter two regions have dense forests and water reservoirs, they could get wild fruit and fish, which are a good source of micronutrients. Previous studies showed that in the pastoral community, vitamin A rich foods

were scarce, and meat and egg consumption were low [55]. Natural forest and semi-natural forests were positively associated with a larger number of nutritionally important food groups [61]. A study from the recent EDHS (2016) showed that children of the agrarian community were more likely to consume diversified food than that of the pastoral community.

Strength and limitations of the study

The main strengths of the study are its representativeness, large sample size, and the availability of individual and community-level factors that demonstrate the actual micronutrient intake status of children in the emerging regions. Besides, this study used a multilevel-modelling technique to identify a more valid result that takes the hierarchical nature of the survey data into account. Furthermore, the DHS surveys have similar designs with similar variables in a different setting; the result may, therefore, be applied to other similar settings.

The study ~~attempted~~tried to assess the intake status of micronutrient using the national data and discuss this with other cross-sectional studies; focus on nutritional studies that can under or overestimates our findings. Moreover, the study used the national micronutrient recommendation for the assessment of the outcome variable which might not significantly measure the adequacy of intake. Furthermore, the mothers might have experienced recall bias, particularly regarding vitamin A supplementation and deworming for their child in the last six months prior to the survey, for instance. Compared to other studies, however, our study assessed later events that preceded the study by only six months which might decrease the recall bias.

~~Conclusion~~

Conclusions

In conclusion, the overall intake of micronutrients in this study was below the national recommendation. Besides, there ~~were~~was a lower intake of micronutrients in the two pastorally dominated regions (Afar and Somalia). Individual-level characteristics such as mothers' occupation and age of a child and community-level characteristics ANC for index child, residence, and region were significantly associated with the micronutrient intake status.

Therefore, for the improvement of micronutrient intake, the regional and zonal health departments and the health facilities needs to cooperate with state governments across the regions, continuously increase the amount of vitamin A and the iron supplementation, encourage the use of Vitamin A and iron-rich foods, and reinforce community-based outreach programs for maternal health and child health programs.

List of Abbreviations

ANC: Antenatal Care, AOR: Adjusted Odds Ratio, CI: Confidence Interval, COR: Crude Odds Ratio, CSA: Central Statistical Agency, EA: Enumeration Areas, EDHS: Ethiopian Demographic and Health Survey, FAO: Food and Agriculture Organizations, FMoH: Federal Ministry of Health, ICC: Intra-class Correlation Coefficients, MN: Micronutrients, PNC: Postnatal Care, and WHO: World Health Organization

Acknowledgments

We are very thankful to MEASURE DHS for permission to use the EDHS 2016 survey data sets.

References

1. Von Grebmer K, Saltzman A, Birol E, Wiesman D, Prasai N, Yin S, et al. Synopsis: 2014 Global Hunger Index: The Challenge of Hidden Hunger: Intl Food Policy Res Inst; 2014.

2. Tzioumis E, Kay MC, Bentley ME, Adair LS. Prevalence and trends in the childhood dual burden of malnutrition in low-and middle-income countries, 1990–2012. Public health nutrition. 2016;19(8):1375-88.

3. Harika R, Faber M, Samuel F, Kimiywe J, Mulugeta A, Eilander A. Micronutrient status and dietary intake of iron, vitamin A, iodine, folate and zinc in women of reproductive age and pregnant women in Ethiopia, Kenya, Nigeria and South Africa: a systematic review of data from 2005 to 2015. Nutrients. 2017;9(10):1096.

4. Azadbakht L, Esmaillzadeh A. Macro and Micro-Nutrients Intake, Food Groups Consumption and Dietary Habits among Female Students in Isfahan University of Medical Sciences. Iranian Red Crescent medical journal. 2012;14(4):204-9. Epub 2012/07/04. PubMed PMID: 22754682; PubMed Central PMCID: PMCPMC3385798.

5. Bush LA, Hutchinson J, Hooson J, Warthon-Medina M, Hancock N, Greathead K, et al. Measuring energy, macro and micronutrient intake in UK children and adolescents: a comparison of validated dietary assessment tools. BMC Nutrition. 2019;5(1):53. doi: 10.1186/s40795-019-0312-9.

6. Herrador Z, Sordo L, Gadisa E, Buño A, Gómez-Rioja R, Iturzaeta JM, et al. Micronutrient deficiencies and related factors in school-aged children in Ethiopia: a cross-sectional study in Libo Kemkem and Fogera districts, Amhara Regional State. PLoS One. 2014;9(12):e112858. Epub 2014/12/30. doi: 10.1371/journal.pone.0112858. PubMed PMID: 25546056; PubMed Central PMCID: PMCPMC4278675.

7. Sharma P, Dwivedi S, Singh D. Global poverty, hunger, and malnutrition: a situational analysis. Biofortification of food crops: Springer; 2016. p. 19-30.

8. Tariku A, Bikis GA, Woldie H, Wassie MM, Worku AG. Child wasting is a severe public health problem in the predominantly rural population of Ethiopia: A community based cross–sectional study. Archives of Public Health. 2017;75(1):26.

9. Bharaniidharan J, Reshmi S. Review on Malnutrition: Impact and Prevention. International Journal. 2019;7(3):240-3.

10. Ritchie H, Roser M. Micronutrient deficiency. Our World in data. 2017.

11. Ames BN. Low micronutrient intake may accelerate the degenerative diseases of aging through allocation of scarce micronutrients by triage. Proceedings of the National Academy of Sciences of the United States of America. 2006;103(47):17589-94. Epub 2006/11/15. doi: 10.1073/pnas.0608757103. PubMed PMID: 17101959.

12. Schneider J, Fumeaux CJF, Duerden EG, Guo T, Foong J, Graz MB, et al. Nutrient intake in the first two weeks of life and brain growth in preterm neonates. Pediatrics. 2018;141(3):e20172169.

13. Velasco I, Bath SC, Rayman MP. Iodine as essential nutrient during the first 1000 days of life. Nutrients. 2018;10(3):290.

14. Keeley B, Little C, Zuehlke E. The State of the World's Children 2019: Children, Food and Nutrition--Growing Well in a Changing World. UNICEF. 2019.

15. Ahmed F, Prendiville N, Narayan A. Micronutrient deficiencies among children and women in Bangladesh: progress and challenges. Journal of nutritional science. 2016;5:e46. Epub 2017/06/18. doi: 10.1017/jns.2016.39. PubMed PMID: 28620473; PubMed Central PMCID: PMCPMC5465809.

16. Nunn RL, Kehoe SH, Chopra H, Sahariah SA, Gandhi M, Di Gravio C, et al. Dietary micronutrient intakes among women of reproductive age in Mumbai slums. European journal of clinical nutrition. 2019;73(11):1536-45. doi: 10.1038/s41430-019-0429-6.

17. Kennedy GL, Pedro MR, Seghieri C, Nantel G, Brouwer I. Dietary Diversity Score Is a Useful Indicator of Micronutrient Intake in Non-Breast-Feeding Filipino Children. The Journal of nutrition. 2007;137(2):472-7. doi: 10.1093/jn/137.2.472.

18. Newmann SJ, Mishra K, Onono M, Bukusi EA, Cohen CR, Gage O, et al. Providers' Perspectives on Provision of Family Planning to HIV-Positive Individuals in HIV Care in Nyanza Province, Kenya. AIDS Res Treat. 2013;2013:915923. doi: 10.1155/2013/915923. PubMed PMID: 23738058; PubMed Central PMCID: PMCPMC3659431.

19. Lander RL, Enkhjargal T, Batjargal J, Bailey KB, Diouf S, Green TJ, et al. Multiple micronutrient deficiencies persist during early childhood in Mongolia. Asia Pacific journal of clinical nutrition. 2008;17(3):429-40. Epub 2008/09/27. PubMed PMID: 18818163.

20. Sheehy T, Carey E, Sharma S, Biadgilign S. Trends in energy and nutrient supply in Ethiopia: a perspective from FAO food balance sheets. Nutrition journal. 2019;18(1):46. doi: 10.1186/s12937-019-0471-1.

21. Central Statistical Agency Addis Ababa E. Ethiopia Demographic and Health Survey 2016 Addis Ababa, Ethiopia 2016.

22. Dangura D, Gebremedhin S. Dietary diversity and associated factors among children 6-23 months of age in Gorche district, Southern Ethiopia: Cross-sectional study. BMC pediatrics. 2017;17(1):6.

23. Birhanu M, Abegaz T, Fikre R. Magnitude and Factors Associated with Optimal Complementary Feeding Practices among Children Aged 6-23 Months in Bensa District, Sidama Zone, South Ethiopia. Ethiopian journal of health sciences. 2019;29(2).

24. Forsido SF, Kiyak N, Belachew T, Hensel O. Complementary feeding practices, dietary diversity, and nutrient composition of complementary foods of children 6–24 months old in Jimma Zone, Southwest Ethiopia. Journal of Health, Population and Nutrition. 2019;38(1):14.

25. Mulat E, Alem G, Woyraw W, Temesgen H. Uptake of minimum acceptable diet among children aged 6–23 months in orthodox religion followers during fasting season in rural area, DEMBECHA, north West Ethiopia. BMC Nutrition. 2019;5(1):18.

26. Sagaro GG AM. Dietary Diversity and Associated Factors Among Infants and Young Children in Wolaita Zone, Southern Ethiopia. Science Journal of Clinical Medicine. July 2017;6, , (4,): 53-9.

27. Belay A, Joy EJ, Chagumaira C, Zerfu D, Ander EL, Young SD, et al. Selenium Deficiency Is Widespread and Spatially Dependent in Ethiopia. Nutrients. 2020;12(6):1565.

28. Herrador Z, Sordo L, Gadisa E, Buño A, Gómez-Rioja R, Iturzaeta J, et al. Micronutrient Deficiencies and Related Factors in School-Aged Children in Ethiopia: A Cross-Sectional Study in Libo Kemkem and Fogera Districts, Amhara Regional State. PloS one. 2014;9:e112858. doi: 10.1371/journal.pone.0112858.

29. Shivakoti R, Christian P, Yang WT, Gupte N, Mwelase N, Kanyama C, et al. Prevalence and risk factors of micronutrient deficiencies pre- and post-antiretroviral therapy (ART) among a diverse multicountry cohort of HIV-infected adults. Clinical nutrition. 2016;35(1):183-9. Epub 2015/02/24. doi: 10.1016/j.clnu.2015.02.002. PubMed PMID: 25703452; PubMed Central PMCID: PMCPMC4531105.

30. Serra-Majem L, Ribas L, Pérez-Rodrigo C, García-Closas R, Peña-Quintana L, Aranceta J. Determinants of nutrient intake among children and adolescents: results from the enKid Study. Annals of Nutrition and Metabolism. 2002;46(Suppl. 1):31-8.

31. Cheng Y, Dibley MJ, Zhang X, Zeng L, Yan H. Assessment of dietary intake among pregnant women in a rural area of western China. BMC Public Health. 2009;9(1):222. doi: 10.1186/1471-2458-9-222.

32. Cetin I, Bühling K, Demir C, Kortam A, Prescott SL, Yamashiro Y, et al. Impact of Micronutrient Status during Pregnancy on Early Nutrition Programming. Annals of Nutrition and Metabolism. 2019;74(4):269-78. doi: 10.1159/000499698.

33. Birhanu M, Abegaz T, Fikre R. Magnitude and Factors Associated with Optimal Complementary Feeding Practices among Children Aged 6-23 Months in Bensa District,

Sidama Zone, South Ethiopia. Ethiop J Health Sci. 2019;29(2):153-64. Epub 2019/04/24. doi: 10.4314/ejhs.v29i2.2. PubMed PMID: 31011263; PubMed Central PMCID: PMCPMC6460456.

34. Iannotti L. Dietary Intakes and Microntrient Adequacy Related to the Changing Livelihoods of Two Pastoralist Communities in Samburu, Kenya. Current Anthropology. 2014;55:475-82. doi: 10.1086/677107.

35. Bach A, Gregor E, Sridhar S, Fekadu H, Fawzi W. Multisectoral Integration of Nutrition, Health, and Agriculture: Implementation Lessons From Ethiopia. Food and nutrition bulletin. 2020:0379572119895097.

36. Haile D, Biadgilign S, Azage M. Differentials in vitamin A supplementation among preschool-aged children in Ethiopia: evidence from the 2011 Ethiopian Demographic and Health Survey. Public health. 2015;129(6):748-54.

37. Gebre-Egziabhere T. Emerging Regions in Ethiopia: Are they catching up with the rest of Ethiopia? Eastern Africa Social Science Research Review. 2018;34(1):1-36.

38. Stark J, Terasawa K, Ejigu M. Climate change and conflict in pastoralist regions of Ethiopia: mounting challenges, emerging responses. Conflict Management and Mitigation (CMM) Discussion Paper. 2011;(4).

39. Central Statistical Agency (CSA) [Ethiopia] and ICF. Ethiopia Demographic and Health Survey 2016. Addis Ababa, Ethiopia, and Rockville, Maryland, USA: CSA and ICF. 2016. https://dhsprogram.com. 2016.

40. WHO. Use of multiple micronutrient powders for point‐ of‐ use fortification of foods consumed by infants and young children aged 6–23 months and children aged 2–12 years guideline2016. 60. https://www.who.int/nutrition/publications/micronutrients/guidelines/mmpowders-infant6to23mons-children2to12yrs/en/ p.

41. Dary O, Hurrell R. Guidelines on food fortification with micronutrients. World Health Organization, Food and Agricultural Organization of the United Nations: Geneva, Switzerland. 2006.

42. FAO, WHO. Human vitamin and mineral requirements. Report of a joint FAO/WHO expert consultation, Bangkok, Thailand. Food and Nutrition Division, FAO, Rome. 2001:235-47.

43. WHO. Protein and amino acid requirements in human nutrition: report of a joint FAO/WHO/UNU expert consultation. Geneva; 2007. WHO Technical Report Series. 935.

44. Organization WH. WHO guideline: use of multiple micronutrient powders for point-of-use fortification of foods consumed by infants and young children aged 6–23 months and children aged 2–12 years: World Health Organization; 2016. 60 p.

45. Russell R, Beard JL, Cousins RJ, Dunn JT, Ferland G, Hambidge K, et al. Dietary reference intakes for vitamin A, vitamin K, arsenic, boron, chromium, copper, iodine, iron, manganese, molybdenum, nickel, silicon, vanadium, and zinc. A Report of the Panel on Micronutrients, Subcommittees on Upper Reference Levels of Nutrients and of Interpretation and Uses of Dietary Reference Intakes, and the Standing Committee on the Scientific Evaluation of Dietary Reference Intakes Food and Nutrition Board Institute of Medicine. 2001.

46. Tabacchi G, Wijnhoven TM, Branca F, Roman-Vinas B, Ribas-Barba L, Ngo J, et al. How is the adequacy of micronutrient intake assessed across Europe? A systematic literature review. The British journal of nutrition. 2009;101 Suppl 2:S29-36. Epub 2009/07/15. doi: 10.1017/s0007114509990560. PubMed PMID: 19594962.

47. Croft TN, Aileen M. J. Marshall, Courtney K. Allen, et al.,. Guide to DHS Statistics. Rockville, Maryland, USA: ICF. 2018.

48. Hox JJ, Moerbeek M, Van de Schoot R. Multilevel analysis: Techniques and applications. Third ed: Routledge; 2010. 364. https://b-ok.cc/book/3695027/0a101f p.

49. Central Statistical Agency (CSA) [Ethiopia] and ICF. 2016. Ethiopia Demographic and Health Survey 2016 2016. Addis Ababa, Ethiopia, and Rockville, Maryland, USA: CSA and ICF,].

50. The Ethiopian Public Health Institute. Ethiopian National Micronutrent Survey Report 2016. Available from: https://www.ephi.gov.et/images/pictures/download2009/National_MNS_report.pdf.

51. Aghaji AE, Duke R, Aghaji UC. Inequitable coverage of vitamin A supplementation in Nigeria and implications for childhood blindness. BMC public health. 2019;19(1):282.

52. Agrawal S, Agrawal P. Vitamin A supplementation among children in India: Does their socioeconomic status and the economic and social development status of their state of residence make a difference? International journal of medicine and public health. 2013;3(1):48.

53. The Federal Democratic Republic of Ethiopia. National Nutrition Programme: June 2013–June 2015, MOH, Addis Ababa, Ethiopia 2012. Available from: https://extranet.who.int/nutrition/gina/sites/default/files/ETH%202013%20National%20Nutrition%20Programme.pdf.

54. Ayana G, Hailu A, Tessema M, Belay A, Zerfu D, Bekele T, et al. Ethiopian National Nutrition Program End-Line Survey2015.

55. Mengistu G, Moges T, Samuel A, Baye K. Energy and nutrient intake of infants and young children in pastoralist communities of Ethiopia. Nutrition. 2017;41:1-6.

56. Tassew AA, Tekle DY, Belachew AB, Adhena BM. Factors affecting feeding 6–23 months age children according to minimum acceptable diet in Ethiopia: A multilevel analysis of the Ethiopian Demographic Health Survey. PloS one. 2019;14(2).

57. Aremu O, Lawoko S, Dalal K. Childhood vitamin A capsule supplementation coverage in Nigeria: a multilevel analysis of geographic and socioeconomic inequities. The Scientific World Journal. 2010;10:1901-14.

58. Aemro M, Mesele M, Birhanu Z, Atenafu A. Dietary diversity and meal frequency practices among infant and young children aged 6–23 months in Ethiopia: a secondary analysis of Ethiopian demographic and health survey 2011. Journal of nutrition and metabolism. 2013;2013.

59. Temesgen H, Negesse A, Woyraw W, Mekonnen N. Dietary diversity feeding practice and its associated factors among children age 6–23 months in Ethiopia from 2011 up to 2018: a systematic review and meta-analysis. Italian journal of pediatrics. 2018;44(1):1-10.

60. Immurana M, Arabi U. Socio-economic covariates of micronutrients supplementation and deworming among children in Ghana. J Behav Health. 2016;4:154-61.

61. Ickowitz A, Rowland D, Powell B, Salim MA, Sunderland T. Forests, trees, and micronutrient-rich food consumption in Indonesia. PloS one. 2016;11(5).

).

Supporting information files

Data file “STATA Data__Micronutrient intake status among children aged 6-23 months_EDHS 2016”

Formatted: Font: 12 pt
