# Supplementary material for: Micronutrient intake status and associated factors among children aged 6–23 months in the emerging regions of Ethiopia: A multilevel analysis of the 2016 Ethiopia demographic and health survey
Source: PLoS One. 2021 Oct 22;16(10):e0258954. doi: 10.1371/journal.pone.0258954 (PMC8535338; doi:10.1371/journal.pone.0258954)
Supplement: Attachment — Submitted filename: Point-by-point response to reviewers comments.docx [file pone.0258954.s001.docx]

**Response to Reviewers’**

**Title: “**Micronutrient intake status and associated factors among children aged 6-23 months in the emerging regions of Ethiopia: a multilevel analysis of the 2016 Ethiopian demographic and health survey." [Manuscript ID: PONE-D-20-13718]

**Subject: Submission of a revised manuscript for publication**

Dear Editor,

Greetings!

Firstly, we would like to appreciate and thank the academic editor and reviewers for investing their time and energy to review and make comments. It is with great pleasure to receive the invaluable and constructive comments which improves our manuscript.

We accepted and tried to incorporate all of the comments provided. Thus, the comments are attached here below with their point-by-point responses. In addition, the detailed changes made are highlighted in the “revised manuscript with track changes” to easily identify the changes/improvements and also the clean copy of the revised manuscript is prepared.

Finally, we kindly request you to review our revised manuscript which is presented in a table form below.

**Point-by-point responses for the editor’s and reviewers’ comments**

| **No** | **Comments** | **Authors’ response** |
| --- | --- | --- |
| ***-***  1. | Journal requirements:  When submitting your revision, we need you to address these additional requirements:  Please ensure that your manuscript meets PLOS ONE's style requirements, including those for file naming. The PLOS ONE style templates can be found at <http://www.plosone.org/attachments/PLOSOne_formatting_sample_main_body.pdf> and <http://www.plosone.org/attachments/PLOSOne_formatting_sample_title_authors_affiliations.pdf> | Thank you so much for your comments. The comment is accepted and corrected in the style of the PLOS ONE journal. Please refer to the clean version of the revised manuscript. |
| **2.** | Please modify the title to ensure that it is meeting PLOS’ guidelines (<https://journals.plos.org/plosone/s/submission-guidelines#loc-title>) . In particular, the title should be "specific, descriptive, concise, and comprehensible to readers outside the field" and in this case we have concerns that the title contains a causal statement not completely supported by the study. | Again, thank you very much for your very kindly and careful review. We had gone throughout the linked document and we have modified the title “Micronutrient intake status and associated factors among children aged 6-23 months in the emerging regions of Ethiopia: a multilevel analysis of the 2016 Ethiopian demographic and health survey” to be more specific, descriptive, concise and comprehensive with the findings. Please see the first page of the clean version of the revised manuscript. |
|  | **Reviewer #1:** | **Authors’ response** |
| 1) | I do not find there to be a strong rationale in the paper for why only data from four regions is analysed, when (presumably) the EDHS has data available from other regions as well. | Dear reviewer, thank you for your constructive comments. We revised the introduction and set out the rationale of the study as per the comments provided; please see the clean version of the revised manuscript on page 5, lines 72-81. |
| 2) | Dependent variable. The individual questions are well explained but I do not fully understand how you combined these into the outcome variable. In the paper you refer both to micronutrient "intake status", which could be categorical, but also "level of intake" which sounds continuous; however, my understanding is your outcome variable is binary, in which case "minimum intake" or similar would be most appropriate. Furthermore, was a positive answer to any of the six questions sufficient for the outcome to be coded as a "yes"? On a conceptual level, I find it difficult to understand why this makes sense if the child has received deworming medication but answers to the five other questions are negative. | Dear reviewer, thank you very much for your comments and insights. The dependent variable of the study was categorical; It was dichotomized in yes and no category. when one chid got at least one of the minimum recommended micronutrients, they were considered us “got the recommended micronutrient”; otherwise it was considered as that “the recommended micronutrient is not received”. We used the classification according to the national micronutrient recommendations, and if the child received at least one of the minimum recommended micronutrients, the national recommendation stated that they were considered “received”; otherwise “not received”. Such recommendation might not give confidence to say the micronutrient intake status is sufficient, so, this might be one of the limitations of the study mentioned on the strength and limitation section (page 26, lines 404-406).  Furthermore, for the deworming medication, it was not considered the medication prescribed for treatment of disease/illness; rather the national micronutrient recommendation has included it, for any heathy child, it should be supplement alongside with the vitamin A and others. So, we have included the deworming medication which was provided as a supplement rather than for those prescribed for the treatment of illness/disease. Please refer to the variables' measurement section in the clean version of the revised manuscript on pages 8-11, lines 132-183. |
| 3) | Sampling procedures. Please use the term "community" in this section, since this is used frequently elsewhere in the paper. Is EA the community level? | Dear reviewer, thank you for your concerns. In the EDHS, enumeration areas were the clusters with a specified household numbers; thus, instead of clusters we used as EA. Furthermore, EA are not the community level rather they are clusters created in the Ethiopian demographic and health survey for sampling purposes. So, we kindly invite you to see the sampling procedures as per those perspectives, on pages 6-7, lines 95-116 in the revised manuscript. |
| 4) | Rows 123-125: Please clarify whether 1009 was the total number of eligible respondents available in the EDHS (from the four regions), or whether you have made some selection. | Dear reviewer, we are thankful for your positive comments to improve the manuscript. In the first step, a total of 1130 participants from the four regions were available as a source. Then, using a selection criterion; alive, first index to birth history and live with their mothers’/caregivers, we have included a total of 1009 eligible children with their mothers/caregivers in the final analysis. we put this in figure 1; please see the clean version of the revised manuscript on page 7, lines 117-124. |
| 5) | The description of multilevel modelling is very nice and while I am not overly familiar, I believe there is a mistake on row 200, where the reference should be to (beta0+Uj) and beta2. | Dear reviewer, we are very much thankful for your observation and comments. We have addressed the issue; please see the clean version of the revised manuscript on page 11, line 203. |
| 6) | The large number of variables describing obstetric history is surprising, given the outcome variable. No rationale is provided in the paper either, and very few end up being included in the final model. | Dear reviewer, thank you for your critical insights and suggestions. In our assumptions, the micronutrient intake status of children can be affected by the maternal obstetric history as we tried to show in the introduction section (page 5, lines 69-71). Moreover, we have revised the obstetric history in the result section and removed few irrelevant variables form the descriptive; please see the clean version of the revised manuscript on page 13, lines 237-243. |
| 7) | Discussion. You mention there is a national target, however, I cannot find a mention of what the target actual is/was. | Thank you for your comments. We mentioned the national targets; please see the clean version on page 23, line 345 of the revised manuscript. Besides, we included that in the introduction section (page 5, lines 65-66). |
| 8) | Strengths and weaknesses. Given the focus on four regions, I am not convinced by your argument that the results are nationally representative, nor that the use of standardised DHS questions makes findings generalisable to other settings. Instead, it would be relevant to discuss strengths and weaknesses of using EDHS as a data source, compared to alternatives e.g. cross-sectional survey focused on nutrition, to answer the research question. | Dear reviewer, thank you for your detailed evaluation. We have addressed the issue; please see the clean version of the manuscript on page 26, lines 396-410. |
| 9) | Conclusion. You recommend specifically continued investment in scaling up vitamin A supplementation, but I cannot find any support in your results to suggest that this would be more important a focus than say iron supplements. | Dear reviewer, thank you so much. We have revised the conclusion section and the problem has been addressed; please see the clean version of the revised manuscript on pages 26-27, lines 412-421. |
| 10) | Data availability. This needs to be revised to refer readers to Measure DHS. | Thank you for your comments. We have addressed the issue. |
| 11) | There are minor spelling mistakes namely "ricked" (row 60), "antennal" (row 305), "vibrations" (Table 5). In references, WHO shows up as Organization WH. | Dear reviewer, thank you for your insights, we have addressed the issue throughout the manuscript please see the clean version of the revised manuscript. |
|  | **Reviewer 2** |  |
| 1) | Line 166-167: Can you clearly state whether Vitamin A uptake and ANC visits was based on mother’s recall, some form of Health documents (like child health card) or both. What about distance to the health facility? This is an ideal variable when it comes to mother’s visits to the clinics. I advise you adequately described, analyzed and discussed if the data exist. | Dear reviewer, thank you very much for your constructive comments. We have addressed the issue; please see the measurement of variables section, particularly on page 9-10, lines 159-183. |
| 2) | Table 4: Please include confidence intervals for the coverages | Dear reviewer, thank you for your comments. We have included the confidence intervals; please see the clean version of the revised manuscript on page 15-16, lines 260-271. |
| 3) | The sample size is not clear. Can you please clarify whether 1009 was the total number of eligible respondents available from the four regions? Also, you have used same denominators for mothers and children sample characteristics. It is possible that a mother was interviewed for more than one child. Can you please clarify here | Again, thank you very much for your very kindly and careful review. We described the EDHS sample selection in text, and presented it in figure as well. Please see the clean version of the revised manuscript on page 7, lines 117-124, and also presented in figure 1. |
| 4) | Discussion: You have only stated the national target but have not provided or given a reference for that. | Dear reviewer, thank you for your insights. We have addressed the issue; please see the clean version of the revised manuscript on page 23, line 345. |
| 5) | There are a few typos and grammatical error. Please get a native English speaker to review and help correct some of the minor mistakes. | Dear reviewer, thank you so much for your comments and suggestions. The manuscript has been revised by English language expert and grammar and spellings have been improved throughout the manuscript, and we made rewording and rephrasing some parts of the paragraphs of the paper accordingly; please see the clean version of the revised manuscript once again. |
| 6) | Strengths and weaknesses: I find it problematic that you have not used the 6 months recall for Vitamin A. I assume the EDHS used the 6 months recall and so I do not think your argument that the results can be compared to national estimates is right especially given the fact that there is some recall bias here. | Dear reviewer, thank you for your valuable comments. We have revised the strength and weaknesses of our study; please see the clean version of the revised manuscript on page 26, lines 396-410. |
| 7) | Please provide p values for comparism where there are significant differences. | Dear reviewer, thank you for your comments and the comment is admitted. We have used the confidence interval and also the p values for the comparison, but to be consistent in writing/editing of the manuscript we did not mentioned it. So, kindly request you to consider it. |
| 8) | Conclusion: What is the national vitamin A coverage and how does it compare to the findings here? Also, worth citing the national Vitamin A deficiency status from a most recent micronutrient survey. Then you need to make this comparism clear before you can recommend continued investment in scaling up vitamin A supplementation. I also think promoting Vitamin A rich foods is a good strategy worth promoting but that will depend on the status of vitamin A in the country. | Dear reviewer, we are very thankful for your comments and suggestions. We have addressed it as per the comments; please see the clean version of the revised manuscript on pages 26-27, lines 412-421. |

Thank you,

Authors
