# Supplementary material for: Micronutrient intake status and associated factors among children aged 6–23 months in the emerging regions of Ethiopia: A multilevel analysis of the 2016 Ethiopia demographic and health survey
Source: PLoS One. 2021 Oct 22;16(10):e0258954. doi: 10.1371/journal.pone.0258954 (PMC8535338; doi:10.1371/journal.pone.0258954)
Supplement: Attachment — Submitted filename: Response to reviewers.doc [file pone.0258954.s004.doc]

**Response to editor’s and reviewers’ comments**

Response to editor’s comments

**Academic editor (Mary Hamer Hodges, MBBS MRCP DSc)**

- The definition of to recommended MN interventions needs to be clearly introduced

**Authors’ response:** Dear editor, thank you for this constructive comment. We have revised and clearly mentioned the recommended micronutrients which has been implemented in Ethiopia by the nutrition supplementation program in the introduction and method section. Accordingly, the recommended micronutrients for children are foods rich in vitamin A within 24 hours, foods rich in iron within 24 hours, multiple micronutrient powder within seven days, iron supplements within seven days, vitamin A supplements within six months, or deworming medication within six months; kindly check on the background and method section.

- Why have you included de-worming?

**Authors’ response:** Dear editor, we are very thankful for the comments. We included deworming in the analysis because the national micronutrient recommendation for children is also included as one of the components, but we measured it for those children above 24 months, which might not have any significance on the measurement of outcome variable.

- The discussion needs to be better oragnized to address first the findings and then comparisons with other manuscripts on MNs.

**Authors’ response:** Dear editor, thank you for this observation, we have made a revision on the discussion section accordingly.

**Response to reviewers’ comments**

Reviewer #2:

Q1: My previous comments have all been addressed. I think there are a few areas with grammatical and typo errors especially in the discussion which needs attention. I advise the authors to find another native/fluent English language speaker to review this thoroughly.

**Authors’ response:** Dear reviewer, thank you very much for your detail comments and suggestions. The manuscript has been revised by language expert and necessary amendments was made throughout the manuscript; kindly see the clean version of the revised manuscript.

Q2: In the discussion, please provide adequate references where comparism is made to other studies.

**Authors’ response:** Dear reviewer, thank for this helpful feedback. Necessary revision was made on the discussion; please see the clean version of the revised manuscript on pages 24-26, lines 348-418.

**Reviewer #3: Comments severe**

L 2: It is unclear what “emerging” means in this context. Please provide an explanation when this word is first introduced.

**Authors’ response:** Dear reviewer, thank you for your comment and we addressed the issue as per the comments. Administratively, Ethiopia has nine regions and two cities administrative and those nine regions categorized in two; emerging regions (Afar, Somali, Benishangul-Gumuz and Gambela) and developed regions (Amhara, Tigray, Oromia, SNNPR, and Harari). This categorization was based on infrastructure, living style and others criteria which was mentioned in the background and method section of the manuscript; kindly see the definition and characteristics of emerging regions in the introduction section on pages 5-6, lines 82-90 and Methods on page 6, lines 101-108 of the clean version of the revised manuscript.

ABSTRACT

L13: Scarcity of MN is due to poor diets rather than poor health care access, health care access helps mitigate the impact of these poor diets but are not the fundamental cause.

**Authors’ response:** Dear reviewer, thank you for your constructive comment and the issue is addressed; kindly see on page 2, lines 12-13.

L15: children 6-23 months include “and their associated factors”. Which MN? it is essential you specify what you are measuring is it Vitamin A supplementation or something else: MNPs, 24 dietary recall?

**Authors’ response:** Dear reviewer, thank you for your constructive comments. We have revised and clearly mentioned the recommended micronutrients which has been implemented in Ethiopia by the nutrition supplementation program in the introduction and method section besides the abstract section; kindly see in the abstract (page 2, lines 14-16), introduction (page 5, lines 64-68) and methods (page 9, lines 154-158) of the clean version of the revised manuscript.

L 25: this is too generic, need to specify which ‘recommended micronutrient supplements’. If more than just VAS, what else?

**Authors’ response:** Dear reviewer, thank you for this important feedback. We mentioned the contents of recommended micronutrient in the background section of the Abstract. To elaborate more, the recommend micronutrients which has been implemented to prevent the micronutrient deficiencies in Ethiopia includes foods rich in vitamin A, foods rich in iron, multiple micronutrient powder, iron and vitamin A supplements and or deworming medications (for those older than 24 months); please see the clean version of the revised manuscript on page 2, lines 14-16.

L 30: Again, still unclear whether you are describing VAS or MNPs or 24 hour dietary recall.

**Authors’ response:** Dear reviewer, thank you for your comments. These recommended micronutrients in the survey were assessed using the 24 hours dietary recall (for food rich in Vitamin A, iron), seven days recall (for the multiple micronutrient powder) and six months recall (for VAS and deworming) which is mentioned in the variable measurement section; kindly refer the clean version of the revised manuscript on pages 9-10, lines 154-184.

L31: i suggest "study population in this area", rather than "study area"

**Authors’ response:** Thank you for the suggestion and the issue is incorporated; kindly see on page 2, line 32.

L33: i suggest putting a full stop (.) after ‘community level’

**Authors’ response:** Thank you for the suggestion and the issue is addressed; kindly see on page 2, lines 33-36.

L35: This final sentence is a recommendation, please conclude your findings rather than just saying they were low in the previous sentence.

**Authors’ response:** Dear reviewer, thank you so much for your detail insights. You are right that the last sentence of the conclusion is recommendation which sated based on our findings. We perceived that putting a recommendation at the end of the conclusion after the finding gives sound for the readers and other policy makers that highlights the future importance of the study.

INTRODUCTION

L45: I suggest “micronutrients are”

**Authors’ response:** Thank you for the comment and the issue addressed; kindly see on page 4, line 45.

L50: I suggest “growth” instead of grow

**Authors’ response:** Thank you for the comment and the issue addressed; kindly see on page 4, line 50.

L55: please define Minimal Diversified Diets (MDD)

**Authors’ response:** Thank you for the comment and the issue addressed; kindly see on page 4, lines 55-60.

L66: You need to explain more about VAD, is the 14% corrected for inflammation? If so which methods of correction was used. 14% prevalence is considered ‘moderate VAD’

**Authors’ response:** Dear reviewer, thank you for your constructive comments. We have revised the national prevalence of vitamin A deficiency and the deficiency was subclinical. The previous reported prevalence of VAD is one of the regions and now we made necessary correction; kindly see the clean version of the revised manuscript on page 5, lines 73-76.

L74: What is the definition of “emerging regions”. where are they on a map? Please provide a map.

**Authors’ response:** Dear reviewer, thank you for your constructive comments and the issue is addressed; kindly see on page 6, lines 101-108 and figure 1.

L77: 'onset of' is superfluous

**Authors’ response:** Thank you for the comment and the issue is addressed.

L81: “therefore” is unnecessary

**Authors’ response:** Thank you for the suggestion and the word is removed.

L83: 'Moreover' is unnecessary

**Authors’ response:** Thank you for the comment and the issue is addressed.

L84: I suggest “problems”

**Authors’ response:** Thank you for the comment and the issue addressed; kindly see on page 6, line 95.

STUDY SETTINGS AND DATA SOURCES

L92: Why are they called 'emerging'. Please explain.

**Authors’ response:** Thank you for this important comment and the issue is addressed; kindly see on page 6, lines 101-108.

SAMPLING PROCEDURES

L108: I presume you mean 'one' segment rather than 'only a'.

**Authors’ response:** Thank you for pointing out it and the issue is addressed; kindly see on page 7, line 128.

L110: 'A fixed number of' is redundant. The sentence can start with 'twenty-eight'

**Authors’ response:** Thank you for the comment and the issue addressed; kindly see on page 8, line 131.

L112: What do the enumerators do if no-one was at home to be interviewed? or no children 6-23 within the household.

**Authors’ response:** Dear reviewer, thank you for this helpful feedback. The enumerators first labeled the households with children age 6-23 months collaborations with the health extension workers working in the community and women health development army leaders and then they conducted their interview after selecting the households systematically. If the no-one was at home, they revisit the household.

L117: Why female survey, were females the only targeted audience?

**Authors’ response:** Dear reviewer, thank you for your comments. Females are not the only target which was mentioned by mistake, now we made a correction; kindly see the clean version of the revised manuscript on page 8, lines 137-138.

L118: Please be consistent with the use of mothers/caregivers

**Authors’ response:** Thank you for the comment and we have revised the word thoroughly.

L119: The meaning of this sentence is unclear.

**Authors’ response:** Thank you for the comment and the issue is addressed; kindly see on page 8, lines 140-141.

L121: How was that one child of twins selected? By ballot?

**Authors’ response:** Dear reviewer, thank you for your constructive comment. The one child was selected by lottery.

L122: ‘extracted’ is an odd way of expressing this

Authors’ response: Thank you for the comment and the issue is addressed on line 143.

MEASUREMENTS OF VARIABLES

L130: Again, the reader can’t follow the augment clearly as you haven't defined what MNs and which preparations and recommended timeframes at the beginning

**Authors’ response:** Dear reviewer, thank you very much for this important comment. We describe the measurements of the recommended micronutrients; kindly see on pages 8-10, lines 150-184.

L131: add “/caregivers”

**Authors’ response:** Thank you for the suggestion and we have added it.

L137: Please clearly explain in background why deworming medication was included in this analysis MNs

**Authors’ response:** Dear reviewer, thank you for your constructive comments and which is really improve our manuscript. The Ethiopian nutrition supplementation program for children to prevent the deficiencies of micronutrients has been implemented in the form of foods and supplementation. This recommendation includes foods rich in vitamin A, foods rich in iron, multiple micronutrient powder, iron and vitamin A supplements and or deworming medications (deworming medication has been supplemented for those greater than 12 months of old children). Therefore, the deworming medication was included in the analysis because the national micronutrient supplementation has been including in its supplementation components, this is also included in the background and method section of our manuscript; please see the clean version of the revised manuscript.

L138: is this referring to any one of the recommended micronutrients, two or three?

**Authors’ response:** Dear reviewer, thank you for your insights. It referring about any one of the recommended micronutrients.

L159: Do the national programs start providing deworming medication at 6 months of age rather than more normally from 12 months of age. What was the deworming medication?

**Authors’ response:** Dear reviewer, thank you for this important feedback. The national program provides the deworming medication for those above 12 months children and also our study was measured the deworming medication for those above 12 months in which the number of children (n=547) for the assessment of deworming medication is different and mentioned in Table 4; kindly see Table 4.

L160: How reliable is the child health card as a source of information rather than the verbal history form the caregiver.

**Authors’ response:** Dear reviewer, thank you so much for this important comment. Using child health card as source of information might affects the reliable but it was used besides the mother’s verbal response. Moreover, we also mentioned as limitation about using the mothers verbal response particularly on recall bias; kindly see the on the methods (page 10, lines 183-184) and strength and limitation (page 27, lines 430-432) sections of the clean version of the revised manuscript.

L164: Do you mean obstetric or history and/or parity and/or ante-natal care?

**Authors’ response:** Dear reviewer, thank you for your comment. We tried to mention the obstetric characteristics of a woman like parity, number of living children and birth interval and the services taken during the antepartum (ANC), intrapartum (place of delivery) and postpartum (PNC). So, kindly see the clean version of the revised manuscript on page 10, lines 187-190.

L168: These indicators measured to reach your definition of wealth need to be described.

**Authors’ response:** Dear reviewer, thank you for the comment and we made revision accordingly; see on page 10, lines 196-197.

L169: Are these ranks comparable to international definitions or just in relation to the cohort under investigation. Are you really comparing very poor, poor, less poor?

**Authors’ response:** Dear reviewer, thank you for your constructive comments. The ranks are comparable to the international definitions. The cut points in the wealth index at which to form the quintiles are calculated by obtaining a weighted frequency distribution of households, the weight being the product of the number of de jure members of the household and the sampling weight of the household. Thus, the distribution represents the national household population, where each member is given the wealth index score of his or her household. The persons are then ordered by the score, and the distribution is divided at the points that form the five 20- percent sections. Then the household score is recoded into the quintile variable so that each member of a household also receives that household’s quintile category.

L170-1: This sentence is unclear.

**Authors’ response:** Dear reviewer thank you for the observation. We have revised the wealth quantile definition; please see the clean version of the revised manuscript on page 10, lines 195-198.

DATA PROCESSING AND STATISTICAL ANALYSIS

L185: Again, do you mean parity/access to ANC?

**Authors’ response:** Dear reviewer, thank you for the detail comments. Parity and access to ANC are two different variables and we tried to define them in the variables and measurements section; kindly see on page 10, lines 187-190.

RESULTS

L223: Again, were ALL the participants mothers of these children or were some caregivers?

**Authors’ response:** Dear reviewer, thank you very much for your detail observation. As per the EDHS data, we strongly believe that there might be few caregivers, which are not separately mentioned to quantify them. The data element mentioned as mothers/caregivers. So, kindly considered this during your review.

OBSTETRIC HISTORY OF PARTICIPANT

L236: please define the term “multipara”

**Authors’ response:** Dear reviewer, thank you. We mentioned the definition of the parity categories including multipara in the variables and measurements section of the methods. For more clarification, Party measures the number of births after 28 months of gestational weeks and it can be classified in to primipara (having only one birth), multipara (having 2-4 births) and grand multipara (having five and more births); kindly see on page 10, lines 187-190.

L237: acronym ANC not yet introduced and must be spelt in fill the first time it is used

**Authors’ response:** Dear reviewer, thank you for your suggestions. The full term of the acronym is first mentioned in the introduction section and the acronym used subsequently; kindly see clean version of the revised manuscript on page 5, line 80.

L239: acronym PNC not yet introduced and must be spelt in fill the first time it is used

**Authors’ response:** Dear reviewer, thank you for your suggestions. The full term of the acronym is first mentioned in the introduction section and the acronym used subsequently; kindly see clean version of the revised manuscript on page 5, line 81.

L241: Grand multipara must first be defined as mentioned above

**Authors’ response:** Dear reviewer, thank you for your comments. We have defined the grand multipara; please see the method section of the clean version of the revised manuscript on the page 10, lines 187-190.

CHILD CHARACTERISTICS AND COMMON CHILDHOOD ILLNESSES

L245: No definitions of average birth weight provided

**Authors’ response:** Dear reviewer, thank you for the comments and we mentioned the definition of it’s accepted; please see the clean version of the revised manuscript on page 10, lines 191-192.

L247: “were weighted” should be changed to 'were weighed'

**Authors’ response:** Thank you for the comments and it’s accepted; please see the clean version of the revised manuscript on page 16, line 274.

L247: please insert % after these figures

**Authors’ response:** Thank you for the comments and it’s accepted; please see the clean version of the revised manuscript on page 16, line 274.

L248: I presume you mean had 'either' rather than 'and'

**Authors’ response**: Thank you for the comments and it’s accepted; please see the clean version of the revised manuscript on page 16, line 275.

L251: I suggest adding “within last 2 weeks” for diarrhoea, cough and fever which is I believe the timeframe under consideration

**Authors’ response**: Thank you, this is hugely important and incorporated; kindly see Table 3 on page 16.

COMMUNITY LEVEL POVERTY, MEDIA EXPOSURE AND ACCESS TO HEALTH FACILITY

L254: ‘had had’ not ‘has’

**Authors’ response**: Dear reviewer, the comment is accepted and incorporated; see on line 280-1.

L256: do you mean ‘was’ or ‘was not’

**Authors’ response:** Dear reviewer, thank you. We mean that was not; please see the clean version of the revised manuscript on line 282.

MICRONUTRIENT INTAKE AMONG CHILDREN 6-59 MONTH

L258: I don't think you adequately described your definition of minimum recommended in background or methods.

**Authors’ response:** Dear reviewer, thank you very much for your constructive comments and observations. According to the Ethiopian nutrition supplementation program for children to prevent micronutrient deficiencies, we describe the minimum recommend micronutrients in the introduction (page 5, lines 64-68) and methods (page 9, lines 154-158) of the clean version of the revised manuscript.

DISCUSSION

I think this section need to be carefully rewritten to ensure it relates to the findings in a systematic manner.

**Authors’ response:** Dear reviewer, we are very thankful for your details comments and we have re-worked the discussion accordingly; kindly see the clean version of the revised manuscript on pages 24-26, lines 348-418.

L361: discuss the association to “care” in these regions. like active response feeding for this age group since they are young compare to the older age group, who might be considered “can eat for themselves” in a family meal!!

**Authors’ response:** Dear reviewer, thank you very much for your constructive comments and details insights. The issues is revied as per you comments; kindly see the clean version of the revised manuscript on page 25, lines 381-382.

CONCLUSIONS

L418-onwards are recommendations rather than conclusions based upon findings

**Authors’ response:** Dear reviewer, thank you for you comments. You are right, the last paragraph of the conclusion states the final recommendation of the study.

REFERENCES

Placed ensure you comply with instructions to authors.

**Authors’ response:** Thank you for this feedback we have taken a close look at the PLOS ONE Journal Editorial guidelines to ensure our article is in line with this.
