# Supplementary material for: Micronutrient intake status and associated factors among children aged 6–23 months in the emerging regions of Ethiopia: A multilevel analysis of the 2016 Ethiopia demographic and health survey
Source: PLoS One. 2021 Oct 22;16(10):e0258954. doi: 10.1371/journal.pone.0258954 (PMC8535338; doi:10.1371/journal.pone.0258954)
Supplement: Attachment — Submitted filename: PONE-D-20-13718 R3.docx [file pone.0258954.s005.docx]

**Micronutrient intake status and associated factors among children aged 6-23 month in the emerging regions of Ethiopia: a multilevel analysis of the 2016 Ethiopian demographic and health survey.**

Overall the article has improved but remains in need of focus in the significant findings and less insignificant details. Another thorough review by a native English speaker with a background in nutrition/public health would be beneficial. Being more concise should be prioritized as information of interest to readers is obscured by less important information.

Abstract

L 14: Are the national recommendations that a child should have all of these 6 options: a diet rich in VA and iron, plus NMP plus VAS and iron supplementation, plus deworming? If not please define the threshold recommendation (any one of the 6, 3 of the 6 or what?) before going further with the methods and results.

L 24: Please describe the 6 options considered for MN intake and the yes no response. Also you need to justify why deworming was included.

L 25: It would be more powerful to state the inverse of this: i.e. 37.3% (CI) had not received any to the recommenced MN sources/interventions

L 29: Residence in.

L 33: Although you have itemized the individual recommendations you have not clarified if there is a 'threshold' (national recommendations) to judge these findings against. This could be a better summary for the conclusion based upon the evidence you have generated. “After adjusting for individual and community level factors, women’s occupational status, child's age, antenatal visits for current pregnancy, residence and region were significantly associated with the micronutrient intake status among children aged 6 to 23 months”. The second sentence is not based upon the results presented and could be deleted, to be discussed in the main text as recommendations.

L 34: Unless more data is presented in the results, this is also not evident. Recommendations should be based on the evidence presented. What was the coverage for VAS, iron supplementation, diet rich in VA or diet rich in iron, deworming and ANC care in the study group. Only those that were especially low make this last sentence appropriate.

L 35: As a final sentence in conclusion again it doesn't make sense based upon the results presented: how do you conclude anything from attendance at ANC, working in the ag sector and children 13-23 months?

Introduction

L 38: Among children should come after deficiency.

L 39: I recommend you introduce the abbreviation (LMICs) and use it throughout the rest of the manuscript.

L 40: I am not sure the word commonest is the best word to use.

L 41: I think the rest of this sentence after vitamins in line 40 can be deleted and it is repetitious and doesn't add to the information.

L 42: Since you refer to MNP a lot I suggest you introduce the abreviation here.

L 44: Again, the word devastating could be better expressed by the word debilitating.

L 46: I suggest you convert this to the first 1000 days from conception to 2 years (hence ANC supplementation is so important)

L 48: Why use the word apparent?

L 49: Please replace the word most with actual percentages from a reference or delete this first sentence as the second sentence can 'stand-alone'.

L 53: This sentence is better without starting with ‘Nevertheless’

L 55: Reference, where does this date come from 29% of children in LMICs or Ethiopia or SSA or globally? please clarify

L 56: The middle of this sentence is unnecessary and unclear. You could delete after seven food groups' to 'namely.

L 69: It would be appropriate here to introduce the intervention taken to improve antenatal nutrition/supplementation of mothers.

L 71: MDD doesn’t read properly in this sentence as constructed. Please reconstruct.

L 72: Here you are describing a diversified diet. Is the reader to presume that is different from MDD?

L 76: Was this figure obtained by serological testing and corrected for inflammation and of so which correction method was used?

L 79: Place of residence

L 81: Visits is unnecessary if you use the term post-natal care.

L 89: No need to start the sentence with ‘Besides’

L 90: Rather than 'could worsen' I recommend ‘exacerbate’

L 91: This sentence needs 'associated with' MN intake

L 104: Categorized 'as'

L 106: No need to start the sentence with ‘Moreover’.

L 142: How was the selection of one twin only performed? By convenience?

L 151: Regarding onwards to the end of this sentence can be deleted

L 152: It wasn't 'measured' it was 'assessed'. I recommend deleting the rest of this sentence after 'based on' and going direct to the definitions in the next sentence starting at 'foods rich.

L 155: So there were 6 options: food rich in VA or iron in last 24 hours, MNP or iron supplement within 7 days, VAS or deworming within 6 months.

L 156: A shorter version of this definition of MN intake is what is missing in the Abstract. line 155-158.

L 158: If the respondent reported that the child had eaten' at least

L 165: If the respondent reported that the child had eaten' at least

L 169: If the respondent reported that the child had eaten' at least

L 171: I think you can change all these to 'respondent'.

L 172: This sentence is more suitable for introduction not methods and you cannot say 'nearly always in a scientific manuscript: check what is used in the emerging region: MNPs were developed to address iron deficiency

L 174: No need to use the brand name.

L 177: How will a respondent know if it was with iron?

L 179: This first sentence should be merged with introduction not methods

L183: This is unclear, was a verbal response accepted even in the absence of card confirmation?

L 186: These first 2 sentences,185-187 should be merged with introduction not methods

L 188: A single birth

L190: Unclear, please explain your definition of question asked and options for respondents.

L 191: Were weights taken at the time of the EDHS 2016?

L 192: In an emerging region I would not expect many children had been weighed at birth. So how was this information obtained? from a child health card?

L 194: How was poverty classified? If you mean wealth quintile then say so.

L 198: Are these comparable to the World Bank definitions?

L 202: Please describe the community-level factors in the same order as cited in Line 194.

Result

L 248: Please present your findings more concisely. If the basic data can be found in the table and there is nothing of note to bring to the reader’s attention then just to refer to Table 1

L 256: You have included some of the community level indicators but not all: media exposure, distance to health facility and have another section later on community level indicators

L 262: Gave birth to their first child

L 263: Overall 45.2% 2-5 living children is different to the definitions given in methods of multip/grand multipara.

L 264: This sentence is poorly written and needs to be revised by the authors.

L 267: For ANC to you mean never had any ANC visits vs any number of ANC visits?

L 270: Be more concise, no need to recite everything that can be found in Table 2. Draw the readers attention to the important points only.

L 277 Table 3: This row can be deleted

L 280: Be consistent, sometimes you use % and sometime percent.

L 283: Be concise and draw attention to points of interest rather than stating everything that is in Table 4.

L 284: As stated in the Abstract it makes more impact to state the inverse, those that had none any of the 6 options.

L 287: Add these CIs to Table 4

L 279: Table 4: ‘Not receive’ column is unnecessary. What is your definition of the minimum?

L 323: This statement would be better in the Abstracts conclusion

L 330: Did this take distance to health facility into consideration?

L 334: Do you mean any MN? If not please explain

L 337: Again, what do you mean by ‘recommended’: any one of the 6 options or at least 3 of the 6 options? or what?

Discussion

L 348: Revise this first sentence to show the inverse finding 37.3% had none of the MNs recommended.

L 350: You are repeating the results, that is ok provided you only repeat the important findings not just dimple percentages. After adjusting for individual and community level factors, women's occupational status, the child's age, antenatal visits for current pregnancy, residence and region were significantly associated with the MN intake status among children aged 6 to 23 months.

L 356: This sentence by itself does not make sense to the global reader unfamiliar with these phases.

L 362: I don't think comparing Ethiopia with India makes sense, concentrate on data from SSA. Also focus on the results after adjusting for individual and community level factors, you are losing the important findings in amongst less important findings.

L 363: Much lower for what?

L 365: For what?

L 367: Of what? I think these statements line 357-370 were already made in the introduction

L 377: Where?

L 380: Better described as poor complementary feeding practices that should be introduced at 6 months of age.

Conclusion

L 440: This last statement excluded any reference to ANC, promoting affordable, available MN-rich foods through improved/adaptive agricultural practices, deworming and MNPs (the other options).

References

I believe 63 references is excessive for a manuscript of this nature. Please be more selective. I suggest 40 well-chosen references would be sufficient.
