# Supplementary material for: Micronutrient intake status and associated factors among children aged 6–23 months in the emerging regions of Ethiopia: A multilevel analysis of the 2016 Ethiopia demographic and health survey
Source: PLoS One. 2021 Oct 22;16(10):e0258954. doi: 10.1371/journal.pone.0258954 (PMC8535338; doi:10.1371/journal.pone.0258954)
Supplement: Attachment — Submitted filename: Response to reviewers.doc [file pone.0258954.s006.doc]

**Response to editor’s and reviewers’ comments**

I) **Response to academic editor’s comments**

Overall, the article has improved but remains in need of focus in the significant findings and less insignificant details. Another thorough review by a native English speaker with a background in nutrition/public health would be beneficial. Being more concise should be prioritized as information of interest to readers is obscured by less important information.

**Authors’ response:** Dear editor, thank you for your time to go thoroughly to comment our manuscript for improvement’ We have learnt a lot of things form each subsequent comments and suggestion provided before. The manuscript is reviewed and edited by fluent English speaker and at this stage, we believe that we incorporate all the concerns raised.

Abstract

L 14: Are the national recommendations that a child should have all of these 6 options: a diet rich in VA and iron, plus NMP plus VAS and iron supplementation, plus deworming? If not please define the threshold recommendation (any one of the 6, 3 of the 6 or what?) before going further with the methods and results.

**Response:** Dear editor, we are very much thankful for your comments. The national recommendation of micronutrients is that a child should have 6 options: a diet rich in vitamin A and iron, plus multiple micronutrient powder plus vitamin A and iron supplementation, plus deworming.

L 24: Please describe the 6 options considered for MN intake and the yes no response. Also you need to justify why deworming was included.

**Response:** Dear editor, thank you for the comments. We describe the 6 options on page 2, line 14-16 and 21-22. As per our description in the background section and method, the recommended micronutrient in the national nutrition program includes a diet rich in vitamin A and iron, plus multiple micronutrient powder plus Vitamin A and iron supplementation, plus deworming (for children older than 12 months). Therefore, we included the deworming for those children aged greater than 12 months only which is really did no have any significant effect on the outcome measurement or estimation as per the figure but we included it to show the national micronutrient recommendation figure which will be helpful for decision making among program implementors and also for the services improvement purpose. We believe that it might not considered as a micronutrient component but we mention it as a limitation; kindly consider in such a way.

L 25: It would be more powerful to state the inverse of this: i.e. 37.3% (CI) had not received any to the recommenced MN sources/interventions

**Response**: The comment is addressed; kindly see line 26.

L 29: Residence in.

**Response:** The comment is addressed; please see line 31.

L 33: Although you have itemized the individual recommendations you have not clarified if there is a 'threshold' (national recommendations) to judge these findings against. This could be a better summary for the conclusion based upon the evidence you have generated. “After adjusting for individual and community level factors, women’s occupational status, child's age, antenatal visits for current pregnancy, residence and region were significantly associated with the micronutrient intake status among children aged 6 to 23 months”. The second sentence is not based upon the results presented and could be deleted, to be discussed in the main text as recommendations.

**Response:** The comments are addressed; kindly see the conclusion section in the Abstract on line 34-38.

L 34: Unless more data is presented in the results, this is also not evident. Recommendations should be based on the evidence presented. What was the coverage for VAS, iron supplementation, diet rich in VA or diet rich in iron, deworming and ANC care in the study group. Only those that were especially low make this last sentence appropriate.

**Response:** Dear editor, thank you for your detail observations and we have revised it accordingly; kindly see the conclusion section in the Abstract from line 34-38.

L 35: As a final sentence in conclusion again it doesn't make sense based upon the results presented: how do you conclude anything from attendance at ANC, working in the ag sector and children 13-23 months?

**Response**: Dear editor, the conclusion is revised as per the comments.

Introduction

L 38: Among children should come after deficiency.

**Response:** The comment is addressed; kindly see line 40.

L 39: I recommend you introduce the abbreviation (LMICs) and use it throughout the rest of the manuscript.

**Response**: The comment is incorporated; see on line 41.

L 40: I am not sure the word commonest is the best word to use.

**Response:** Dear editor, we revised the word and changed by essential; kindly see on line 42.

L 41: I think the rest of this sentence after vitamins in line 40 can be deleted and it is repetitious and doesn't add to the information.

**Response:** Dear editor, thank you for your observation and the comments are addressed.

L 42: Since you refer to MNP a lot I suggest you introduce the abbreviation here.

**Response:** Dear editor, thank you for your comments. We revised all the words with their abbreviations and used throughout the manuscript as needed.

L 44: Again, the word devastating could be better expressed by the word debilitating.

**Response:** The comment is incorporated; see line 45.

L 46: I suggest you convert this to the first 1000 days from conception to 2 years (hence ANC supplementation is so important)

**Response:** Dear editor, thank you for your insights. We revised it accordingly; please see line 46.

L 48: Why use the word apparent?

**Response**: Dear editor, we revised it; kindly see line 46-48.

L 49: Please replace the word most with actual percentages from a reference or delete this first sentence as the second sentence can 'stand-alone'.

Response: Dear editor, the first sentence is deleted and started by the second on line 49.

L 53: This sentence is better without starting with ‘Nevertheless’

**Response:** The comment is addressed, line 51-52.

L 55: Reference, where does this date come from 29% of children in LMICs or Ethiopia or SSA or globally? please clarify

**Response:** Dear editor, thank you for your constructive comment. We have revised and addressed the issue; kindly see line 52.

L 56: The middle of this sentence is unnecessary and unclear. You could delete after seven food groups' to 'namely.

**Response:** Dear editor, thank you for your constructive comments and we addressed accordingly, see line 52-55.

L 69: It would be appropriate here to introduce the intervention taken to improve antenatal nutrition/supplementation of mothers.

**Response:** Dear editor, thank you for your constructive comment. We have introduced the interventions which have been implemented in Ethiopia to improve antenatal nutrition/supplementation of mothers; kindly see line 65-67.

L 71: MDD doesn’t read properly in this sentence as constructed. Please reconstruct.

**Response**: We reconstructed it; kindly see on line 68-69.

L 72: Here you are describing a diversified diet. Is the reader to presume that is different from MDD?

**Response:** Dear editor, thank you for the comment. We believe that the previous sentence (Line 68-69) can describe the magnitude of MDD and we removed the second sentence.

L 76: Was this figure obtained by serological testing and corrected for inflammation and of so which correction method was used?

**Response:** Dear editor, thank you for this constructive comment. The finding was obtained by the retinol analysis and those who had serum retinol of less than 0.7 μmol/L were diagnosed with subclinical Vitamin A deficiency. But the result was not corrected for inflammation.

L 79: Place of residence

**Response:** The comment is addressed on line 75.

L 81: Visits is unnecessary if you use the term post-natal care.

**Response:** The comment is addressed on line 77.

L 89: No need to start the sentence with ‘Besides’

**Response:** The comment is addressed; see line 84.

L 90: Rather than 'could worsen' I recommend ‘exacerbate’

**Response:** Thank you for the recommendation and the comment is addressed on line 85.

L 91: This sentence needs 'associated with' MN intake

**Response:** Thank you and the comment is addressed on line 86.

L 104: Categorized 'as'

**Response:** Thank you, the comment is addressed on line 98.

L 106: No need to start the sentence with ‘Moreover’.

**Response:** We removed the word ‘Moreover’ from line ----

L 142: How was the selection of one twin only performed? By convenience?

**Response:** Dear editor, thank you for this constructive comment. Indeed, the selection of the twin was convenience and we mentioned it on line 133.

L 151: Regarding onwards to the end of this sentence can be deleted

**Response:** Then you for the comment, we removed it as per the comments; kindle see on line 141-142.

L 152: It wasn't 'measured' it was 'assessed'. I recommend deleting the rest of this sentence after 'based on' and going direct to the definitions in the next sentence starting at 'foods rich.

**Response:** Dear editor, thank you for your detail comments. We revised it accordingly; please see line 142.

L 155: So, there were 6 options: food rich in VA or iron in last 24 hours, MNP or iron supplement within 7 days, VAS or deworming within 6 months.

**Response**: We incorporate the comment on line 143.

L 156: A shorter version of this definition of MN intake is what is missing in the Abstract. line 155-158.

**Response:** We apricate the comment and the short version is included in the Abstract on line 14-16.

L 158: If the respondent reported that the child had eaten' at least

**Response:** The comment is addressed on line 145.

L 165: If the respondent reported that the child had eaten at least

**Response:** The comment is addressed on line 151.

L 169: If the respondent reported that the child had eaten' at least

**Response:** The comment is addressed on line 156.

L 171: I think you can change all these to 'respondent'.

**Response:** Dear editor, thank you for your comment. We revised accordingly.

L 172: This sentence is more suitable for introduction not methods and you cannot say 'nearly always in a scientific manuscript: check what is used in the emerging region: MNPs were developed to address iron deficiency

**Response**: Dear editor, thank you for the comment and we revised it and taken to the introduction section.

L 174: No need to use the brand name.

**Response**: The comment is addressed.

L 177: How will a respondent know if it was with iron?

**Response:** Dear editor, thank you for this comment. In the case of iron and vitamin supplementation, the mothers are mostly informed before the administration, so, we believe the women are able to know this especially the Vitamin A and Iron is easy to know and recall.

L 179: This first sentence should be merged with introduction not methods

**Response**: The comment is addressed and taken to the Introduction section on line 63-65.

L183: This is unclear, was a verbal response accepted even in the absence of card confirmation?

**Response:** Dear editor, thank you for this insight. The vitamin A supplementation status of the child was mainly collected from the child card, but sometimes the card may not available and mothers can recall on whether her child taken or not. In fact, there might be a recall bias, which is mentioned in the limitation section.

L 186: These first 2 sentences,185-187 should be merged with introduction not methods

**Response**: Dear editor, we are very much thankful for your comments and we revised it accordingly, see line 73-77.

L 188: A single birth

**Response**: The comment is addressed, see line 166.

L190: Unclear, please explain your definition of question asked and options for respondents.

**Response:** Dear editor, thank you. In our analysis, obstetrics services meaning the use of maternal services like ANC, institutional delivery and postnatal care and the response was; ‘yes’ (if they received the services) or ‘No’ (if not received the services).

L 191: Were weights taken at the time of the EDHS 2016?

**Response:** Dear editor, thank you for this comments and weights were taken during the survey.

L 192: In an emerging region I would not expect many children had been weighed at birth. So how was this information obtained? from a child health card?

**Response:** Dear reviewer, thank you for your detail observation. You are right, they might not weighed, but since this is a secondary data analysis we do not do anything to cross check at this stage. But in Ethiopian, if a mother did not deliver her baby at health facility, the health extension workers in the community have been visited at home within a week since they have a pregnant mothers list in her catchment areas and she follows frequently those pregnant women. The data on birth weight were collected based on a child card (if the card exists) or from mother’s verbal response if she recalls exactly, otherwise mostly the health care providers told to the mother in such a way; your baby birth weight is small or normal or big.

L 194: How was poverty classified? If you mean wealth quintile then say so.

**Response**: Dear editor, thank you for this comment and we mean wealth quintile, which is addressed on line 178.

L 198: Are these comparable to the World Bank definitions?

**Response:** Dear editor, thank you for your comment. The wealth quantiles measurements are context dependents; it might not be comparable with the world bank definition.

L 202: Please describe the community-level factors in the same order as cited in Line 194.

**Response:** The comments are addressed, see line 178-187.

Result

L 248: Please present your findings more concisely. If the basic data can be found in the table and there is nothing of note to bring to the reader’s attention then just to refer to Table 1

**Response:** Dear editor, thank you for your constructive comments and we revised accordingly, please see the results section on line 228-232 and Table 1.

L 256: You have included some of the community level indicators but not all: media exposure, distance to health facility and have another section later on community level indicators

**Response:** Dear editor, thank you for the comments. We revised and presented all the community level factors in Table 4.

L 262: Gave birth to their first child

**Response:** The comment is addressed; kindle see on line 239.

L 263: Overall 45.2% 2-5 living children is different to the definitions given in methods of multip/grand multipara.

**Response:** Dear editor, thank you for your comments. The multiparty and grand parity in our method was defined based on the number of deliveries after 28 weeks of gestation. Accordingly, women who have 2 to 4 births after 28 weeks of gestion we classified as multiparity and more than 4 grand parity. But the living children was measured the number of alive children currently. So, those two variables are different; kindly referee our result based on this definition.

L 264: This sentence is poorly written and needs to be revised by the authors.

**Response:** The sentence is rewritten; please see on line 241.

L 267: For ANC to you mean never had any ANC visits vs any number of ANC visits?

**Response:** Dear Editor, thank you for this comment. ANC visit was asked for their recent birth and labeled as Yes or no; no mean never had any ANC visit.

L 270: Be more concise, no need to recite everything that can be found in Table 2. Draw the readers attention to the important points only.

**Response**: Dear editor, we revised it and all the narration before the Tables; kindly see the results sections.

L 277 Table 3: This row can be deleted

**Response:** Dear editor, thank you for the comment and we revised the Table accordingly, see Table 3.

L 280: Be consistent, sometimes you use % and sometime percent.

**Response:** Dear editor, thank you for the detail observation. We revised it, but in few cases we used the percent instead of % at the beginning of a new sentence and or paragraph, otherwise we used % consistently.

L 283: Be concise and draw attention to points of interest rather than stating everything that is in Table 4.

**Response**: Dear editor, we revised it accordingly; kindly see Table 5 and line 257-266.

L 284: As stated in the Abstract it makes more impact to state the inverse, those that had none any of the 6 options.

**Response:** The comment is addressed on line 258.

L 287: Add these CIs to Table 4

**Response:** The comments are addressed; see Table 5 of the revised version on page 16

L 279: Table 4: ‘Not receive’ column is unnecessary. What is your definition of the minimum?

**Response:** Dear editor, thank you for the comments. We removed the column; kindly see Table 5 on page 16 and we revised the minimum by at least one of the recommended micronutrients.

L 323: This statement would be better in the Abstracts conclusion

**Response:** The comment is addressed.

L 330: Did this take distance to health facility into consideration?

**Response:** Dear editor, thank you for this comment. Distance to health facility did not take into consideration.

L 334: Do you mean any MN? If not please explain

**Response:** The comment is addressed on line 296.

L 337: Again, what do you mean by ‘recommended’: any one of the 6 options or at least 3 of the 6 options? or what?

**Response:** The recommended mean any one of the 6 options and the comment is addressed on line 299.

Discussion

L 348: Revise this first sentence to show the inverse finding 37.3% had none of the MNs recommended.

**Response:** Dear editor, thank you for your constructive comment and we have addressed it on line 308.

L 350: You are repeating the results, that is ok provided you only repeat the important findings not just dimple percentages. After adjusting for individual and community level factors, women's occupational status, the child's age, antenatal visits for current pregnancy, residence and region were significantly associated with the MN intake status among children aged 6 to 23 months.

**Response:** The suggestion is incorporated on line 309-312.

L 356: This sentence by itself does not make sense to the global reader unfamiliar with these phases.

**Response:** Dear editor, thank you for the comment and we have revised it.

L 362: I don't think comparing Ethiopia with India makes sense, concentrate on data from SSA. Also focus on the results after adjusting for individual and community level factors, you are losing the important findings in amongst less important findings.

**Response:** The comment is addressed and we compared the findings from Nigeria’s on line 339.

L 363: Much lower for what?

**Response:** Dear editor, we revised and edited the discussion section.

L 365: For what?

**Response:** Dear editor, we revised and edited the discussion section, so there are changes in paragraphs and sentences.

L 367: Of what? I think these statements line 357-370 were already made in the introduction

**Response:** Dear editor, thank you for the comments. We revised and edited the discussion section, so there are changes in paragraphs and sentences.

L 377: Where?

**Response:** The comment is addressed on line 325-326.

L 380: Better described as poor complementary feeding practices that should be introduced at 6 months of age.

**Response:** The comment is addressed on line 328.

Conclusion

L 440: This last statement excluded any reference to ANC, promoting affordable, available MN-rich foods through improved/adaptive agricultural practices, deworming and MNPs (the other options).

**Response:** The comment is addressed; kindly see line 387-393.

References

I believe 63 references is excessive for a manuscript of this nature. Please be more selective. I suggest 40 well-chosen references would be sufficient.

**Response:** Dear editor, thank you for your detail and constructive comments. We tried to choose the pertinent references.

II) **Responses to reviewers’ comments**

**Reviewer #2 comments**

Abstract- The author has not been too clear with his conclusion. He says ‘The micronutrient intake status in the study population in this area was low compared to the national recommendation. Promoting vitamin, A and iron-rich foods and micronutrient powders are better for micronutrient enrichments. Strengthen supplementations and deworming alongside the community-based maternal and child health services would improve micronutrient intake among children. There are a couple of typos here and grammatical errors that need to be addressed to make the conclusion concise. The author could try and rephrase the conclusion to read: The status of micronutrient intake among the study population was low when compared to the national recommended threshold. Strengthening supplementation and deworming in addition to maternal and child health services would improve micronutrient intake among children

**Response:** Dear reviewer, thank you for your comments and we addressed it; kindly see the Abstract on line 11-38.

Line 141-142: In the sampling procedure, the author has not explained why mothers with twins were only interviewed for one child. The author could consider explaining this sampling method for easy comprehension of sampling at the household for readers.

**Response:** Dear reviewer, thank you for your detail and constructive comments. In our analysis, mothers with twins were only interviewed for one child, because we believe that those twins might have the same micronutrient intake status.

Line 285-286: This sentence does not read well. The author could rephrase the sentence to read- Of those who took the recommended micronutrient, only 12.9% had received three or more types of micronutrients

**Response**: Dear reviewer, thank you for your comments, we have rewritten on those sentences and there are some sentence changes as per the Editor’s comments.

Line 293-294: There are a few typos in this sentence: “Around 47% (95% CI: 44.1-50.3) of the children 293 received vitamin A supplements in the six months preceding the interview. Besides, 8.4% (95% 294 CI: 5.5-8.7) of the children aged 12 to 23 months of were received deworming medication in the six months preceding the interview (Table 4).” The author could rewrite to make it easier for readers to understand what he is trying to say.

**Response:** Dear reviewer, thank you for your comment. We revised and rewritten the sentence; kindly see on line 258-264.

Line 361-362: Vitamin A supplement in Nigeria was 45% which is comparable with the current finding [53]. However, our study differs from the finding in India (30.4%) [54]. I suggest the author sticks with studies whose results are comparable to this study. Only compare finding in Nigeria to this and take out the study in India.

**Response:** The comment is addressed and we changed some of the sentence and the comparison was done form Nigeria.

Line 363: This study's result is much lower than that of the national target of over 90% [55]. The way this sentence is written is not grammatically sound. The author could consider re-writing to read: “Results from this study shows much lower micro nutrients uptake compared to the national target of over 90%

**Response:** Dear reviewer, thank you for your comments. We made changes on this sentence.

Line 372, 388-391, 398-399 either are not writing in proper English or are not clear. The author need to rewrite again to make them readable and clear to the reader. I have made some suggestions here. Instead of saying micronutrient intake among children whose mothers worked in agriculture was higher than children whose mothers did not have work; Rephrase sentence to read – Mothers who were housewives or who had no formal/paid jobs
Replace Also, even if vitamin A supplement is effective for 6-11 months, especially when used with the vaccine against measles…. With when administered with measles vaccines
Instead of saying Furthermore, they might learn about the value of iron intake that supplements, the author could re-write sentence to read: caregivers may have learned or acquired knowledge of iron supplements during their ANC follow-up.

**Response**: Dear reviewer, thank you for your comments. The comments are addressed accordingly; see line 319, 336-339, 345-346.

Line 412-413: Again, instead of saying” Besides, since the latter two regions have dense forests and water reservoirs, they could get wild fruit and fish, which are good micronutrients” the author should rephrase as “Besides, since the latter two regions have dense forests and water reservoirs, caregivers could get wild fruit and fish, which are good sources of micronutrients”
I recommend the author to read the whole manuscript again and ensure consistency in presenting results. Adjust all percentages to 1 decimal point.
**Response:** Dear reviewer, thank you for your comments and recommendation. We revised the results and all the decimals points also adjusted to 1.

Once more I suggest the author gets a native English speaker, to proof read the manuscript and ensure it is written in clear, simple English language.

**Reviewer #3 comments**

Abstract
L14: I suggest you add "include" before the word food
Response: Dear reviewer, thank you for your comments and the sentence is rewritten; kindly see line 14-16.

Conclusion
L 33: What is the national recommended threshold?

**Response:** Dear reviewer, thank you for your comment. The national recommended is a child should receive foods rich in vitamin A (VA) and iron, multiple micronutrient powder (MNP), iron and vitamin A supplements (VAS) and or deworming have been implemented in the form of food and supplementation. Their timing is also food rich in VA or iron in last 24 hours, MNP or iron supplement within 7 days, VAS or deworming within 6 months.

L 34: Supplementation of what?
**Response**: The sentences in the conclusion section of the Abstract are changed; kindly see line 34-38.

Introduction
L 75: This target should be sighted in the abstract above, L33, of my previous comment
**Response:** Dear reviewer, thank you for your comment and suggestions.

Measurement of Variables
L 174: Were the mothers shown a sample of the sachet during the interview?

**Response:** Dear reviewer, thank you for this comment. The mothers were expected to show the sachet during the interview for verification and if incase they discard or don’t have at that time, they were asked to described about it.

L 182: I suggest including the word "health" before the word "card"
**Response:** The comment is addressed on line 163.

Micronutrient intake status among children aged 6-23 months
L 294: I suggest deleting "of were"

**Response:** Dear reviewer, thank you for your suggestions. We made a revision on the result to make it clearer and simpler for the readers.
