# Supplementary material for: Micronutrient intake status and associated factors among children aged 6–23 months in the emerging regions of Ethiopia: A multilevel analysis of the 2016 Ethiopia demographic and health survey
Source: PLoS One. 2021 Oct 22;16(10):e0258954. doi: 10.1371/journal.pone.0258954 (PMC8535338; doi:10.1371/journal.pone.0258954)
Supplement: Attachment — Submitted filename: Response to reviewers comments.doc [file pone.0258954.s007.doc]

**Point by point response for reviewer’s comments**

|  | Reviewer’s comments | Authors’ responses |
| --- | --- | --- |
|  | Reviewer #2: Abstract, conclusion |  |
| 1. | L35: Although you have now clearly defined the sources of MN you have not defined the national recommendations. is it that all individuals should have all of these interventions? | Dear reviewer, thank you for your comments. As we defined in the introduction section (lines 59-63), these should be given for children older than six months and specifically for the deworming for those older than 12 months of age as per the national guideline. |
|  | Introduction |  |
| 2. | L42: Include (there are others) | Thank you for the comments and addressed on L42-43. |
| 3. | L48: The last part of this sentence needs amending | We appreciate the comment and incorporated on L48 |
| 4. | L50: Instead of saying vitamins and minerals why not stay with MN? | Thanks, we accept the comment and addressed on L50 |
| 5. | L51: At the beginning of this sentence, it would be good to state which year(s) you are referring to. | Thank you for the comments and addressed on L51 |
| 6. | L72: Please state whether this was corrected for inflammation and if so by which method. | Dear reviewer, we really appreciate the comment. The study measured the subclinical vitamin A deficiency using a cutoff point of serum retinol < 0.7 μmol/L as recommended by WHO, but other correction for inflammation was not done. Please see on line 72. |
| 7. | L75: Rather than 'on top of' I suggest 'in addition to' | Thanks, and addressed on L75 |
| 8. | L89: Problem (no s) | Incorporated accordingly on L89 |
| 9. | L131: 'Those second' do you mean 'those years old and older'? | Dear reviewer, thank you for your concern and we want to mentioned that the second and third child which means for those more than one child less than 24months for a mother, the first included but the second and third children were excluded. Kindly see on line 130-132. |
| 10. | L133: For mother /caregivers with twins only one was selected by convenience | Thank you and we amended it accordingly on L133 |
| 11. | L183: You mean 9 instead of an television? | Dear reviewer, thank you for your comments. We mean that the community media exposure was assessed;  “yes” if they have access to all three media (newsletter, radio, and television) at least once a week, otherwise “no” if they did not have any media exposure.  So, the three media includes newsletter, radio, and television. Without crusty, we did not mean 9. Kindly see it again on L183. |
| 12. | L240: Having defined grand multi para please use it here, unless there was a discrepancy between giving birth and living children. | Dear reviewer, thank you for your comments and we totally agree with that giving birth and living children are not the same and we corrected as per your comments; kindly see on L241. |
| 13. | L247: I recommend reducing this text further by referring the reader to the table. Only points of interest in the final analysis need a mention which does not include average birth weight and diarrhoea. However, the final analysis does include mothers occupation etc. | Dear reviewer, thanks for the comments. We addressed the comments; kindly see on L249-250 |
| 14. | L251: In the text you are using the word weight and here you are referring to size. Please clarify again as earlier you actually specified weight in kg whereas previously, I thought this was a subjective assessment by the mother/caregiver. | Dear reviewer, thank you for your comments. We are referring weight. Mother who gave birth at health facility remembered their child’s birth weight, but for those who gave birth at home measurement was done at home by the health extension workers within the first week which is one of the packages of community-based newborn care program in this case, the weight assessment might be a subjective assessment by the mothers to recall exactly, but they were informed whether their newborn was small, normal or over wight which makes subjective. So, we address the comments accordingly; kindly see table 3, on page 14, L253. |
| 15. | L259: Rather than 'Moreover' use 'Only' | Thank you. Addressed on L262 |
| 16. | L261: Better to continue the sentence rather than break it with 'additionally' | Thank you for the comments and incorporated on L264 |
| 17. | L286: Rather than antenatal visit stay with ANC | Thank you. Addressed on L291 |
| 18. | L300: I believe the manuscript would make a bigger impact if you limit this table to only those findings of statistical significance. The text could then simply stat that the other characteristics were not significant. | Dear reviewer, thank you for suggestions and we revised the table accordingly; kindly see on L306 |
|  | Discussion |  |
| 19. | L312: I see you have dropped the child’s weight now but there is still confusion over whether you were recording weight or size. Add had consumed | Dear reviewer, thank you for your comments. The child’s weight is not statistically significant in our analysis and as we defined in the variable measurement subheading of Methods, the child’s weight at the time of survey was measured. So, we can say that we mentioned the child’s weight at the result section but not in the discussion. |
| 20. | L313: Do you mean or iron? No need for the word 'reports' | Dear reviewer, thank you for your comments. We mean that in our finding, 28% of children had consumed foods rich in Vitamin A and 15.6% had consumed foods rich in iron. We also removed the word ‘reports’; kindly see on L317-318. |
| 21. | L315: No need to start this sentence with 'besides' | Thanks, addressed on L319 |
| 22. | L316: Got, not get. | Thank you. Addressed on L320 |
| 23. | L318: Current result? are you comparing EDHS 2011 with 2016 or with this analysis? | Dear reviewer, thank you for your comments. We compared the EDHS 2011 findings with our analysis/findings; kindly see on L321 |
| 24. | L322: Use MN | Thank you for the comment and addressed on 326 |
| 25. | L326: Rather than 'the current finding' I suggest 'this study' | Thank you for your suggestion and we amended it accordingly on L330 |
| 26. | L329: No need for 'in Ethiopia' | Thank you, addressed on L333 |
| 27. | L331: The sentence starting on L331-332 seems redundant although the citations might be useful | Dear Reviewer, thank you for your observation and we revised it accordingly; kindly see on L335 |
| 28. | L332: Again, starting the sentence with 'on the other way' is redundant. | Thank you for the comments and we addressed it on L336 |
| 29. | L334: What do you mean by low ability? | Dear reviewer, thank you for your insights. We mean that some of the mothers perceived that those children less than 23 months might be unable to take theses diversified food, because of those they reduced the varieties of food for children which can really affects the diversity of the food intake. We made a revision on it; kindly see on L337-338 |
| 30. | L338: The sentence starting on L336-339 does not make sense. Why would children not be eligible for VAS | Dear reviewer, thank you for your comments. We revised it and the sentence does not have any contribution at this place and we removed it. |
| 31. | L344: Another explanation might be that mothers with follow-up live nearer to health facilities of have more time/money available to attend ANC. | Thank you for your insights. We incorporated accordingly; see on L345-347 |
| 32. | L347: Same comment as above | Thank you for the comments and revised. |
| 33. | L351: A few | Thank you. Addressed on L352 |
|  | Strength and limitations of the study |  |
| 34. | L368: No need to expand this sentence. You can stop after factors. | Thank you and addressed on L369 |
| 35. | L369: No need to start the sentence with 'besides' | Addressed on L369 |
| 36. | L372: The last sentence in this para can be simplified to 'the DHS methodology allows for comparison with other settings'. | Thank you for your suggestions and incorporated on L371 |
| 37. | L373: This sentence is redundant as it is neither a strength not a weakness. | Dear Reviewer, thank you for your comments and we revised it and deleted. |
| 38. | L375: This second sentence can be removed as you are not assessing adequacy only the MN intake of MN recommendations. | Thank you for your comments. Removed accordingly. |
| 39. | L376: No need to start with 'furthermore' | Addressed on L372 |
| 40. | L377: Here you are using VAS but not in previous sections. Once an abbreviation has been introduced please ALWAYS use it. | Thank you for your observation and we revised all the abbreviations throughout the document and we made it consistent. |
| 41. | L378: I don't feel the last sentence is required. | Dear reviewer, thank you for your insights and we removed it accordingly. |
| 42. | Conclusion L381: Conclusion should be only 2-3 sentences just starting the essential facts not going over the details again. Some of what you say here could be in the discussion (but not all) | Dear reviewer, thank you very much for your comments. We made a revision for the conclusion; kindly see it once again on L375-381 |
| 43. | References L4023: Please review these citations and ensure only those of importance are used and that none are used twice. | Thank you for your comments and we checked the references and made the necessary corrections. |
