# Supplementary material for: Micronutrient intake status and associated factors among children aged 6–23 months in the emerging regions of Ethiopia: A multilevel analysis of the 2016 Ethiopia demographic and health survey
Source: PLoS One. 2021 Oct 22;16(10):e0258954. doi: 10.1371/journal.pone.0258954 (PMC8535338; doi:10.1371/journal.pone.0258954)
Supplement: Attachment — Submitted filename: Responses to Reviewers letter.docx [file pone.0258954.s008.docx]

**Responses to Reviewers letter /Point by point responses to editor’s comments**

**#1.** You have made some improvements but there is still too much information being presented in Tables 2 and 3 that have no relevance to your final analysis.

**Response:** Dear editor, we are very much thankful for your comments and suggestions that you provided for the improvements of our manuscript. Indeed, we agree with your suggestion and we reduced the non-significant variables both in the bivariable and multivariable analysis. Accordingly, the data presented in Table 2 and 3 are those significant in the bivariable analysis and we believe that those variables can have a better information for further researchers and policy makers. So, we keep in table 2 and 3 those variables with a p-value of less than 0.2 in the bivariable analysis and few of them are statistically significant at p-vale of less than 0.05 in the multivariable analysis.

**#2.** Confusion over how you have defined wealth status/index. quintile/quantile and birth weight/size remain.

**Response:** Dear editor, thank you for your comments. We addressed and made a clear and consistent definition of the two terms; household wealth index, community level wealth quantile. Kindly see the clean version of the manuscript on pages 9-10, lines 180-192.

**#3.** If data in tables 2 and 3 have been analyzed and found 'Not Significant' I suggest to reduce the amount of data being presented to the reader so that attention can be drawn to important findings: mothers’ occupation, ANC visits, child's age, residence and region.

**Response:** Thank you for the suggestion. We reduced those non-significant variables at the bivariable analysis; kindly see Table 2 on page 13, line 252 and Table 3 on page 14, line 259.

**#4.** Please define what the governments recommendations for minimum dietary diversity are in terms of food groups consumed within last 24 hours.

**Response:** Dear editor, thank you so much for your comments and we addressed the issue in the abstract on page 2, lines 16-19 and introduction section on page 4, lines 56-62.

**#5.** L 14: rather than ‘prevent’ I believe these recommendations/interventions help to ‘reduce’.

**Response:** Thank you for your comments, we revised the issue; see on page 2, line 14.

**#6.** L 14: I think this sentence need to be modified as diets have been ‘promoted’ and programs for the distribution MNPs or others supplements for iron and VA and deworming ‘have been implemented’.

**Response:** The comment is addressed, see on page 2, lines 14-16.

Methods
**#7.** L 21: ‘consumed within the previous’ 24 hours

**Response:** Thank you for your comments and the issue is incorporated; kindly see on page 2, lines 24-25.

**#8.** L 22: ‘supplementation with the previous’ seven days

**Response:** Thanks, the comment is incorporated; see on page 2, line 25.

Result
**#9.** L 31: This sentence seems unfinished. Are these factors that increase MN uptake or decrease MN intake, or mixed some increased and some decreased? Same comment for last sentence.
**Response:** thank you, we revised and addressed the issue; see on pages 2-3, lines 32-37.

**#10.** Conclusions L 35: recommendation’s’. it is still not clear what the Government recommendations are whether it is a diet with VA or iron rich foods plus VAS or iron supplementation or MNPs. I suspect you are trying to say diet rich (in VAS or iron) or VA or Iron supplementation or MNPs but it is still not clarified in Background.

**Response:** Dear editor, we are very much thankful for your comments and suggestions, we revised and addressed the issue abstract on page 2, lines 16-19 and introduction section on page 5, lines 68-74.

Introduction
**#11.** L 45: May lead would be better replaced by 'contribute to'

**Response:** Thanks, the comment is incorporated; see on page 4, line 49

**#12.** L 64: 6-59 months semi-annually in a (no need for ‘by’)

**Response:** The comment is addressed on page 5, line 71.

**#13**. L 65: Which ones? Iron folic acid (IFA) or multiple micronutrient supplements (MMS)?

**Response:** Thank you for your comments and it’s addressed on page 5, line 75.

**#14.** L 69: Were receiving ‘a diet of minimal dietary diversity’ Please don’t use an abbreviation such as MDD without first introducing it. What does the government recommend for MDD? Three or more of the food groups as elaborated further in Results? Please specify.

**Response:** Dear editor, thank you for the comment and the full term of MDD is first introduced on page 4, line 56. We clearly mentioned the government recommendation for MDD on page 5, line 72.

**#15**. L 82: Hotspot’s’ (pleural)

**Response:** The comment is addressed on page 6, line 92

**#16**. L 85: I think the last section of this sentence ‘that exacerbate MN deficiency’ could be deleted. These factors to not exacerbate MN deficiency they limit the ability to address the issue through interventions.

**Response:** Thanks, we deleted it; see on page 6, line 95.

**#17**. L 88: Finding’s’

**Response:** Addressed on page 6, line 98.

**#18.** L 89: Problem’s’
**Response:** The comment is addressed on page 6, line 99.

Study setting and data source
**#19.** L 104: ‘and access to health and education services’
**Response:** The comment is addressed on page 6, line 114.

Measurements of variables
**#20.** L 143: ‘consumed within the previous seven days’

**Response:** The comment is addressed on page 8, line 154-155.

**#21**. L 144: ‘the previous’ six months

**Response:** The comment is addressed on page 8, line 155.

**#22.** L 170: reviewers have asked previously how birth weight/size was assessed. Was it mothers recall of size or an actual weight taken at birth and recorded in a child health card? If it was mothers recall them, please use large, average or small as reported in table 3.

**Response:** The birth weight was assessed by mothers recall and classified as large, average or small and we addressed it on page 9, line 179.

**#23.** L 171: Where did you get this classification? 4kgs seem excessive. please double check and provide a reference. As the results of this weight/size was not fund to be of any significance it might be best to omit it altogether to avoid these critisims.

**Response:** Dear editor, thank you very much for your insights and we kept the birth weight since it is significant at the bivariable analysis and we reported using the mothers recall; small, average and large; kindly see on page 9, line 179.

**#24**. L 172: This section lines 172-187 is unclear. Sometime you use quintile, sometimes quantile and sometimes index. On line 175 you have 3 subdivisions and on line 181 you have 5 (usual for quintiles). please clarify.

**Response:** Dear editor, thank you for your comments and suggestions and we addressed the issue on pages 9-10, lines 180-192.

**#25**. L 174: Quintile or index?
**Response:** Thanks for the comment and it’s household wealth index and addressed on page 9, line 182.

Result
**#26.** L 231: What do to mean here wealth quintiles?

**Response:** Dear editor, thanks for the comments and we want to presented the household wealth index which is defined in the variables and measurement section of method; kindly refer the definition from line 180 to 192.

**#27.** L 241: Why have you written this in words rather than given the actual figure 56.4%?

**Response:** Dear editor, thank you for your comments. We believe that in a scientific writing it is better to use word if it comes at the beginning of the sentence rather than numeric value. We have addressed it now as per your suggestions; see on page 13, line 250.

**#28.** L 245 Table 2: What is the merit or presenting this data as it is not used in the further analysis or found significant. What is the merit of presenting all this date? It is not used or found significant in the analysis
What is the merit of presenting this data if it was not further analysis or found significant? A reader interested in these figures can find them in the EDHS. Only data that directed the reader to your important findings really deserves attention.

**Response:** Dear editor, we incorporate your suggestion and presented those significant variables in the bivariable analysis at a p-value of 0.2 to make easy for the readers; kindly see Table 2 on page 13, line 252.

**#29.** L 252: The merit of Table 3 is questionable. None of these factors were associated with MN intake.

**Response:** Dear editor, thank you for your observation. We presented those significant variables in the bivariable analysis at a p-value of 0.2 in table 3. We believe those significant variables at a p-value of 0.2 might give important information for further researcher and policy makers; kindly see Table 3 on page 14, line 259.

**#30.** L 253: Date that was not found to be of significance in the analysis could be removed from these tables to draw attention to elements there are of significance.
If the birth interval or birth order had no significant association with MN intake what is the merit of presenting the date?
what is the merit of the current child weight being presented if it is not related to the child age (W/A) or height (WH)? If you are not using this data in the analysis, please delete both these rows.
These last 3 section on diarhoea, cough and fever get not further mention in the analysis. If they were not significantly associated with MN intake, please delete these 6 rows and jut make a statement that there was not associated with MN intake.
**Response:** Dear editor, thank you for your suggestions and we presented those variables significant in the bivariable analysis and removed the others non-significant variables as per your suggestions; kindly see Table 3 on page 14, line 259.

Community level variables
**#31.** L 256: Past tense 'were'

**Response:** Thanks, and addressed the issue on page 14, line 263.

**#32**. L 257: Now you are using status as opposed to index, quintile/quantile.

**Response:** Thank you for your observation and we addressed the issue. We want to mention it as wealth quantile for measuring the community level poverty which is described in the method section on page 10, lines 187-192.

**#33.** L 259: Quantile needs a better explanation in methods
**Response:** Dear editor, thank you very much for your comments. We incorporate your comments and the is addressed; kindly see on page 10, lines 187-192.

Individual and community level factor of micronutrient intake status (fixed effects)
**#34.** L 293: Here you could list all the factors that were not significant.

**Response:** Dear editor, thank you for your comments and we incorporated the comments; kindly see on page 17-18, lines 300-304.

**#35.** L 301: Versus urban residents
**Response:** Thanks, and addressed the issue on page 17, line 312.

Discussion
**#36.** L 316-322 you are inconsistent with the use of decimal places sometime non and sometimes 2. For example: 38% and 43.17%. This is distracting for the reader.

**Response:** Thanks, and addressed the comments on page 21, lines 328-333.

**#37.** L 323: The term 'housewives' has not previously been used.

**Response:** Thank you for the comments and previously we used to express no job but now we omit the word and used no job alone; kindly see on page 21, line 334.

**#38.** L 347: Citing the author is not the PLOS ONE format for referencing
**Response:** Thanks, and we revised the citation as per the journal formatting; kindly see on page 22, lines 358-360.

Strength and limitation of the study
**#39.** L 367: It is normal practice to just draw attention to the study limitations as the last paragraph in discussion (without a sub-heading)
**Response:** Thank you for your suggestions, and we removed the subheading of strength and limitation of the study.
Conclusion
**#40.** L 375: You have not clearly defined the national dietary recommendations (number of food groups to be consumed in previous 24 hours). For deworming it might be >75% in the last 6 months. But what about the other options?

**Response:** Dear editor, thank you very much for your suggestions and we revised the national dietary recommendation; kindly see on page 4, lines 56-62 and page 5, lines 68-77.

**#41.** L 377: This second sentence is not based upon findings.

**Response:** Dear editor, thank you for your comments and we revised the conclusion; kindly see on page 23, lines 385-389.

**#42.** L 379-381: We have only cited deworming and MNPs. What about iron and VAS?

**Response:** Dear editor, thank you so much and we revised the issue; kindly see on page 23, lines 387-389.
