# Supplementary material for: Micronutrient intake status and associated factors among children aged 6–23 months in the emerging regions of Ethiopia: A multilevel analysis of the 2016 Ethiopia demographic and health survey
Source: PLoS One. 2021 Oct 22;16(10):e0258954. doi: 10.1371/journal.pone.0258954 (PMC8535338; doi:10.1371/journal.pone.0258954)
Supplement: Attachment — Submitted filename: Reviewer comments MB final.docx [file pone.0258954.s003.docx]

**Comments**

L 2: It is unclear what “emerging” means in this context. Please provide an explanation when this word is first introduced.

ABSTRACT

L13: Scarcity of MN is due to poor diets rather than poor health care access, health care access helps mitigate the impact of these poor diets but are not the fundamental cause.

L15: children 6-23 months include “and their associated factors”. Which MN? it is essential you specify what you are measuring is it Vitamin A supplementation or something else: MNPs, 24 dietary recall?

L 25: this is too generic, need to specify which ‘recommended micronutrient supplements’. If more than just VAS, what else?

L 30: Again, still unclear whether you are describing VAS or MNPs or 24 hour dietary recall.

L31: i suggest "study population in this area", rather than "study area"

L33: i suggest putting a full stop (.) after ‘community level’

L35: This final sentence is a recommendation, please conclude your findings rather than just saying they were low in the previous sentence.

INTRODUCTION

L45: I suggest “micronutrients are”

L50: I suggest “growth” instead of grow

L55: please define Minimal Diversified Diets (MDD)

L66: You need to explain more about VAD, is the 14% corrected for inflammation? If so which methods of correction was used. 14% prevalence is considered ‘moderate VAD’

L74: What is the definition of “emerging regions”. where are they on a map? Please provide a map.

L77: 'onset of' is superfluous

L81: “therefore” is unnecessary

L83: 'Moreover' is unnecessary

L84: I suggest “problems”

STUDY SETTINGS AND DATA SOURCES

L92: Why are they called 'emerging'. Please explain.

SAMPLING PROCEDURES

L108: I presume you mean 'one' segment rather than 'only a'.

L110: 'A fixed number of' is redundant. The sentence can start with 'twenty-eight'

L112: What do the enumerators do if no-one was at home to be interviewed? or no children 6-23 within the household.

L117: Why female survey, were females the only targeted audience?

L118: Please be consistent with the use of mothers/caregivers

L119: The meaning of this sentence is unclear.

L121: How was that one child of twins selected? By ballot?

L122: ‘extracted’ is an odd way of expressing this

MEASUREMENTS OF VARIABLES

L130: Again, the reader can’t follow the augment clearly as you haven't defined what MNs and which preparations and recommended timeframes at the beginning

L131: add “/caregivers”

L137: Please clearly explain in background why deworming medication was included in this analysis MNs

L138: is this referring to any one of the recommended micronutrients, two or three?

L159: Do the national programs start providing deworming medication at 6 months of age rather than more normally from 12 months of age. What was the deworming medication?

L160: How reliable is the child health card as a source of information rather than the verbal history form the caregiver.

L164: Do you mean obstetric or history and/or parity and/or ante-natal care?

L168: These indicators measured to reach your definition of wealth need to be described.

L169: Are these ranks comparable to international definitions or just in relation to the cohort under investigation. Are you really comparing very poor, poor, less poor?

L170-1: This sentence is unclear.

DATA PROCESSING AND STATISTICAL ANALYSIS

L185: Again, do you mean parity/access to ANC?

RESULTS

L223: Again, were **ALL** the participants mothers of these children or were some caregivers?

OBSTETRIC HISTORY OF PARTICIPANT

L236: please define the term “multipara”

L237: acronym ANC not yet introduced and must be spelt in fill the first time it is used

L239: acronym PNC not yet introduced and must be spelt in fill the first time it is used

L241: Grand multipara must first be defined as mentioned above

CHILD CHARACTERISTICS AND COMMON CHILDHOOD ILLNESSES

L245: No definitions of average birth weight provided

L247: “were weighted” should be changed to 'were weighed'

L247: please insert % after these figures

L248: I presume you mean had 'either' rather than 'and'

L251: I suggest adding “within last 2 weeks” for diarrhoea, cough and fever which is I believe the timeframe under consideration

COMMUNITY LEVEL POVERTY, MEDIA EXPOSURE AND ACCESS TO HEALTH FACILITY

L254: ‘had had’ not ‘has’

L256: do you mean ‘was’ or ‘was not’

MICRONUTRIENT INTAKE AMONG CHILDREN 6-59 MONTH

L258: I don't think you adequately described your definition of minimum recommended in background or methods.

DISCUSSION

I think this section need to be carefully rewritten to ensure it relates to the findings in a systematic manner.

L361: discuss the association to “care” in these regions. like active response feeding for this age group since they are young compare to the older age group, who might be considered “can eat for themselves” in a family meal!!

CONCLUSIONS

L418-onwards are recommendations rather than conclusions based upon findings

REFERENCES

Placed ensure you comply with instructions to authors.
